# Core Scientific Dataset Model: A lightweight and portable model and file format for multi-dimensional scientific data

**DOI:** 10.1371/journal.pone.0225953

**Published:** 2020-01-02

**Authors:** Deepansh J. Srivastava, Thomas Vosegaard, Dominique Massiot, Philip J. Grandinetti

**Affiliations:** 1 Department of Chemistry, Ohio State University, 100 West 18th Avenue, Columbus, OH 43210, United States of America; 2 Laboratory for Biomolecular NMR Spectroscopy, Department of Molecular and Structural Biology, University of Aarhus, DK-8000 Aarhus C, Denmark; 3 CEMHTI UPR3079 CNRS, Univ. Orléans, F-45071 Orléans, France; University of Michigan, UNITED STATES

## Abstract

The Core Scientific Dataset (CSD) model with JavaScript Object Notation (JSON) serialization is presented as a lightweight, portable, and versatile standard for intra- and interdisciplinary scientific data exchange. This model supports datasets with a *p*-component dependent variable, {**U**_0_, …, **U**_*q*_, …, **U**_*p*−1_}, discretely sampled at *M* unique points in a *d*-dimensional independent variable (**X**_0_, …, **X**_*k*_, …, **X**_*d*−1_) space. Moreover, this sampling is over an orthogonal grid, *regular* or *rectilinear*, where the principal coordinate axes of the grid are the independent variables. It can also hold correlated datasets assuming the different physical quantities (dependent variables) are sampled on the same orthogonal grid of independent variables. The model encapsulates the dependent variables’ sampled data values and the minimum metadata needed to accurately represent this data in an appropriate coordinate system of independent variables. The CSD model can serve as a re-usable building block in the development of more sophisticated portable scientific dataset file standards.

## 1 Introduction

A frustrating and common problem faced by scientists in many disciplines is the lack of a portable scientific dataset format and universal standards for exchanging and archiving multi-dimensional datasets—both experimental and computational. Scientific datasets are too often saved in vendor-specific file-formats using proprietary software, making archiving and data-exchange problematic even within a discipline, let alone across disciplines. A majority of scientists rely on vendor-specific proprietary software to interact with their datasets. These scientists are at a constant risk that the original dataset files could become unreadable if a future version of the software stops supporting older file formats or the vendor stops supporting the software, or even worse, goes out of business.

As a result of such risks and incompatibilities, many scientists resort to using comma-separated values (CSV) files for dataset exchange and archival. Such an approach, however, is not resourceful, especially in the case of multi-dimensional datasets. Furthermore, such approaches often leave out essential metadata about experimental or computational procedures. Other scientists resort to specialized library packages to import datasets from the vendor-specific file formats into their favorite programming languages such as Matlab, Python, R, Java, or use the third-party software for dataset imports. This is only a temporary fix since it just delays the original problem as the dataset files are translated to yet another third-party software or user-specific file-format—and again, often with metadata loss.

With increasing pressure from the funding agencies and scientific journals to archive and share primary and processed data, there is a growing sense of urgency for a stable, resourceful and future-proof file-format for the exchange of scientific datasets. Here we take the first step in addressing this problem by proposing a *Core Scientific Dataset (CSD) Model* that can encode a wide variety of multi-dimensional and correlated datasets. The objective of the CSD model is to encapsulate the *data values* and the *minimum metadata* needed to accurately represent the data in an appropriate coordinate system. We envision the CSD model as a re-usable building block in a hierarchical description of more sophisticated portable scientific dataset file standards.

## 2 Overview of CSD model

The CSD model supports a dataset of a continuous physical quantity (dependent variable) discretely sampled on a multi-dimensional grid with vertexes associated with one or more independent quantities (dimensions), e.g., a density as a function of temperature, a current as a function of voltage and time, an ionization energy as a function of element symbol, etc.

Similarly, the CSD model supports a dataset with a multi-component dependent variable. For example, a color image with a red, green, and blue (RGB) light intensity components as a function of two independent spatial dimensions, or the six components of the symmetric second-rank diffusion tensor MRI dataset as a function of three independent spatial dimensions. In the CSD model, a *dataset* is defined as an *p*-component dependent variable, {**U**_0_, …, **U**_*q*_, …, **U**_*p*−1_}, discretely sampled at *M* unique points in a *d*-dimensional (**X**_0_, …, **X**_*k*_, …, **X**_*d*−1_) space. Moreover, this sampling is over an orthogonal grid, *regular* or *rectilinear*, where the principal coordinate axes of the grid are the dimensions. A regular grid is an orthogonal grid where the spacing between vertex coordinates along each dimension is uniform. If the spacing along any one of the dimensions is not uniform, the grid is rectilinear.

The CSD model can also hold multiple datasets when different physical quantities (dependent variables) are sampled on the same multi-dimensional (independent variables) grid. We refer to this case as *correlated datasets*. One such example would be the simultaneous sampling of current and voltage as a function of time. Another example would be datasets for air temperature, pressure, wind velocity, and solar-flux, all simultaneously sampled on a two-dimensional grid associated with the same region of latitude and longitude coordinates.

We adopt the JavaScript Object Notation (JSON) as the file-serialization format [1] for the CSD model because it is human-readable, if properly organized, as well as *easily integrable* with any number of programming languages and field related application-software.

### 2.1 UML class diagram

**Fig 1:**
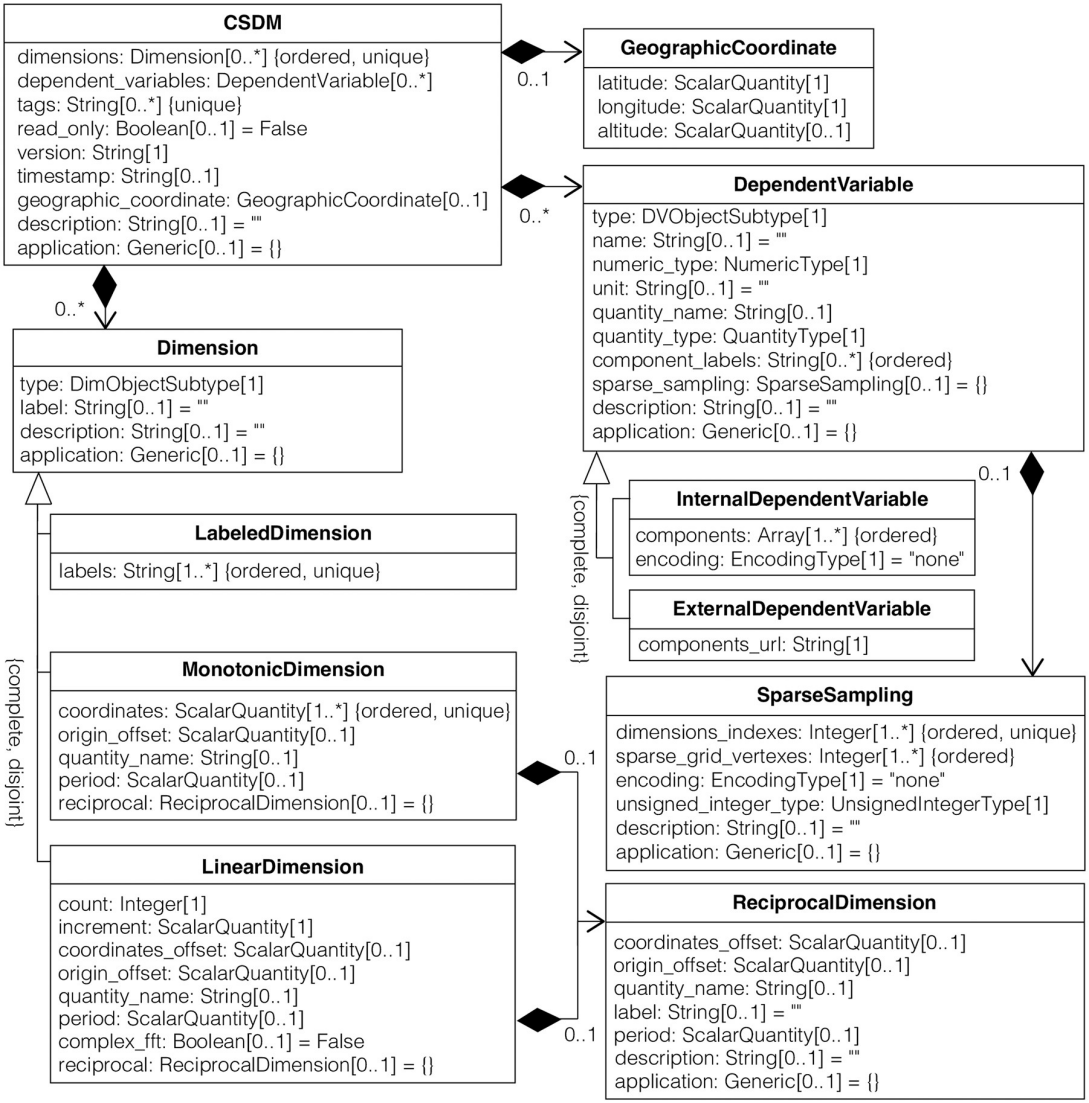
Unified Modeling Language (UML) [2] class diagram of the Core Scientific Dataset (CSD) Model. Each class is represented with a box that contains two compartments. The top compartment contains the name of the class, and the bottom compartment contains the attributes of the class. The enumerations DimObjectSubtype and DVObjectSubtype are described in Tables 1 and 2 as the description of the type attribute. The enumerations QuantityType, NumericType, and EncodingType are described in Tables 3, 4 and 5, respectively. The enumeration UnsignedIntegerType is a subset of NumericType enumeration with only unsigned integers. The **ScalarQuantity** represents a physical quantity containing a numerical value and a unit. Note: When encoding is base64 the type and multiplicity for the components attribute in **InternalDependentVariable** is String[1..*]. Similarly, when encoding is base64 the type and multiplicity for the sparse_grid_vertexes attribute in **SparseSampling** is String[1].

The schema for the CSD model, in the form of a UML class diagram [2], is shown in Fig 1. In such diagrams, each class is represented with a box that contains two compartments. The top compartment contains the name of the class, and the bottom compartment contains the attributes of the class. A composition is depicted as a binary association decorated with a filled black diamond. Inheritance is shown as a line with a hollow triangle as an arrowhead.

**Table 1:** The description of the attributes from the Dimension class in version 1.0 of the CSD model.

Dimension	
attribute	description
type	*Required* attribute for all **Dimension** objects. Holds a **String** object with one of the allowed **DimObjectSubtype** enumeration literals—linear, monotonic or labeled.
labels	*Required* attribute for **LabeledDimension** objects. Holds an *ordered and unique array* of **String** objects containing UTF-8 allowed characters. *Invalid* for **LinearDimension** and **MonotonicDimension** objects.
coordinates	*Required* attribute for **MonotonicDimension** objects. Holds an *ordered and unique array* of strictly increasing or decreasing **ScalarQuantity** objects along the dimension. The dimensionality of **ScalarQuantity** objects must be consistent with each other and other dimension attributes. *Invalid* for **LinearDimension** and **LabeledDimension** objects.
count	*Required* attribute for **LinearDimension** objects. Holds an **Integer** object specifying the number of coordinates, *N*_*k*_, along the dimension. *Invalid* for **MonotonicDimension** and **LabeledDimension** objects.
increment	*Required* attribute for **LinearDimension** objects. Holds a **ScalarQuantity** object specifying the increment, Δ*x*_*k*_, along the dimension. *Invalid* for **MonotonicDimension** and **LabeledDimension** objects.
coordinates_offset	*Optional* attribute for **LinearDimension** objects. Holds a **ScalarQuantity** object specifying the coordinates offset, *b*_*k*_, used in Eq (3) to calculate the coordinates along the dimension. The default value is a physical quantity with a numerical value of zero. *Invalid* for **MonotonicDimension** and **LabeledDimension** objects.
origin_offset	*Optional* attribute for **LinearDimension** and **MonotonicDimension** objects. Holds a **ScalarQuantity** object specifying the origin offset, *o*_*k*_, along the dimension. The default value is a physical quantity with a numerical value of zero. *Invalid* for **LabeledDimension** objects.
complex_fft	*Optional* attribute for **LinearDimension** objects. Holds a **Boolean** specifying how the coordinate, **B**_*k*_, along the dimension are calculated from Eq (3). When false, the value of *Z*_*k*_ = 0 otherwise, *Z*_*k*_ = *T*_*k*_/2 where *T*_*k*_ = *N*_*k*_ and *N*_*k*_ − 1 for even and odd values of *N*_*k*_, respectively. *Invalid* for **MonotonicDimension** and **LabeledDimension** objects.
period	*Optional* attribute for **MonotonicDimension** and **LinearDimension** objects. Holds a **ScalarQuantity** object specifying the period of the dimension. The default value is a physical quantity with an infinite numerical value, that is, the absence of this key indicates that the dimension is non-periodic. When present it indicates that all dependent variables are periodic along the dimension. A **ScalarQuantity** object with a numerical value of zero is *invalid* for this attribute. *Invalid* for **LabeledDimension** objects.
quantity_name	*Optional* attribute for **MonotonicDimension** and **LinearDimension** objects. Holds a **String** object containing the *quantity name* associated with the dimension. This value may resolve ambiguities which may otherwise be inherent. For example, with only a unit of “J/(mol*K)”, one cannot distinguish between the thermodynamic quantities ‘molar entropy’ and ‘molar heat capacity.’ Similarly, the units “1/s”, “Bq”, and “Hz” all have the dimensionality of inverse time, but generally “Bq” would be an acceptable unit for the quantity of radioactivity and “Hz” for frequency. If unspecified the valid quantity name is left at the end-user’s discretion. A list of CSDM-accepted physical quantity names and their corresponding dimensionalities can be found in the supporting information. *Invalid* for **LabeledDimension** objects.
label	*Optional* attribute for all **Dimension** objects. Holds a **String** object of UTF-8 allowed characters containing the label for the **Dimension** object. The default value is an empty string.
description	*Optional* attribute for all **Dimension** objects. Holds a **String** object of UTF-8 allowed characters describing an instance of the **Dimension** object. The default value is an empty string.
reciprocal	*Optional* attribute for **MonotonicDimension** and **LinearDimension** objects. Holds a **ReciprocalDimension** object. See Fig 1 for the list of attributes in this object. These attributes follow the same definitions as described in this Table with the only difference being that these attributes describe the reciprocal dimension. *Invalid* for **LabeledDimension** objects.
application	*Optional* attribute for all **Dimension** objects. Holds a generic dictionary object. See section 2.5 for expected behavior.

There are three subtypes of this class—**LinearDimension**, **MonotonicDimension**, and **LabeledDimension**. See Fig 1 for the list of *valid* attributes for a given subtype. If an attribute is optional, its value should only be serialized to the file if it is not the default value. As a recommendation, when deserializing a JSON file the numerical value associated with the physical quantities should be converted to a 32-bit or higher floating-point number.

**Table 2:** The description of the attributes from the DependentVariable class in version 1.0 of the CSD model.

DependentVariable	
attribute	description
type	*Required* attribute for all **DependentVariable** objects. Holds a **String** object with one of the two allowed **DVObjectSubtype** enumeration literals—internal or external.
components	*Required* attribute for **InternalDependentVariable** objects. Holds an *ordered array* of *p* components. When the value of encoding attribute is none each component, **U**_*q*_, is an *ordered array* of numerical values. When the value of the encoding attribute is base64, each component is a Base64 string. *Invalid* for **ExternalDependentVariable** objects.
components_url	*Required* attribute for **ExternalDependentVariable** objects. Holds a **String** object containing the Uniform Resource Locator (URL) of a local or a remote file where the ordered array of numerical values {**U**_0_, …,**U**_*q*_, …, **U**_*p*_} are stored as binary data. The CSD model utilizes the https and file schemes for locating the files. For local data files, the URL is specified relative to the .csdfe file and is located either in the folder containing the .csdfe file or in a subfolder of the folder containing the .csdfe file. The corresponding syntax follows file:./relative/path/to/the/file. *Invalid* for **InternalDependentVariable** objects.
quantity_type	*Required* attribute for all **DependentVariable** objects. Holds a **String** object with any of the allowed **QuantityType** enumeration literals from Table 3. The value specifies the number, *p*, and interpretation of the **DependentVariable** components.
numeric_type	*Required* attribute for all **DependentVariable** objects. Holds a **String** object with one of the allowed **NumericType** enumeration literals from Table 4. This value represents the numeric type and the number of bits associated with each numerical value in Eq (5) when the component data is stored in an external file or when Base64 encoded into a string. When numerical values are expressed as JSON numbers, this value specifies the numerical precision needed for import.
unit	*Optional* attribute for all **DependentVariable** objects. Holds a **String** object representing the unit associated with the data values in Eq (5). The default value is “”, i.e., the data values are dimensionless.
quantity_name	*Optional* attribute for all **DependentVariable** objects. Holds a **String** object containing the *quantity name* associated with the physical quantity. See the description for the quantity_name attribute in Table 1 for further details.
encoding	*Optional* attribute for **InternalDependentVariable** objects. Holds a **String** object with one of the allowed **EncodingType** enumeration literals in Table 5. This value specifies the encoding method used to store the data values in the components attribute. The default value is none. *Invalid* for **ExternalDependentVariable** objects.
component_labels	*Optional* attribute for all **DependentVariable** objects. Holds an *ordered array* of **String** objects where the *q*^th^ **String** is the label associated with the *q*^th^ component. The default value is an ordered set of empty strings.
name	*Optional* attribute for all **DependentVariable** objects. Holds a **String** object of UTF-8 allowed characters containing the name associated with the dependent variable. Naming is good practice as it improves the human readability of the serialized file when multiple dependent variables might be present. The default value is an empty string.
description	*Optional* attribute for all **DependentVariable** objects. Holds a **String** object of UTF-8 allowed characters describing an instance of the **DependentVariable**. The default value is an empty string.
sparse_sampling	*Optional* attribute for all **DependentVariable** objects. Holds a **SparseSampling** object, which contains the attributes dimension_indexes and sparse_grid_vertexes. The attribute dimension_indexes holds an array of integers indicating which dimensions in the ordered array of dimensions are sparsely sampled and form the sparse grid. The attribute sparse_grid_vertexes holds an array of integers defining the ordered set of sampled vertexes on the sparse grid. See section 2.4.1 for further details.
application	*Optional* attribute for all **DependentVariable** objects. Holds a generic dictionary object. See section 2.5 for expected behavior.

If an attribute is optional, its value may only be serialized to the file if it is not the default value.

**Table 3:** QuantityType enumeration literals allowed in version 1.0 of the CSD model.

literals	description
scalar	This value represents a *p* = 1, single-component dependent variable where the *i*^th^ data value is interpreted as a scalar value, $S_{i}=U_{0,i}$.
vector_*n*	The value represents a *p* = *n* component dependent variable where the *i*^th^ data value is interpreted as a vector, $V_{i}=[U_{0,i},U_{1,i},\ldots U_{n-1,i}]$.
matrix_*m*_*n*	The value represents a *p* = *mn* component dependent variable where the *i*^th^ data value is interpreted as a *m* × *n* matrix, with *m* rows and *n* columns. The *p* components of the matrix are in column-major order. $$\begin{array}{c} M_{i}=[\begin{array}{cccc} U_{0,i} & U_{m,i} & \ldots & U_{(n-1)m,i}\\ U_{1,i} & U_{m+1,i} & \ldots & U_{(n-1)m+1,i}\\ \vdots & \vdots & \vdots & \vdots \\ U_{m-1,i} & U_{2m-1,i} & \ldots & U_{nm-1,i} \end{array}]. \end{array}$$ Here, the entry at the *r*^th^ row and the *c*^th^ column is *U*_*cm*+*r*,*i*_.
symmetric_matrix_*n*	The value represents a $p=\frac{n(n+1)}{2}$ component dependent variable. This is a special case of matrix data value where *n* = *m* and the matrix is symmetric about the leading diagonal. In this case, only the upper half of the matrix is specified. The *n* × *n* symmetric matrix, $M_{i}^{\left(s\right)}$, of the *i*^th^ data value is interpreted as, $$\begin{array}{c} M_{i}^{\left(s\right)}=[\begin{array}{cccc} U_{0,i} & U_{1,i} & \ldots & U_{n-1,i}\\ U_{1,i} & U_{n,i} & \ldots & U_{2n-2,i}\\ \vdots & \vdots & \vdots & \vdots \\ U_{n-1,i} & U_{2n-2,i} & \ldots & U_{\frac{n(n+1)}{2}-1,i} \end{array}]. \end{array}$$
pixel_*n*	The value represents a *p* = *n* component dependent variable where the *i*^th^ data value is interpreted as a pixel, [*U*_0,*i*_, *U*_1,*i*_, …, *U*_*n*−1,*i*_], with the *n* components corresponding to pixel component intensities. Note this quantity type, as do all quantity types, is restricted to components that share the same physical dimensionality, i.e., can be added or subtracted, making it, for example, appropriate for holding RGB or CMYK components but not HSV components.

The literals are **String** objects and correspond to the value of the quantity_type attribute of the **DependentVariable** object. In the description, the index *i* refers to the *i*^th^ data value from the ordered array, **U**_*q*_, in Eq (5).

**Table 4:** NumericType enumeration literals allowed in version 1.0 of the CSD model.

literals	description
uint8	8-bit unsigned integer
uint16	16-bit unsigned integer
uint32	32-bit unsigned integer
uint64	64-bit unsigned integer
int8	8-bit signed integer
int16	16-bit signed integer
int32	32-bit signed integer
int64	64-bit signed integer
float32	32-bit floating-point number
float64	64-bit floating-point number
complex64	two 32-bit floating-points numbers
complex128	two 64-bit floating-points numbers

The literal is a **String** object corresponding to the value of the numeric_type attribute of the **DependentVariable** object.

**Table 5:** EncodingType enumeration literals allowed in version 1.0 of the CSD model.

literals	description
base64	The binary data corresponding to the ordered array of numerical values in **U**_*q*_ from Eq (5) is stored as a Base64 encoded strings for the numeric_type specified assuming ‘little-endian’ format. This is the recommended storage method when the type attribute of the corresponding **DependentVariable** object is internal.
none	The literal denotes that the ordered array of numerical values from Eq (5) are serialized as JSON numbers. This is the default encoding type when the encoding attribute is not present in the **DependentVariable** object.

The literals are the **String** object corresponding to the value of the encoding attribute of the **DependentVariable** object.

Each line in the bottom compartment of a box describes a single attribute of the class in the form:

name:type[multiplicity]=default{properties}

In this line name is the name of an attribute in the class, type defines the kind of object that may be placed in the attribute, multiplicity indicates how many objects are assigned to the attribute. The multiplicity can be a single number, e.g., “[1]”, indicating that one object must be assigned to the attribute. Alternatively, the multiplicity can be given as a lower and upper bound for how many objects can be assigned to the attribute, e.g., “[0..1]” indicates that the assignment of a single object to an attribute is optional. An asterisk indicates an unlimited number of objects. For example, an attribute with a multiplicity of “[1..*]” must have no less than one object and an unlimited upper bound of objects that can be assigned to it. The default is the object assigned when an optional attribute is unspecified. The {properties} value at the end of the line gives additional information on the attribute. In Fig 1 this is used to indicate whether a set of objects assigned to an attribute is ordered and/or unique.

For object attribute names we adopt the “snake case” convention with all lower case characters and “camel case” for class or type names. Attribute value types used in the model are given in Table 6 along with the corresponding JSON value type used for serialization of the model. Of particular importance in the CSD model is the **ScalarQuantity** type, which is composed of a numerical value and any valid SI unit symbol or any number of accepted non-SI unit symbols. It is serialized in the JSON file as a string containing a numerical value followed by the unit symbol, for example, “3.4 m” (SI) or “2.3 bar” (non-SI). The CSD model follows the International System of Units guideline [3] for defining the physical quantities. In software usage, one must adhere to stricter conventions for unit and physical constant symbols to avoid ambiguities and symbol collisions. All unit symbols are case sensitive. For derived unit symbols, the multiplication and division of the units are represented by the *asterisk* symbol, “*”, and the *solidus* symbol, “/”, respectively. For example, a unit of speed is “m/s”. Note that derived unit symbols in the CSD model require explicit use of the multiplication symbol instead of multiplication implied with spacing between symbols, e.g., use “N*m” instead of “N m”. Similarly, avoid the use of compound symbols, e.g., use “kW*h” instead of “kWh”. The *caret* symbol, “^” is used for raising unit symbols to a power—a unit of force is “kg*m^2/s^2”, and a unit of concentration is “g/cm^3”. Operator precedence can be specified using parentheses, e.g., “J/(mol*K)”. Also, note that while both °C and °F are valid units, they are not proper thermodynamic temperature units and are discouraged due to their ambiguity. Further details on the SI system and how units are used in the CSD model are given in the supporting information.

**Table 6:** Mapping of CSD model attribute values to JSON serialized values.

CSD model attribute value type	JSON value type
**DependentVariable**	object
**Dimension**	object
**DimObjectSubtype**	string
**DVObjectSubtype**	string
**ScalarQuantity**	string
**NumericType**	string
**EncodingType**	string
**QuantityType**	string
**String**	string
**Integer**	number
**Boolean**	boolean

The relation between the CSD model attribute value types and the corresponding JSON serialized value type. In JSON serialization the attribute name is the JSON key.

### 2.2 CSDM object

At the root level of the CSD model is the **CSDM** object. The **CSDM** object includes a required version attribute whose value is a string representing the version number of the CSD model, here assigned a string value of “1.0”. The optional timestamp attribute indicates when the CSDM file was last serialized and holds a combined date and time string representation of the Coordinated Universal Time (UTC) formatted according to the ISO-8601 standard. The optional geographic_coordinate attribute indicates where the CSDM file was last serialized and holds a **GeographicCoordinate** object, inside which are three attributes: the required latitude and longitude, and the optional altitude. Positive latitude values indicate latitudes north of the equator, while negative values indicate latitudes south of the equator. Longitude values are relative to the zero meridian, with positive values extending east of the meridian and negative values extending west of the meridian. Positive altitudes indicate above sea level while negative values indicate below sea level. All three are **ScalarQuantity** types. The optional boolean read_only attribute is set to true for archived datasets—informing applications that the dataset should not be modified or overwritten. The optional tags attribute holds a set of UTF-8 allowed string values describing keywords associated with the dataset. The description attribute appears in nearly every CSD model object and holds a UTF-8 allowed string describing the instance of the model object. The application attribute also appears in nearly every CSD model object and is a generic object that can be used for storing application-specific metadata within the CSD model. Further details on the expected behavior of application attributes are given in section 2.5.

The dependent_variables and dimensions attributes each hold a set of **DependentVariable** and **Dimension** objects, respectively. The ordered and unique set of **Dimension** objects, indexed from *k* = 0 to *d* − 1, define the *d*-dimensional coordinate grid where discrete samples of the dependent variables are taken.

### 2.3 Dimension object

The mapping of grid vertexes along the *k*^th^ dimension to an ordered set of coordinates, **X**_*k*_, are defined by one of three **Dimension** subtypes: **LabeledDimension**, **MonotonicDimension**, and **LinearDimension**. Fig 1 gives the required and optional attributes along with their default values for the three subtypes. Descriptions of the attributes for all three subtypes are also given in Table 1, and examples of various instances are given in section 3.

#### 2.3.1 LabeledDimension object

An ordered set, **A**_*k*_, of *N*_*k*_ character string labels in the labels attribute of a **LabeledDimension** object are mapped to the grid vertexes along the *k*^th^ dimension, becoming the ordered set of coordinates, **X**_*k*_, along the dimension, as given by

$$\begin{array}{c} X_{k}=A_{k}. \end{array}$$ (1)

This is a purely qualitative dimension, with no physical significance given to the spacing between grid vertexes along the dimension.

#### 2.3.2 MonotonicDimension object

An ordered set, **A**_*k*_, of *N*_*k*_ strictly ascending or descending coordinates in the coordinates attribute of a **MonotonicDimension** object are similarly mapped to the grid vertexes along the *k*^th^ dimension and become the ordered set of coordinates along the dimension, also given by Eq (1).

For the **MonotonicDimension** and **LinearDimension** objects, the CSD model allows the mapping of grid vertexes along a dimension to an ordered set of absolute coordinates, $X_{k}^{\text{abs}}$, using the origin_offset attribute according to

$$\begin{array}{c} X_{k}^{\text{abs}}=X_{k}+o_{k}1, \end{array}$$ (2)

where *o*_*k*_ is the value of the origin_offset attribute. Note, the **ScalarQuantity** objects in **X**_*k*_, and *o*_*k*_ must all share the same unit dimensionality.

#### 2.3.3 LinearDimension object

The ordered set of *N*_*k*_ uniformly spaced coordinates along the *k*^th^ **LinearDimension** object are given by

$$\begin{array}{c} X_{k}=\Delta x_{k}\;\left(J_{k}-Z_{k}\right)+b_{k}1, \end{array}$$ (3)

where Δ*x*_*k*_ and *b*_*k*_ are the **ScalarQuantity** objects in the increment and coordinates_offset attributes, respectively, and **J**_*k*_ is an ordered set of coordinate indexes along the *k*^th^ dimension,

$$\begin{array}{c} J_{k}=[0,1,2\ldots ,j_{k},\ldots ,N_{k}-1]. \end{array}$$ (4)

Here, *N*_*k*_ is the **Integer** object in the count attribute. As before, the absolute coordinates along the *k*^th^ dimension are given by Eq (2). Again, the **ScalarQuantity** objects Δ*x*_*k*_, *b*_*k*_, and *o*_*k*_ must all share the same unit dimensionality.

The *Z*_*k*_ variable in Eq (3) is an integer with a value of *Z*_*k*_ = 0 when the **LinearDimension** attribute complex_fft is false. The complex_fft is set to true when a complex fast Fourier transform (FFT) has been applied to the dataset along the *k*^th^ dimension, and then the value of *Z*_*k*_ becomes *T*_*k*_/2, where *T*_*k*_ = *N*_*k*_ and *N*_*k*_ − 1 for even and odd values of *N*_*k*_, respectively. There are two reasons for the inclusion of the attribute complex_fft and the different values of *Z*_*k*_. First, it provides the metadata needed for determining whether a forward (false) or reverse (true) complex FFT should be performed on the dataset. Second, a value of *Z*_*k*_ = *T*_*k*_/2 in Eq (3) when complex_fft is true associates *b*_*k*_ with the zero “frequency” after a complex FFT. This definition makes *b*_*k*_ independent of count and the increment in the Reciprocal dimension, i.e., the dimension before the complex FFT.

#### 2.3.4 ReciprocalDimension object

An optional attribute named reciprocal can be present in both the **LinearDimension** and **MonotonicDimension** objects. This attribute holds a **ReciprocalDimension** object which contains metadata about the coordinate that is reciprocal to the **X**_*k*_ coordinate. This metadata is useful for datasets which are frequently transformed into the reciprocal dimension, such as NMR, FTIR and x-ray datasets.

### 2.4 DependentVariable object

The **DependentVariable** object can be one of two subtypes: **InternalDependentVariable** and **ExternalDependentVariable**, depending on whether the serialized components are stored internally with the rest of the serialized metadata or externally at a location specified by a uniform resource locator (URL) [4], respectively. Descriptions of all **DependentVariable** attributes are given in Table 2, as well as through examples given in section 3. See Fig 1 for the required and optional attributes along with their default values.

A **DependentVariable** object holds an ordered set of *p* components indexed from *q* = 0 to *p* − 1,

$$\begin{array}{c} \{U_{0},\ldots ,U_{q},\ldots ,U_{p-1}\}. \end{array}$$ (5)

Each component, **U**_*q*_, contains an ordered array of *M* physical quantity values indexed from *i* = 0 to *M* − 1. These values represent samples on the coordinates grid and are ordered to follow a *column-major order* relative to the ordered set of dimensions. If **U**_*q*_ contains a sample at every vertex of the *d*-dimensional grid, then

$$\begin{array}{c} M=\prod_{k=0}^{d-1}N_{k}, \end{array}$$ (6)

and the mapping of the values in **U***_q_* to the grid vertexes follows a simple reshaping of **U***_q_* to a *N*_0_ × *N*_1_ × … × *N*_*d*−1_ matrix where *d* is the number of **Dimension** objects. In this case, the location or memory offset of the *i*^th^ value in a component array maps to a grid vertex with coordinate indexes, (*j*_0_, *j*_1_, …, *j*_*d*−1_), given by

$$\begin{array}{c} j_{k}=\frac{i}{\prod_{\ell =0}^{k-1}N_{\ell }}\;\mathrm{mod}\;N_{k},\;\;\;\;\;k=0,\ldots ,d-1. \end{array}$$ (7)

Conversely, the memory offset of the *i*^th^ value in a component array is obtained from the ordered array of coordinate indexes (*j*_0_, *j*_1_, …, *j*_*d*−1_), according to

$$\begin{array}{c} i=\sum_{k=0}^{d-1}(\prod_{l=0}^{k-1}N_{l})j_{k}. \end{array}$$ (8)

It is also helpful to recall that the value of the empty product, $\prod_{m}^{n}a_{m}$ where *m* > *n* is 1.

Taken together, the *i*^th^ values from each of the *p* components form a quantity specified by one of the quantity_type attribute values given in Table 3.

#### InternalDependentVariable

The components attribute in an **InternalDependentVariable** object holds an *ordered array* of *p* components, and each component, **U**_*q*_, is an *ordered array* of *M* numerical values associated with the *q*^th^ component. When the value of the encoding attribute is none or unspecified, a JSON serialization of this object gives a human-readable list of numerical values. This approach, however, is not resourceful compared to the serialization of raw binary data. As JSON files are strictly text-based it is not possible to serialize raw binary data inside a JSON file. A commonly used approach to reduce JSON file sizes in such situations is to encode raw binary data into plain text using a binary-to-text encoding scheme. The CSD model allows this approach with the raw binary data for each component encoded into a Base64 string when the encoding attribute is set to base64. In this case, JSON serialization of the components attribute in an **InternalDependentVariable** object holds an *ordered array* of *p* Base64 strings where the *q*^th^ string represents the array **U**_*q*_. Out of the various binary-to-text encoding schemes, we chose Base64 encoding because of its widespread use and easy access to decoders across most object-oriented programming languages. Base64 provides an efficiency of ∼75% compared to the serialization of raw binary data. When encoding and decoding raw binary data with Base64 we assume a ‘little-endian’ byte order for multi-byte numeric types such as 32-bit and 64-bit integers or floats. Typically, data saved on Intel x86 platforms use the little-endian as the native format. Also, binary floating-point standard IEEE 754 is assumed for float and complex numeric types.

#### ExternalDependentVariable

The components_url attribute is only *valid* when the value of the corresponding type attribute is external. Its value is a **String** object containing the address of a local or a remote file where the ordered array of numerical values {**U**_0_, …,**U**_*q*_, …, **U**_*p*_} are stored as binary data. In this case we also assume little-endian byte order and the binary floating-point standard IEEE 754 for float and complex numeric types. The CSD model utilizes the https and file schemes of the Uniform Resource Locator (URL) for locating the files. For local data files, the URL is specified relative to the .csdfe file (*see section 2.6*) and is located either in the folder containing the .csdfe file or in a subfolder of the folder containing the .csdfe file. The corresponding syntax follows, file:./relative/path/to/the/file.

#### 2.4.1 SparseSampling object

Eqs (6), (7) and (8) are no longer valid when the **DependentVariable** components are sparsely sampled on the *d* dimensional grid. In this case, additional metadata is required to determine the grid vertex, (*j*_0_, *j*_1_, …, *j*_*d*−1_), where the *i*^th^ sampled component value belongs. If the component is sparsely sampled along all *d* dimensions, then the additional metadata can be an ordered set of *M* grid vertexes. We must, however, consider the general mixed case of *s* fully sampled dimensions and *d* − *s* sparsely sampled dimensions. In this case, we adopt an approach where the component values are organized into a set of fully sampled *s*-dimensional cross-sections taken at vertexes of a sub-grid formed from the sparsely sampled dimensions, which we will call the *sparse grid*. In adopting this approach, we require that the *component values along the fully sampled dimensions are packed together into the array in column-major order relative to the ordered set of fully sampled dimensions, i.e., excluding the sparsely sampled dimensions*.

The **SparseSampling** object provides this metadata in its two attributes dimension_indexes and sparse_grid_vertexes. The dimension_indexes attribute holds an ordered and unique set of integers indicating along which dimensions the **DependentVariable** is sparsely sampled. These dimensions form the sparse grid. The sparse_grid_vertexes holds an ordered set of vertexes on the sparse grid. Each sparse grid vertex is an ordered array of *d* − *s* indexes. To make the serialization more resourceful, we flatten the ordered set of arrays intended for the sparse_grid_vertexes attribute into an ordered array of integers, for example,

$$\begin{array}{c} {}[[1,0],[3,4],[5,7],[8,11],\ldots ]\to [1,0,3,4,5,7,8,11,\ldots ], \end{array}$$

in a case of two sparsely sampled dimensions. The set of arrays (on the left) can be easily reconstructed from the array of integers (on the right) given the number of indexes specified in the dimension_indexes attribute. Additional storage reduction can be had by encoding the sparse_grid_vertexes array as a Base64 character string of specified unsigned_integer_type and little-endian byte ordering. The encoding attribute in the **SparseSampling** object would indicate this option with a value of base64.

### 2.5 Generic application objects—Beyond the CSD model

As stated earlier, the objective of the CSD model is to encapsulate the *data values* and the *minimum metadata* needed to accurately represent the data in an appropriate coordinate system, that is, the minimum metadata for defining the current state of the dataset. Thus, the goal of the CSD model is to always remain relevant as the state of the dataset changes. In our refinement of the CSD model, we identified any metadata attribute as extraneous if it could become irrelevant as the state of the dataset changes. Metadata attributes extraneous to the CSD model could generally be classified as belonging in one of four broad and somewhat overlapping categories: acquisition, process, analysis, and presentation. The design of models organizing these extraneous metadata attributes tends to be scientific domain specific, although some commonalities exist. The CSD model allows the inclusion of metadata models describing these other categories using generic application objects. An application can place its own attribute type, e.g., a dictionary object with application-specific metadata attributes inside each generic application object using a reverse domain name notation string as the attribute key, for example, “com.example.myApp”. The use of a reverse-DNS key provides a simple mechanism for reducing name-space collisions. Overall, we believe generic application objects give the CSD model enough flexibility to become the native file format of many applications.

This approach, however, creates a dilemma when CSDM files are saved and opened by different applications. Specifically, what does an application from company B (e.g., “com.B.process”) do with generic application objects placed in a CSDM file by an application from company A (e.g., “com.A.acquire”)? On the one hand, company B could retain the company A specific metadata (as found) in the generic application object using the “com.A.acquire” key as well as serialize its own metadata using the “com.B.process” key. If the company B application made any modification to the dataset, however, it runs the risk that parts of the company A application-specific metadata are now irrelevant or logically inconsistent with the newly saved dataset—potentially causing company A’s application to crash when it tries to open this newly saved dataset. On the other hand, company B could decide to discard the company A specific metadata, in which case the company A application can safely open the dataset saved by company B, but will have lost all of its previously saved metadata. Finding a consistent solution to this dilemma is critically important as one can easily envision a workflow where a dataset passes through many applications as it progresses from the raw dataset to the final “product.” During such a workflow there is often an expectation of an audit trail, which most likely could be determined from application metadata saved by each application used during the workflow.

One approach that could solve this dilemma is to allow the CSDM file to contain a time-ordered array of CSDM objects. In other words, company B would simply append a second CSDM object with only company B metadata to the array that already contains the CSDM object created by company A. No application metadata would be lost, and the metadata in each CSDM object would be relevant and logically consistent with its respective datasets. In this approach, the CSDM “array” file would grow as each application completes its task in the overall workflow.

It is our opinion, however, that it is better to delegate such a task of managing a time-ordered array of CSDM objects to the operating system. In this approach, we envision the workflow associated with a particular dataset to result in a folder containing a series of CSDM files, each a snapshot from the workflow as it progresses from the raw dataset to the final “product.” When each application is finished with its workflow task a CSDM file is saved with the read_only flag set to true, so that any future work on the dataset would be performed on a copy of the CSDM dataset, leaving the “read-only” file with application metadata intact. Typically, the read_only flag would be set to true immediately after the acquisition of raw data, after processing is complete, or after analysis of a dataset. Delegating the task of managing a time-ordered set of CSDM objects to the operating system also makes the workflow status involving individual CSDM files more transparent to the end-user. In adopting this solution we propose the general rule that *while* *application* *attributes should be visible to any application opening a CSDM file, only the reverse-DNS owners have permission to use their respective keys to place an attribute in an* *application object*.

An application could implement an additional layer of protection from application metadata loss by saving CSDM compliant files with its own application-specific file extension. Other applications could still open the CSDM compliant file but would be discouraged from saving with another application’s file extension.

### 2.6 JSON file-serialization

A JSON file is ordinarily a UTF-8 encoded text file which is built on two structures: a collection of unordered key-value pairs and an ordered list of values. The “key”: value pair is separated by a *colon* symbol, with the key to the left and the value to the right of the colon. Different key-value pairs are separated using *commas*. The JSON keys are always wrapped in double quotation marks, as in “key”, and the value type can either be (a) a string, (b) a number, (c) a JSON object, (d) an array, (e) a boolean or (d) null. A string is a composition of JSON allowed characters [1] wrapped in double quotation marks. A number can be integer or float. A JSON object is an unordered set of key-value pairs which begins with a left curly brace, {, and ends in a right curly brace, }. An array is an ordered collection of JSON values that begins with a left square bracket, [, and ends in a right square bracket, ]. A boolean is true or false. In the JSON serialization of the CSD model, the JSON “ key “ corresponds to the attribute name of the various CSD model objects while the JSON value and CSD model attribute value follow the relationship listed in Table 6.

Efforts have been made in the design of the CSD model to keep the keys intuitive and self-explanatory to all scientists and engineers. To further enhance the human-readability aspect of the files, we recommend, as a general rule, that no key be present in the file unless its value differs from the default value. With this in mind, the CSD model defines all boolean values as false when unspecified. In other words, the only boolean keys that need to appear in the file are those set to true.

The serialization file names are designated with two possible extensions: .csdf and .csdfe, the acronyms for Core Scientific Dataset Format and Core Scientific Dataset Format External. When all data values are stored within the file, i.e., there are no instances of an **ExternalDependentVariable** object in the serialization, then the .csdf file extension is allowed, otherwise, the serialization file name must use the extension .csdfe. This difference in extensions is intended to alert the end-user to a possible risk of failure if the external data file is inaccessible when deserializing a file with the .csdfe file extensions.

## 3 *d*D{*p*_0_, *p*_1_, …} example datasets

In this section we examine the CSD model in a number of illustrative examples. We use a shorthand notation of *d*D{*p*} to indicate that a dataset has a *p*-component dependent variable defined on a *d*-dimensional coordinate grid. In the case of correlated datasets the number of components in each dependent variable is given as a list within the curly braces, i.e., *d*D{*p*_0_, *p*_1_, *p*_2_, …}.

Efforts have been made to include examples across disciplines, although given our expertise in magnetic resonance spectroscopy, we include multiple examples from this field. It is worth noting, however, that magnetic resonance datasets prove to be excellent test cases for the CSD model as they are diverse and often multi-dimensional in nature. We have converted a variety of datasets from various fields to the CSD model format. To accomplish this, we utilize several Python packages [5, 6] to import the original field-specific scientific datasets as Numpy [7] array(s) and export the latter in the CSD model format using the csdmpy package for Python, described in the appendix.

### 3.1 1D{1} examples

In this section, we examine the JSON serialization for illustrative cases of 1D{1} datasets. These are the simplest cases, with one dimension, *d* = 1, and one single-component dependent variable, *p* = 1. The supplementary material gives further 1D{1} examples from FITR, UV-vis, and EPR spectroscopies.

#### GMSL.csdf

An example of a JSON serialized CSD model holding a 1D{1} dataset is shown in Listing 1. This dataset is a measurement of the global mean sea level [8] (GMSL) based on the satellite altimeter data from 1993-2009.

**Listing 1. CSD model depiction of the global mean sea level dataset.** A JSON serialized CSD model describing the global mean sea level dataset. The listing was created by the authors using data from reference [8].

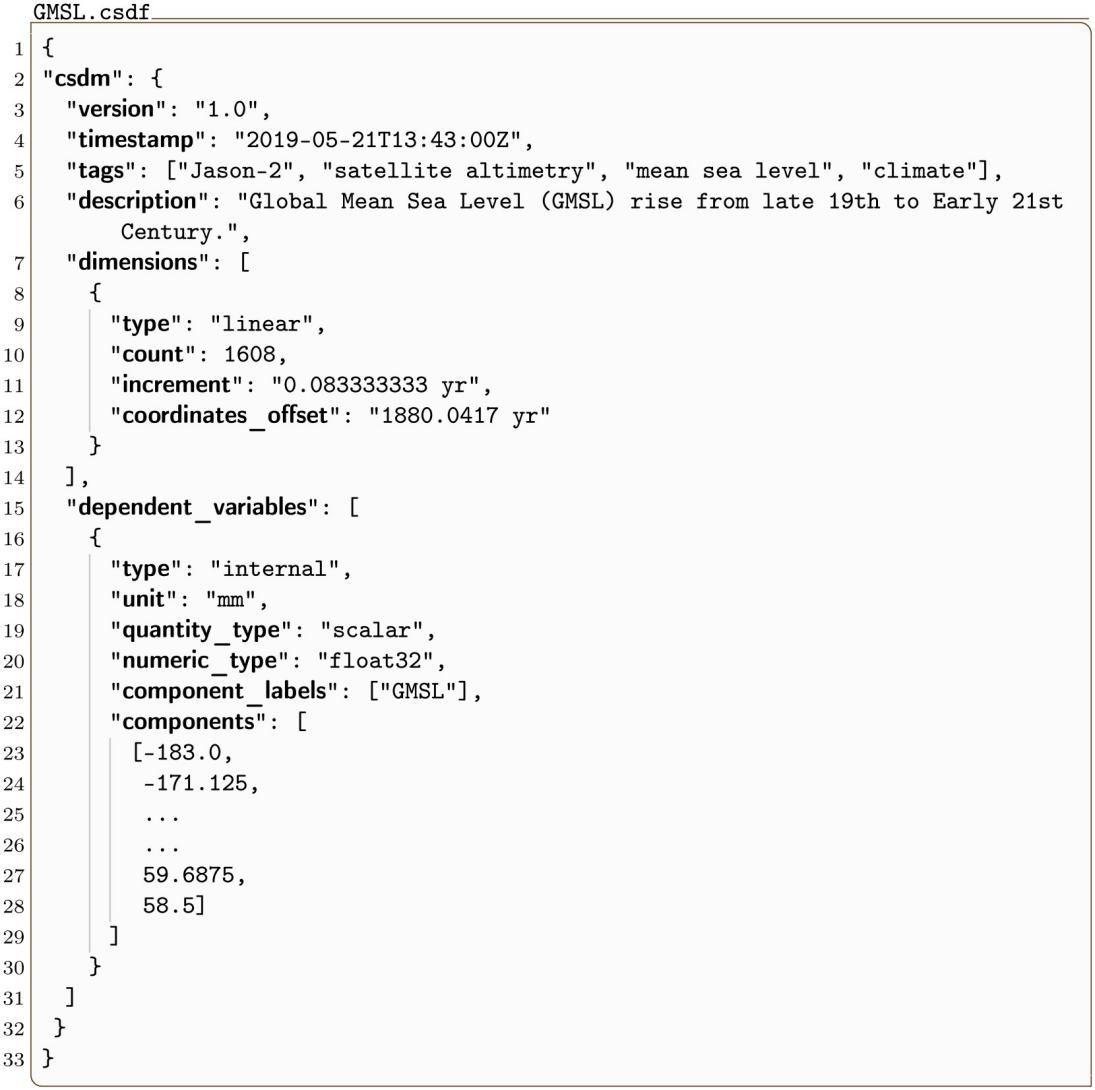


At the root level is the csdm key, an acronym for the core scientific dataset model. The value of this key is a JSON object which is a serialization of the CSD model’s **CSDM** object and includes six keys—version, timestamp, tags, description, dimensions, and dependent_variables. The value of the dimensions key is an array (lines 7-14) with a single JSON object defined in-between lines 8 and 13. This object is a JSON serialization of the CSD model’s **Dimension** object. In this example, it represents a **LinearDimension** object, as indicated by the value of linear in the type key, and with a coordinate count of 1608 as defined by the value of the key count. Furthermore, it is a temporal dimension with **ScalarQuantity** values of 0.08333 yr for increment and 1880.0417 yr for coordinates_offset. The coordinates at vertexes along this temporal dimension are obtained from Eq (3).

The value of the dependent_variables key is an array (lines 15-31) with a single JSON object describing the global mean sea level. This object is a JSON serialization of an **InternalDependentVariable** object, with data values stored within the object, as indicated by the value of internal for the type key. The data values are serialized as JSON numbers as seen in-between lines 23-28 of Listing 1. Ellipses indicate where superfluous lines were omitted from the listing. The value of float32 for the numeric_type key indicates that the array of JSON numbers should be converted into a numerical array of data values with 32-bit floating-point precision on import. The value of mm for the unit key is the unit associated with the data values. The value of the component_labels is an array with a single entry holding the label associated with the component values. The value of scalar for the quantity_type key indicates that the component of the dependent variable is interpreted as scalar.

**Fig 2:**
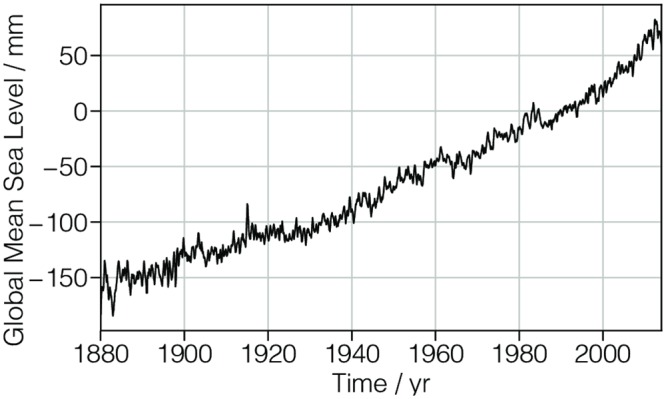
CSD model depiction of the global mean sea level dataset. A line plot, derived from Listing 1, depicting the global mean sea level as a function of time. The figure was created by the authors using data from reference [8].

A plot of the dataset is shown in Fig 2. Note that meta-data on how a dataset is presented in a plot or otherwise is not included in the CSD model. While such presentation metadata is outside the scope of the core model, it can be included in an application dictionary.

#### blochDecay.csdf

Another simple example of a 1D{1} dataset, acquired by the authors for this work, is shown in Listing 2. This example corresponds to a ^13^C free induction decay signal from a nuclear magnetic resonance spectroscopy of ethanol.

**Listing 2**. **CSD model depicting an 1-D NMR dataset**. JSON serialized CSD model describing the ^13^C NMR Bloch decay time signal along with the relevant metadata of the reciprocal frequency dimension.

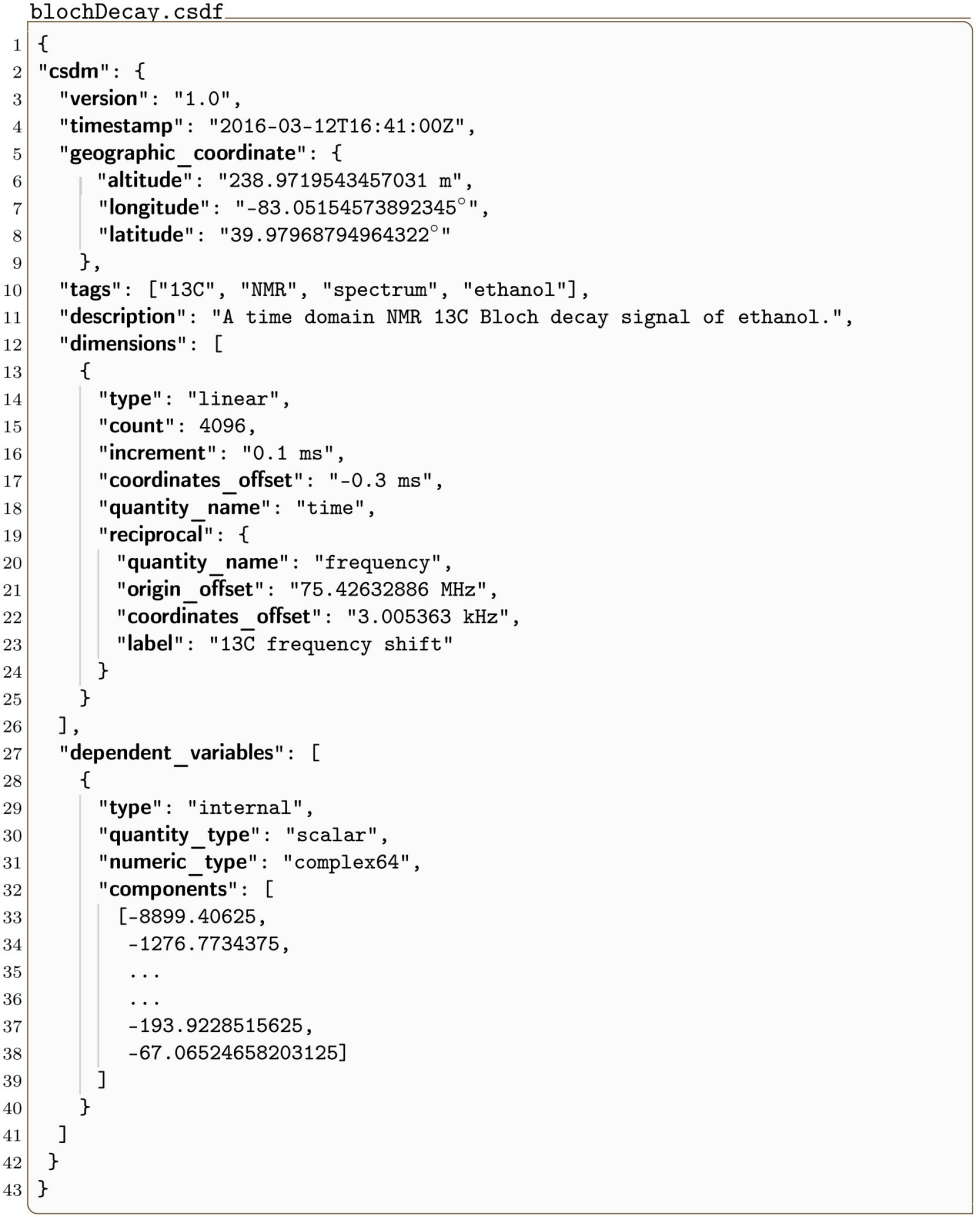


The value of the dimensions key is an array (lines 12-26) with a single JSON serialized **LinearDimension** object (lines 13-25) representing a temporal dimension with 4096 coordinate positions sampled every 0.1 ms starting at −0.3 ms. The coordinate values along the dimension are evaluated using Eq (3). This **LinearDimension** object also contains an optional JSON serialized **ReciprocalDimension** object (lines 19-24) as the value of the reciprocal key. In this example, it provides the metadata needed for describing the reciprocal time or the frequency dimension, i.e., after a Fourier transform.

**Fig 3:**
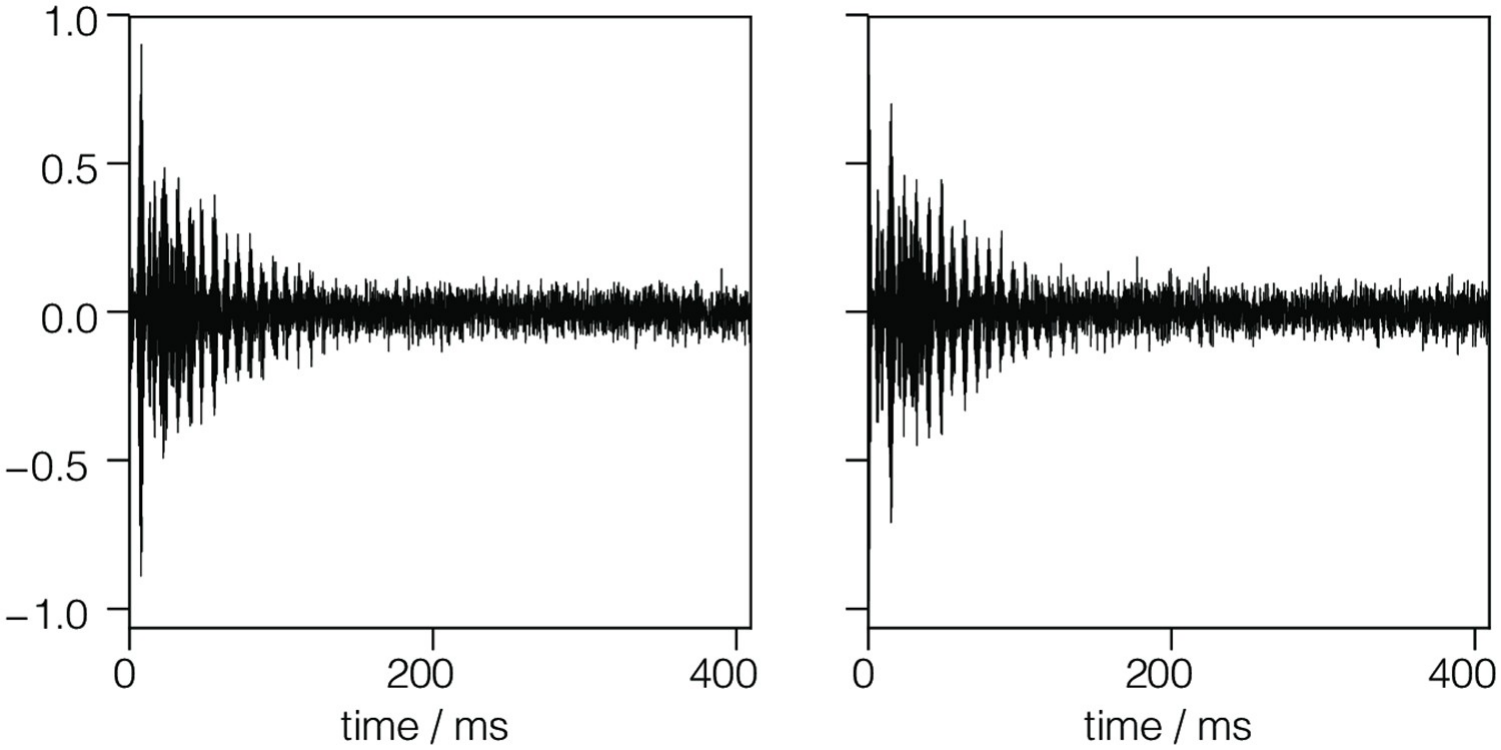
CSD model depicting an 1-D NMR dataset. A plot, derived from Listing 2, of the real (left) and imaginary (right) ^13^C NMR Bloch decay signal as a function of time.

The value of the dependent_variables key is an array (lines 27-41) with a single JSON serialized **InternalDependentVariable** object (lines 28-40) describing the signal response. While the keys and values in this object are similar to the corresponding object from the previous example, a key difference is that the value of the numeric_type key denotes a complex64 numeric type. Complex numbers are stored as an ordered array of alternating real and imaginary data values, starting with the real value. In this example, the first and the last complex numbers of the signal in Fig 3 are (−8899.406 − *i*1276.773) and (−193.923 − *i*67.065), respectively. Note that the length of the ordered data array is 2*M* for complex numeric types, where *M* is the total number of sampled data points. Fig 3 shows a line plot of the time domain NMR decay signal.

#### acetone.csdf

In Listing 3 is an illustration of a 1D{1} mass spectrum dataset [9] serialized with sparse sampling. Here the **InternalDependentVariable** object (lines 17-43) holds a **SparseSampling** object (lines 23-34) in the sparse_sampling key. Inside the **SparseSampling** object are the three keys dimensions_indexes, sparse_grid_vertexes, and unsigned_integer_type. The dimensions_indexes key holds an array of integers specifying the indexes of the dimensions along which the dependent variable is sparsely sampled. In this case, it is the zeroth dimension, i.e., the only dimension in the dataset. The sparse_grid_vertexes key holds an array of integers specifying the vertexes on the one-dimensional sparsely sampled grid. Again, in this example with only one dimension, the array of integers corresponds to the sampled sparse grid vertexes, i.e., the coordinate indexes, *j*_0_, along the zeroth dimension. The value uint8 for the unsigned_integer_type key is the numeric type used when importing the JSON serialized integer array from the sparse_grid_vertexes key.

**Listing 3. CSD model depiction of a sparse mass spectrum.** JSON serialized CSD model describing the mass spectrum of acetone.

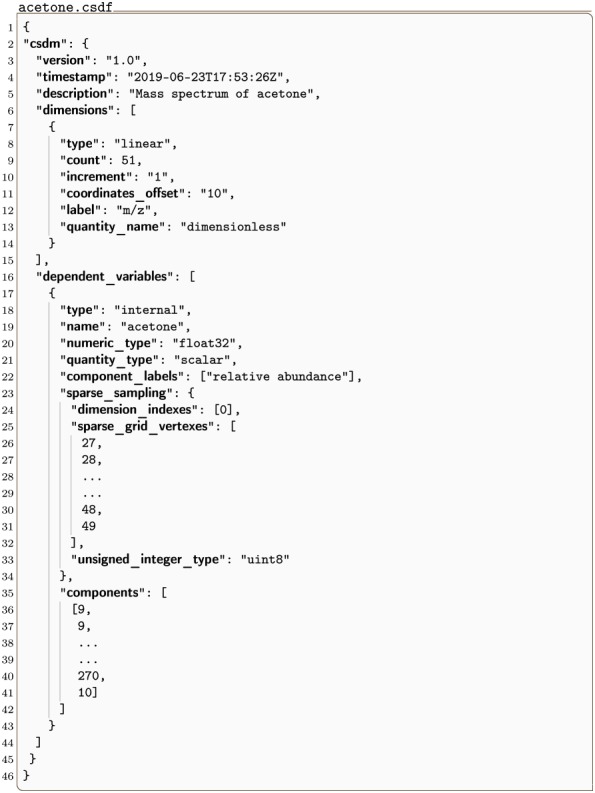


### 3.2 2D{1} examples

#### TEM.csdf

**Fig 4:**
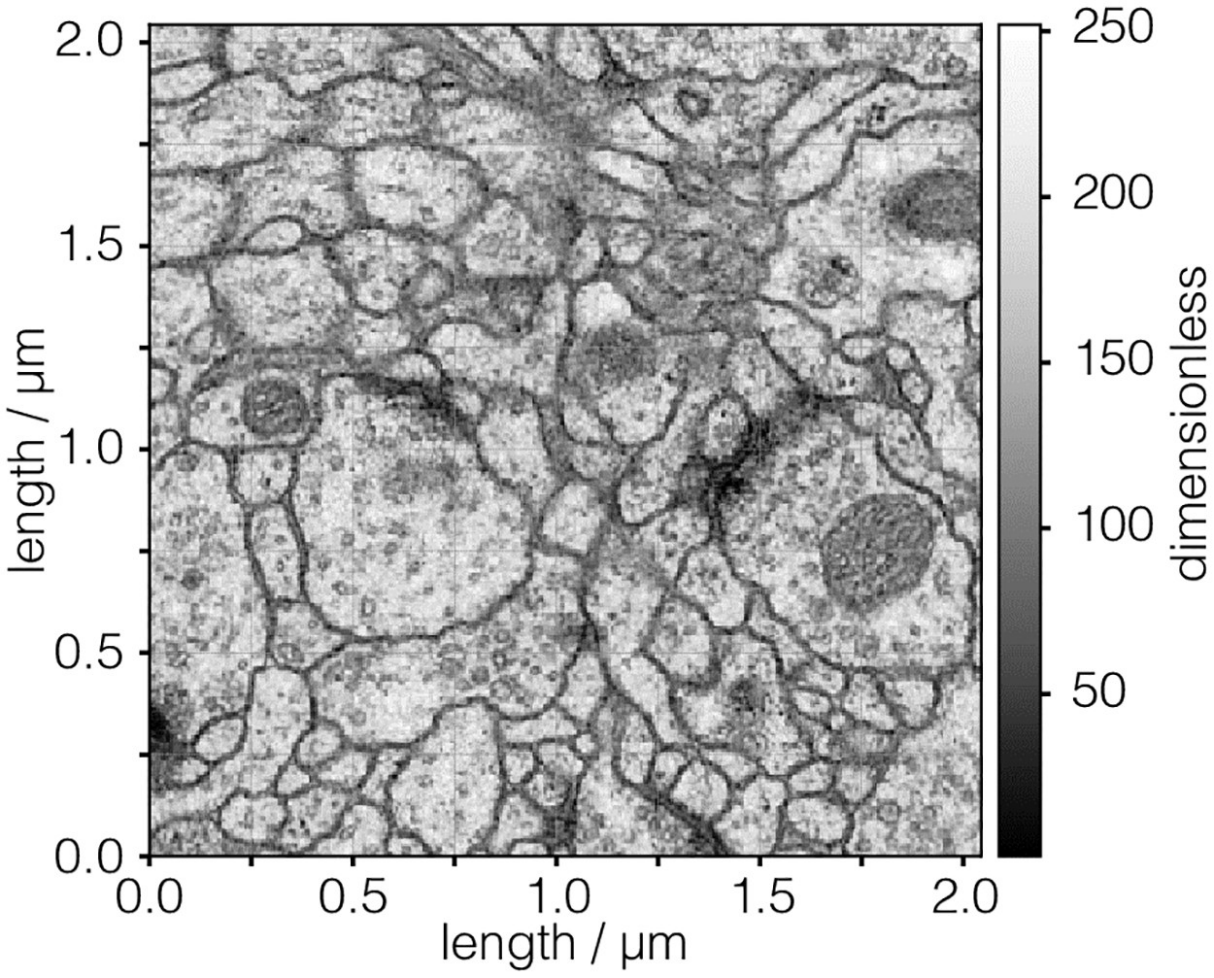
CSD model depiction of a TEM image dataset. An intensity plot, derived from Listing 4, of a TEM dataset depicting the early larval brain of *Drosophila melanogaster*. The figure was created by the authors using data from reference [10].

In Fig 4 is an intensity plot of a Transmission Electron Microscopy (TEM) dataset of a section of the early larval brain of *Drosophila melanogaster* used in the analysis of neuronal microcircuitry [10]. The CSDM JSON serialization for this 2D{1} dataset is given in Listing 4. This dataset has two dimensions, *d* = 2, and one single-component dependent variable, *p* = 1.

**Listing 4. CSD model depiction of a TEM image dataset.** JSON serialized listing of a TEM dataset containing one single-component **InternalDependentVariable** object and two **LinearDimension** objects. The listing was created by the authors using data from reference [10].

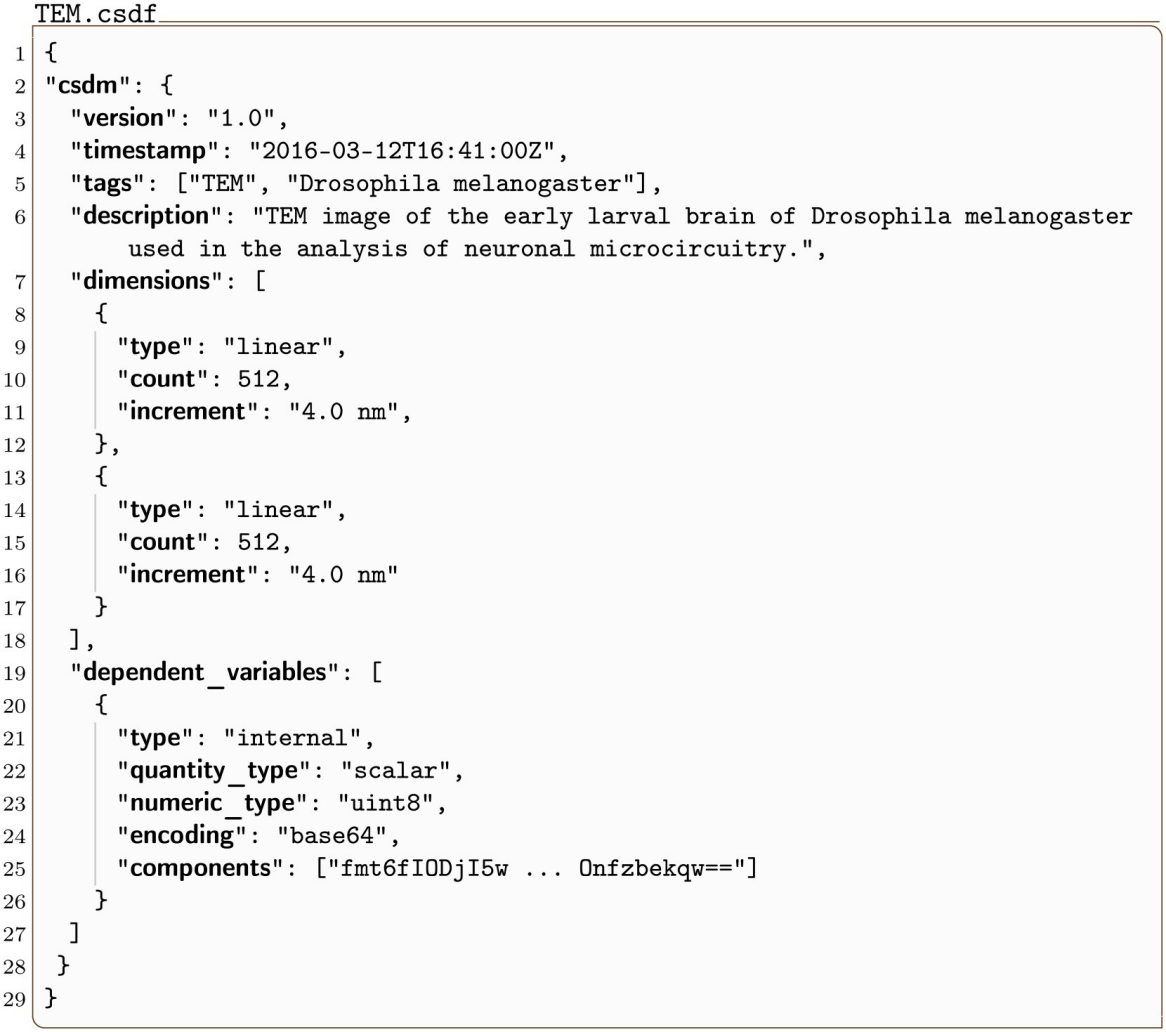


The value of the dimensions key is an array with two JSON serialized **LinearDimension** objects, defined in-between lines 8-12 and 13-17. Both these objects describe a linearly sampled spatial dimension with 512 points sampled every 4 nm. As before, Eq (3) gives the ordered list of the coordinates along the respective dimensions. The value of the dependent_variables key is an array containing a single JSON serialized **InternalDependentVariable** object (lines 20-26). Unlike the previous examples, the value of the components key is an array with a single element. This element is a Base64 encoded string, as indicated by the encoding key, and decodes to an array of binary data values which are interpreted as an array of numerical values with a uint8 numeric type. The array of numerical values is then mapped to the 512 × 512 coordinate grid according to Eqs (7) and (8).

#### bubble.csdfe

**Fig 5:**
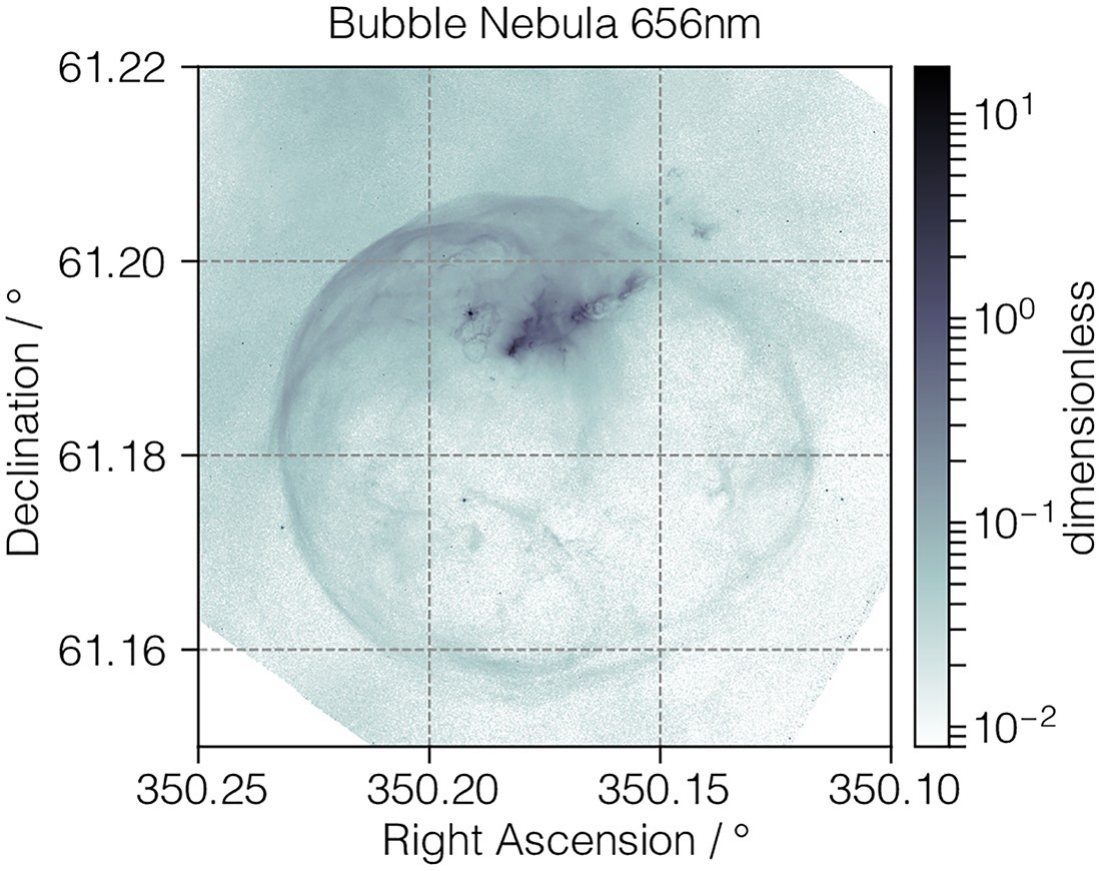
CSD model depiction of an astronomy image dataset. A log intensity plot, derived from Listing 5, of the bubble nebula [11] observed at 656 nm wavelength. The figure was created by the authors using data from reference [11].

In Fig 5 and in Listing 5 we present a 2D{1} astronomy dataset of the bubble nebula acquired at 656 nm wavelength by the Hubble Heritage Project [11] team. In this example, the value of the dimensions key is an array with two JSON serialized **LinearDimension** objects defined in lines 8-15 and 16-23. Both these objects describe a linearly sampled angular dimension. The value of the dependent_variables key is an array with a single JSON serialized **ExternalDependentVariable** object, described in lines 26-32. In this example, the value of the type key is external, indicating that the data values are stored in an external file located at the Uniform Resource Locator (URL) address given by the components_url key. In this case, the address corresponds to a local file, designated by the file scheme of the URL, relative to the location of the bubble.csdfe file. The external file holds an ordered array of 11592 × 11351 binary values, which are specified by the numeric_type key as 32-bit floating-point numbers.

**Listing 5. CSD model depiction of an astronomy image dataset.** JSON serialized listing of the astronomy dataset describing the bubble nebula observed at 656 nm wavelength. The listing was created by the authors using data from reference [11].

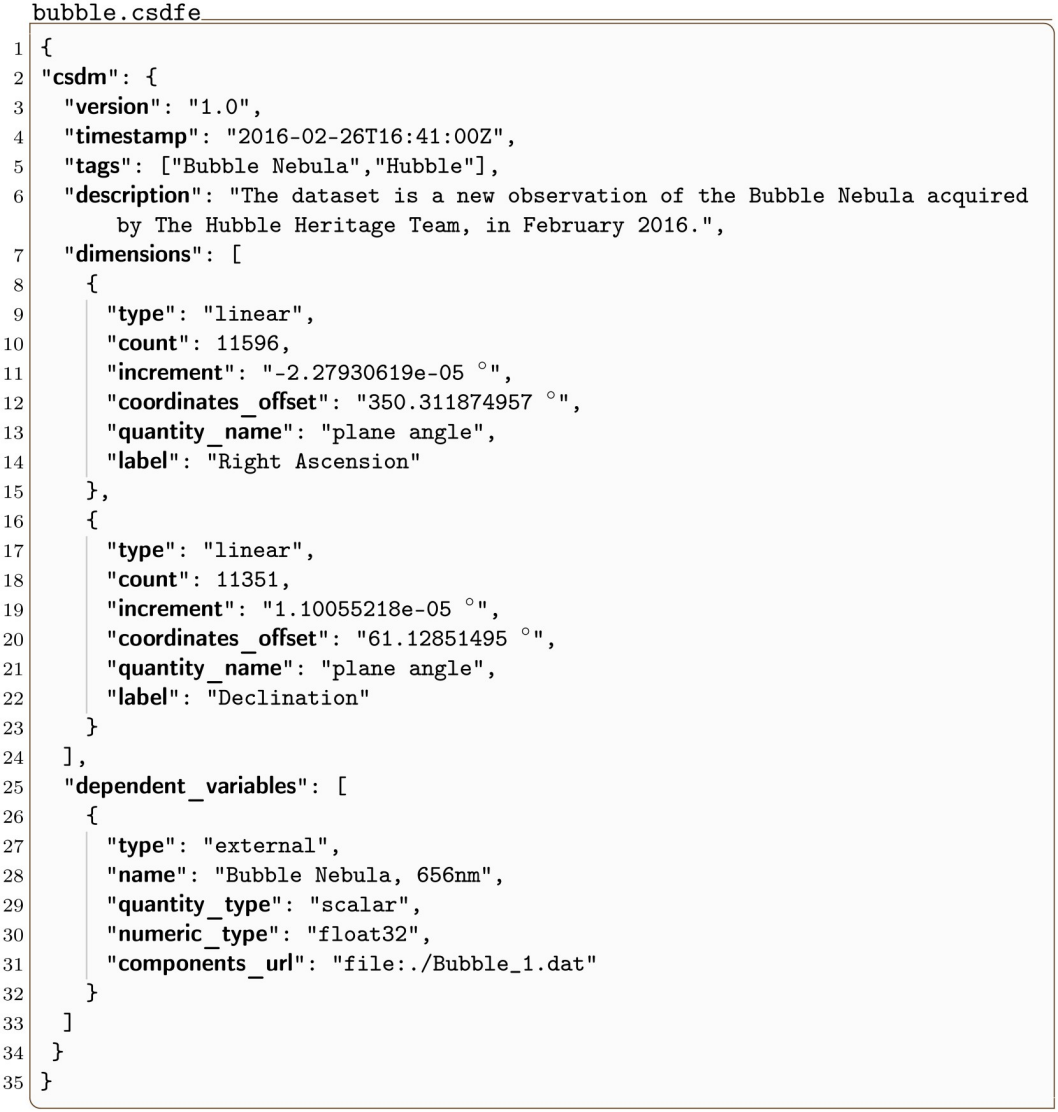


#### satRec.csdf

**Fig 6:**
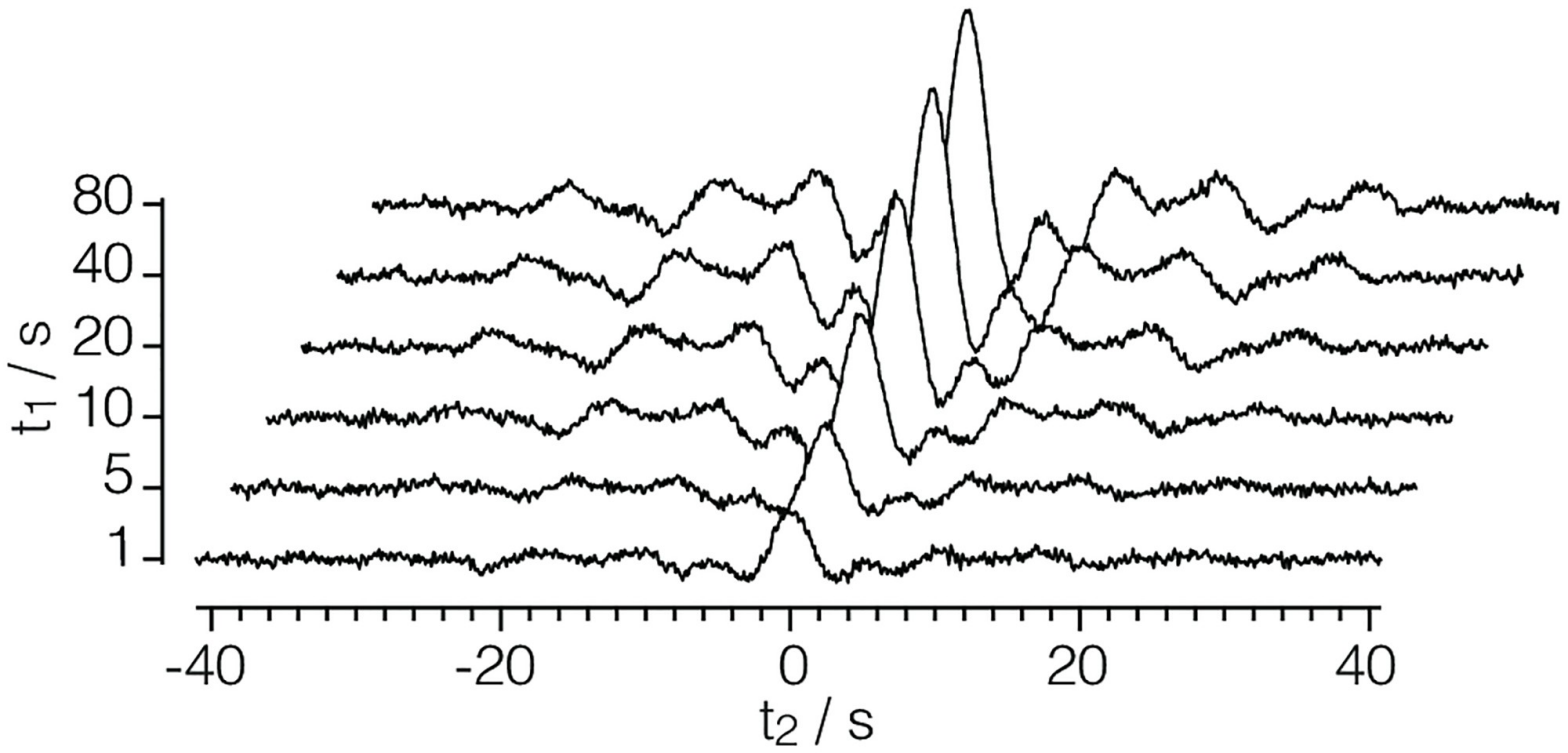
CSD model depiction of a 2-D NMR dataset. A stacked plot, derived from Listing 6, of an NMR dataset depicting the ^29^Si saturation recovery measurement of a highly siliceous ZSM-12 zeolite.

A monotonic dimension is employed when measurements are not uniformly spaced or span several orders of magnitude along a dimension. An example of a 2D{1} dataset with a monotonic dimension, acquired by the authors, is given in Listing 6. Here the dataset comes from a ^29^Si NMR magnetization recovery measurement of a highly siliceous ZSM-12 zeolite sampled on a 2D rectilinear grid. Fig 6 depicts a stacked plot corresponding to the dataset from Listing 6.

**Listing 6. CSD model depiction of a 2-D NMR dataset.** JSON serialized listing of ^29^Si NMR magnetization saturation relaxation dataset containing one single-component **DependentVariable** object and two **Dimension** objects.

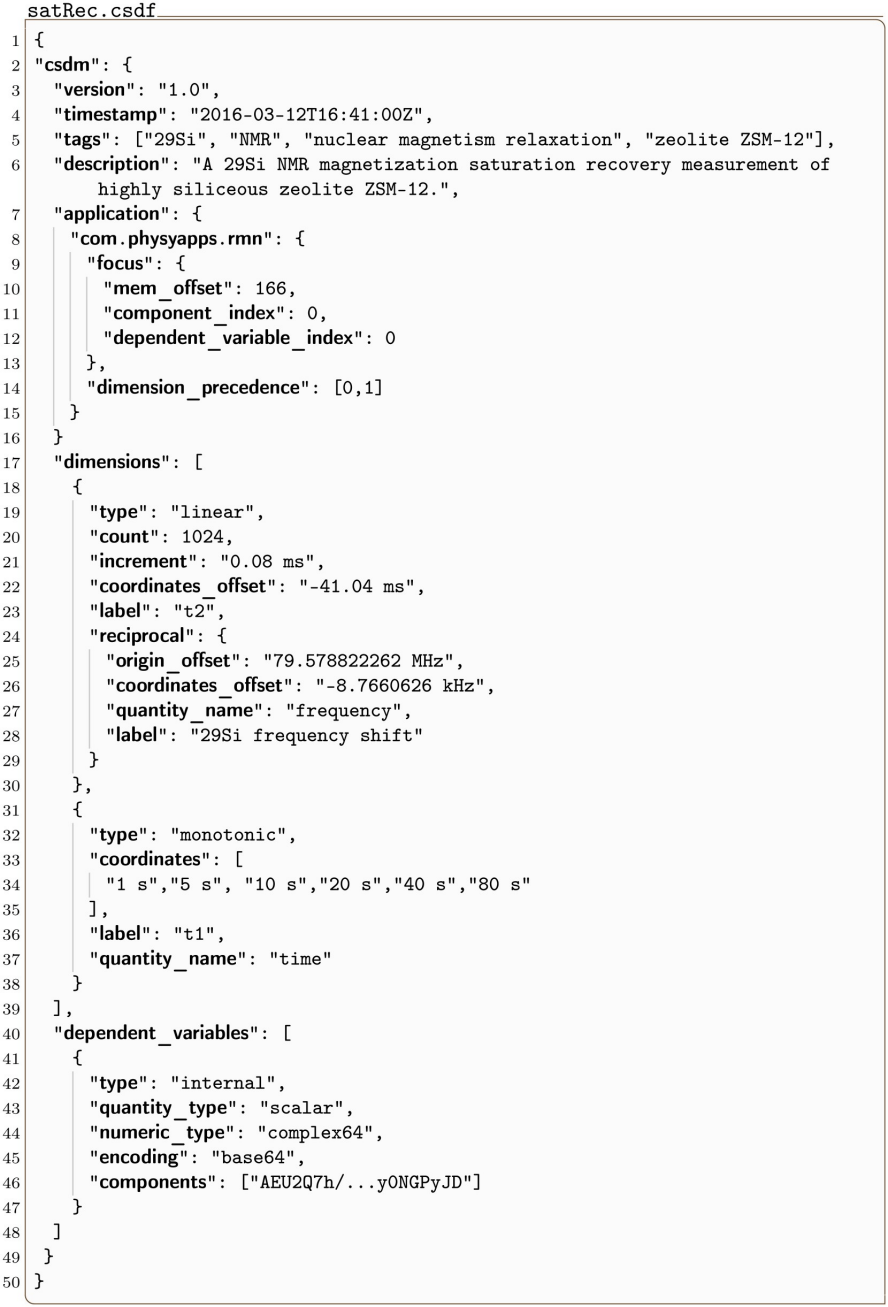


The value of the dimensions key is an array with two JSON serialized **Dimension** objects (lines 18-30 and 31-38). The first is a **LinearDimension** object, labeled as *t*_2_, describing a temporal dimension with 1024 points sampled at every 80 *μ*s with a coordinates_offset of −41.04 ms. Additionally, this **LinearDimension** object contains a **ReciprocalDimension** object serialized as the value of the reciprocal key. The second is a **MonotonicDimension** object, labeled as *t*_1_, with the coordinates associated with grid vertexes along the dimension explicitly given in the ordered set of values in the coordinates key. The value of the dependent_variables key is an array with a single JSON serialized **InternalDependentVariable** object (lines 41-47) describing the signal response. Here, the data values are encoded as an array with one Base64 string in the components key.

This listing also gives an example of the use of the application key in the csdm dictionary. Here an application owning the domain name physyapps.com has placed an attribute in the application dictionary using the reverse domain name key com.physyapps.rmn. Domain name owners are free to place any valid JSON object as the value of their respective reverse domain name attribute inside the application dictionary. In this case, the domain name owner has used the reverse domain name key com.physyapps.rmn to place a dictionary holding two keys, focus and dimension_precedence.

An application key can also be placed in any **Dimension**, **ReciprocalDimension**, **DependentVariable**, and **SparseSampling** object. Again, according to the rule in section 2.5, only the reverse domain name owner has permission to serialize a file using their respective reverse domain name as a key in the application attribute.

#### iglu_1d.csdf

**Fig 7:**
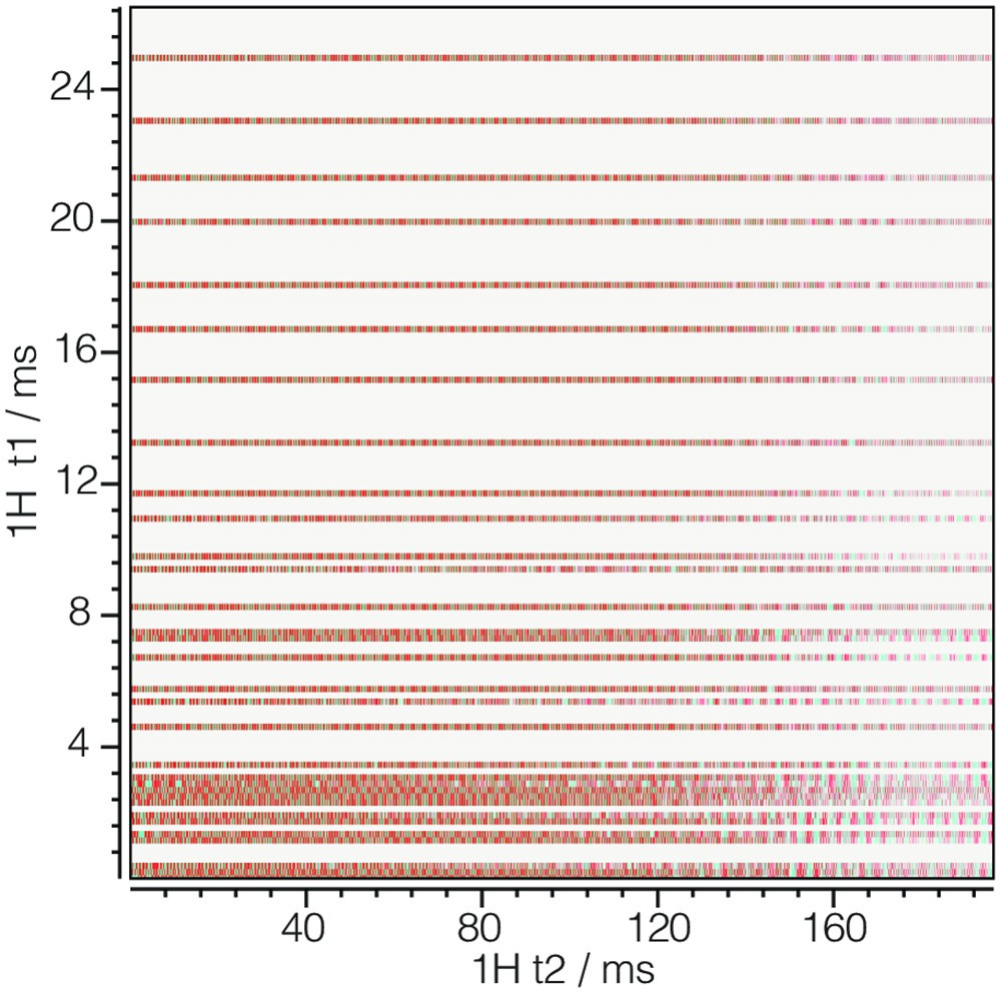
CSD model depiction of a sparse NMR dataset with one sparse dimension. A plot, derived from Listing 7, of the real part of a 2D{1} dataset sparsely sampled in one dimension. The figure was created by the authors using data from reference [12].

Listing 7 is a 2D{1} example of an NMR signal shown in Fig 7 with sparse sampling along one dimension [12]. Here the **InternalDependentVariable** object (lines 30-44) holds a **SparseSampling** object (lines 37-43) in the sparse_sampling key. The **SparseSampling** object contains three keys dimension_indexes, sparse_grid_vertexes, and unsigned_integer_type. The dimension_indexes key holds an array of integers specifying the indexes of the dimensions along which the dependent variable is sparsely sampled, in this case, the *k* = 1 dimension. The sparse_grid_vertexes key holds an array of integers specifying the vertexes on the one-dimensional sparsely sampled grid. Since there are two dimensions in the dataset the array of integers corresponds to the coordinate indexes, *j*_1_, along the *k* = 1 dimension. In this example, the dependent variable values are fully sampled along the *k* = 0 dimension. The value of the unsigned_integer_type key holds the numeric type used in importing the integer array from sparse_grid_vertexes.

**Listing 7. CSD model depiction of a sparse NMR dataset with one sparse dimension.** JSON serialized listing of ^13^C-^15^N NMR HSQC dataset containing one single-component **DependentVariable** object and two **Dimension** objects. The listing was created by the authors using data from reference [12].

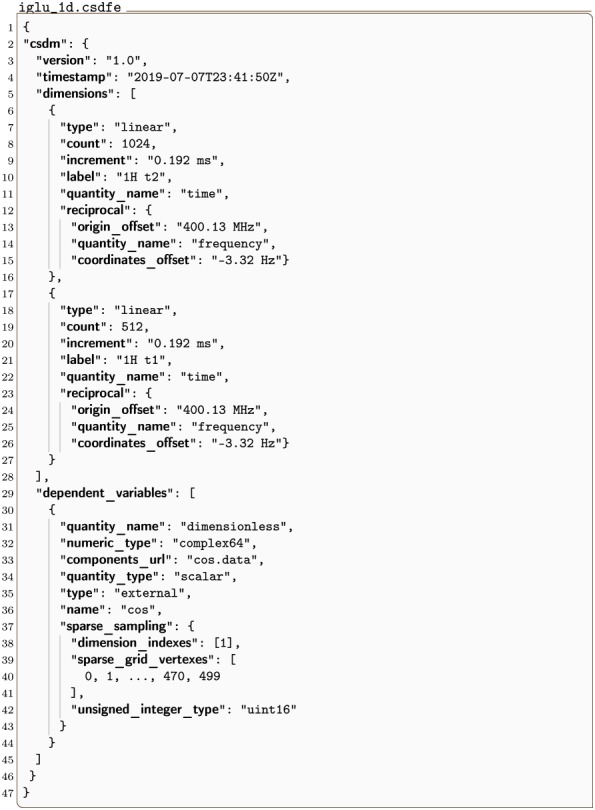


#### iglu_2d.csdf

**Fig 8:**
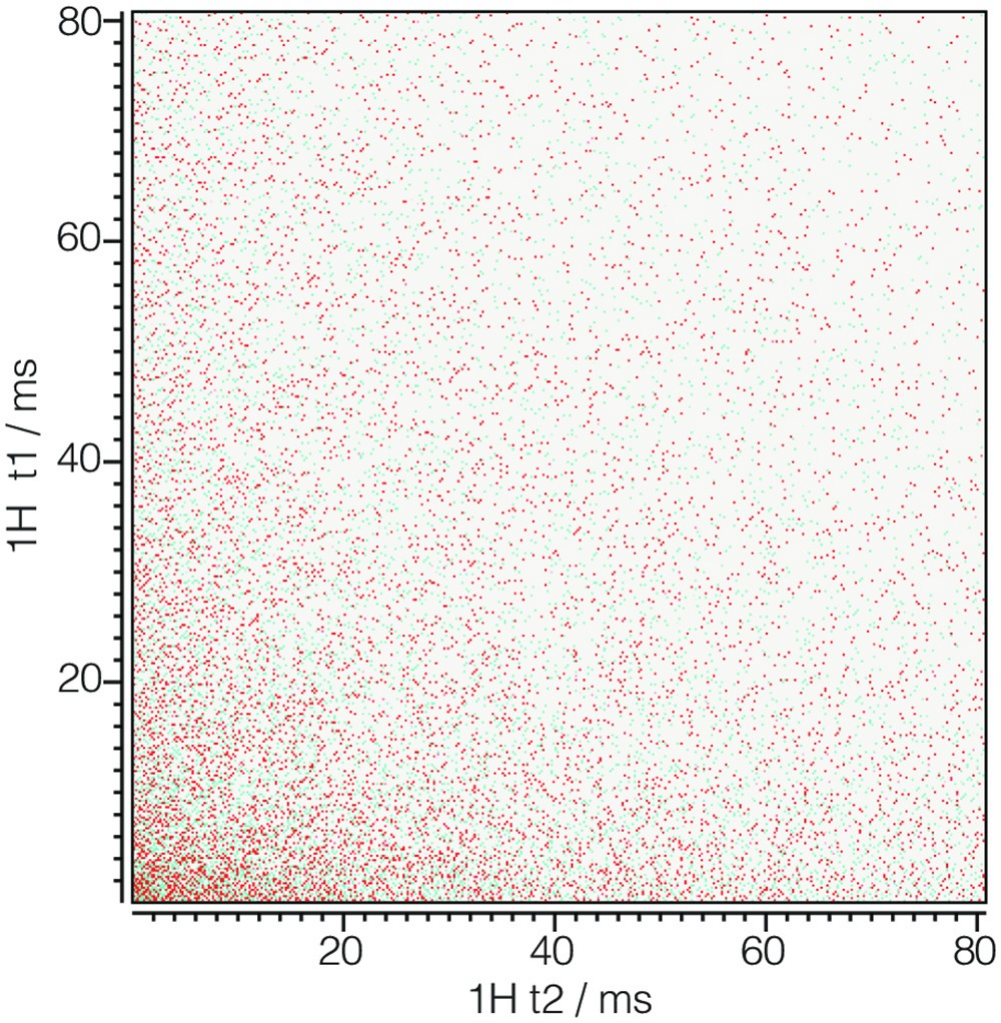
CSD model depiction of a sparse NMR dataset with two sparse dimensions. A plot, derived from in Listing 8, of the real part of a 2D{1} dataset sparsely sampled in both dimensions. The figure was created by the authors using data from reference [12].

Listing 8 is a 2D{1} example of an NMR signal shown in Fig 8 with sparse sampling along two dimensions [12]. As before, the sparse_sampling key holds a **SparseSampling** object with the dimension_indexes, sparse_grid_vertexes, and unsigned_integer_type attributes. The dimension_indexes key holds an array of two integers, *k* = 0 and 1, specifying the sparse sampling dimensions. The sparse_grid_vertexes key holds an array of integers defining the vertexes on the two-dimensional sparsely sampled grid. As described in section 2.4.1 this array is a flattened ordered set of arrays which can be reshaped into the ordered set of sparse grid vertexes, i.e.,

$$\begin{array}{c} \left[0,0,1,0,\ldots ,972,511,1015,511\right]\;\;⟶\;\;[[0,0],[1,0],\ldots ,[972,511],[1015,511]]. \end{array}$$

**Listing 8. CSD model depiction of a sparse NMR dataset with two sparse dimensions.** JSON serialized listing of ^1^H NMR TOCSY dataset containing two **Dimension** objects and one single-component **DependentVariable** object with sparsely sampled values in both dimensions. The listing was created by the authors using data from reference [12].

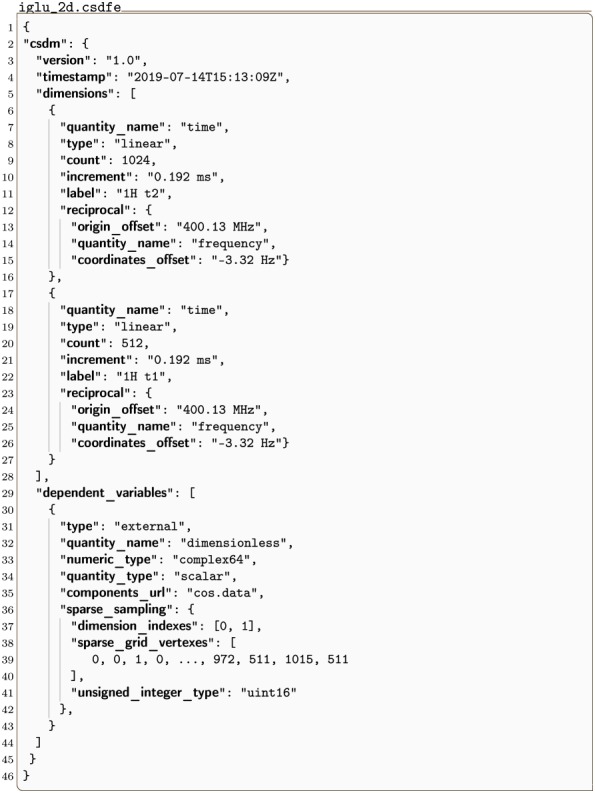


The *i*^th^ vertex in the ordered set of sparse grid vertexes specifies the sparse grid location of the *i*^th^ value in each component array of the dependent variable.

### 3.3 2D{3} example

#### RGB_image.csdf

**Fig 9:**
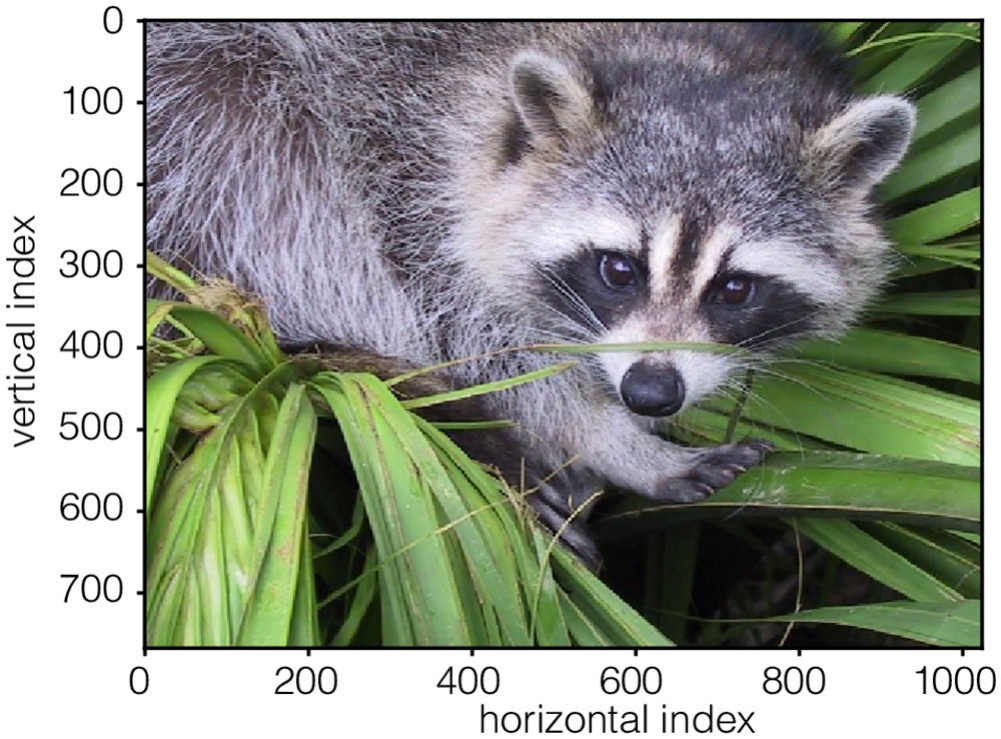
CSD model depiction of a RGB image dataset. An image plot, derived from Listing 9, of an RGB dataset depicting a raccoon face. Photo credit: Judy Weggelaar.

A simple example of a 2D dataset with multiple components is a color image [13], such as the one shown in Fig 9. This is a 2D{3} dataset, with two **LinearDimension** objects and one three-component dependent variable, *p* = 3. The CSDM serialization is shown in Listing 9. The dimensions key holds an array with two JSON serialized **LinearDimension** objects (lines 8-13 and 14-19) with 1024 and 768 points, respectively, and a unit sampling interval. The dependent_variable key holds an array with a single JSON serialized **InternalDependentVariable** object (lines 22-34) containing an image dataset as indicated by the pixel_3 value of the quantity_type key. The first part, pixel, indicates pixel data, and the last part, 3, gives the number of pixel components. An array holding to the three components, i.e., the red, green, and blue color intensities with each encoded as a Base64 string, is the value of the components key. The Base64 decoded binary data values are then interpreted as an array of 8-bit unsigned integer (uint8), for each component, and subsequently mapped onto a 1024 × 768 coordinate grid. The value of the component_labels key is an array of the labels ordered to match the order of the components.

**Listing 9. CSD model depiction of a RGB image dataset.** JSON serialized listing of an RGB image dataset containing two **Dimension** objects, and one **DependentVariable** object with three components corresponding to red, green and blue color intensities. The listing was created by the authors using the data [13] available under (Creative common 0) CC0 license.

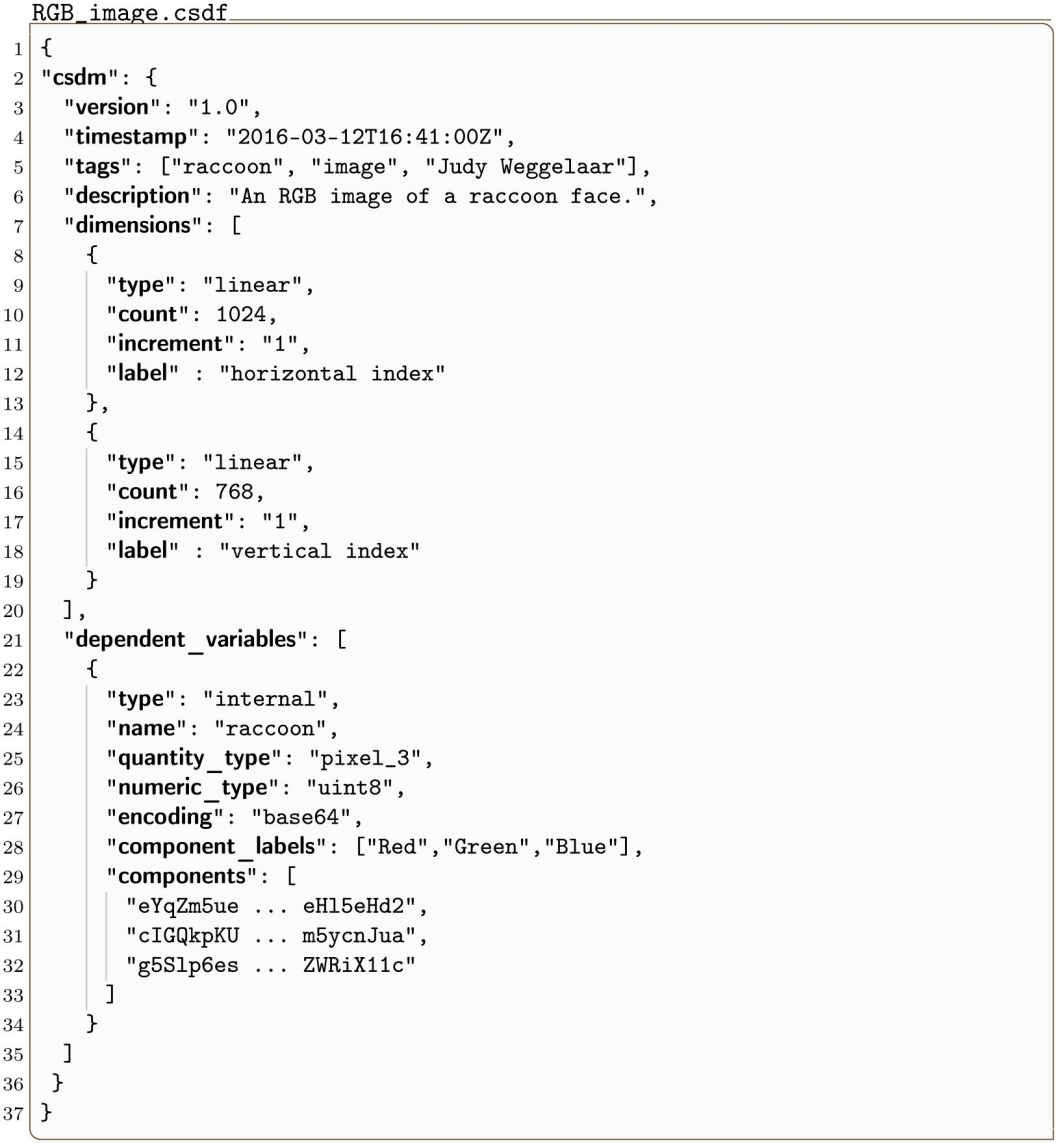


### 3.4 3D{2} example

#### wind_velocity.csdfe

An example of a 3D{2} dataset, i.e., with three dimensions, *d* = 3, and one two-component dependent variable, *p* = 2, is the wind velocity prediction [14] dataset as a function of latitude, longitude and time, shown in Listing 10.

**Listing 10. CSD model depiction of a meteorology vector dataset.** JSON serialized listing of the predicted wind velocities over and around the Gulf of Mexico. The model contains one two-component **DependentVariable** object and three **Dimension** objects. Listing was created by the authors using data from the national centers for environment information/national oceanic and atmospheric administration [14].

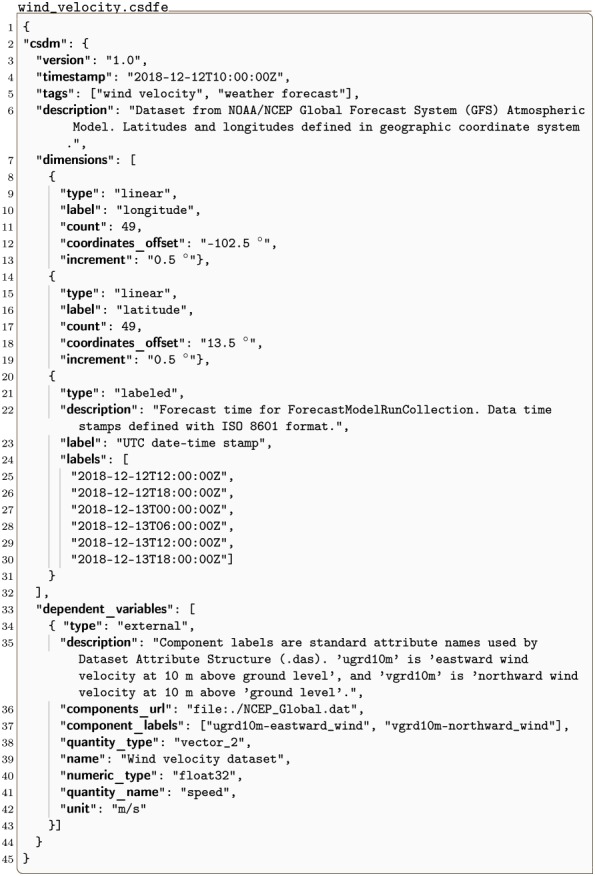


**Fig 10:**
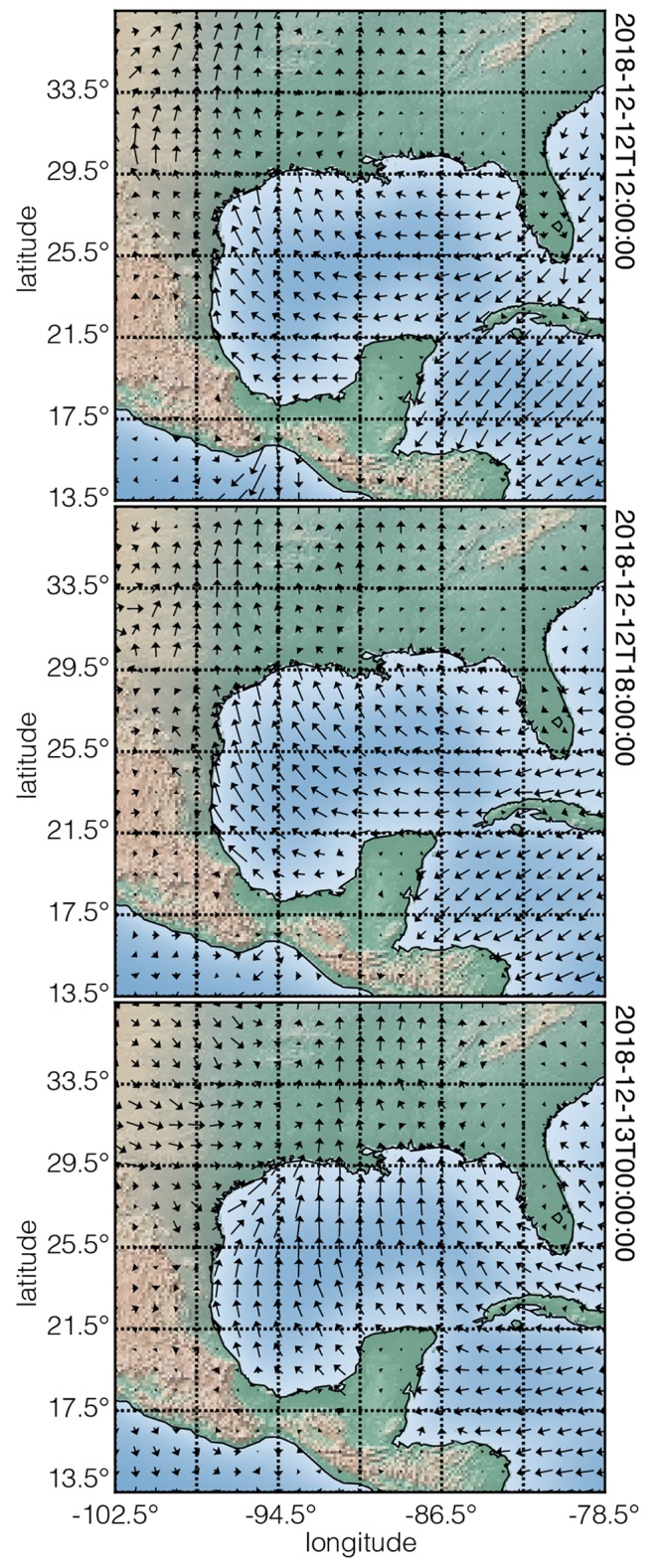
CSD model depiction of a meteorology vector dataset. A quiver plot, derived from Listing 10, of the wind velocities from the dataset in Listing 10 at three different date-time stamps. The underlaid map of the Earth corresponding to the latitudes and longitudes is rendered using the Matplotlib Basemap toolkit [15]. The figure was created by the authors using data from reference [14].

The value of the dimensions key is an array with three JSON serialized **Dimension** objects. The first two **LinearDimension** objects, labeled as *longitude* and *latitudes* respectively, describe two linear dimensions sampled at every 0.5° for 49 points starting at −102.5° longitudes and 13.5° latitudes. Together, these two objects create a two-dimensional grid that spans the region around the Gulf of Mexico as depicted in Fig 10. The third dimension is a **LabeledDimension** object as indicated by the value of the type key. The corresponding labels array lists six *date-time stamps* entries.

The value of the dependent_variable key is an array with a single JSON serialized **ExternalDependentVariable** object (lines 34-43) containing a two-component vector dataset as identified by the quantity_type key-value. This value is vector_2 where the first part, vector, indicates vector data, and the last part, 2, gives the number of vector components. The two vector components are labeled as ugrd10m-eastward_wind and vgrd10m-northward_wind, in the array assigned to the component_labels key. The data values are located in an external file as a binary data whose address, relative to the wind_velocity.csdfe file, is the value of the components_url key. The binary data is interpreted as a 32-bit floating-point numerical array. Note, because the binary data does not support array indexing, unlike JSON serialization, the corresponding numerical array of data values is reshaped into a matrix which includes the number of components. In this case, the reshaped matrix is 49 × 49 × 6 × 2, where the last number is the number of components, *p* = 2, and the remaining three is the number of points from the **Dimension** objects. Table 3 contains a description of the number of components, *p*, for each quantity_type.

Fig 10 depicts a quiver plot of the wind velocity at three different date-time stamps. Underlaid these plots is a map of the Earth corresponding to the given range of latitudes and longitudes. These plots were generated using the Matplotlib library [16] for python in addition to the Matplotlib Basemap toolkit [15] for rendering maps.

### 3.5 3D{6} example

#### brain_MRI.csdf

A 3D{6} dataset has three dimensions, *d* = 3, and one six-component dependent variable, *p* = 6. An example of such a dataset is the second rank symmetric diffusion tensor MRI dataset [17] of a brain given in Listing 11.

**Listing 11. CSD model depiction of an MRI tensor dataset.** JSON serialized listing of the diffusion tensor MRI dataset [17] of the brain containing one six-component **DependentVariable** object and three **Dimension** objects. Listing was created by the authors using data from reference [17].

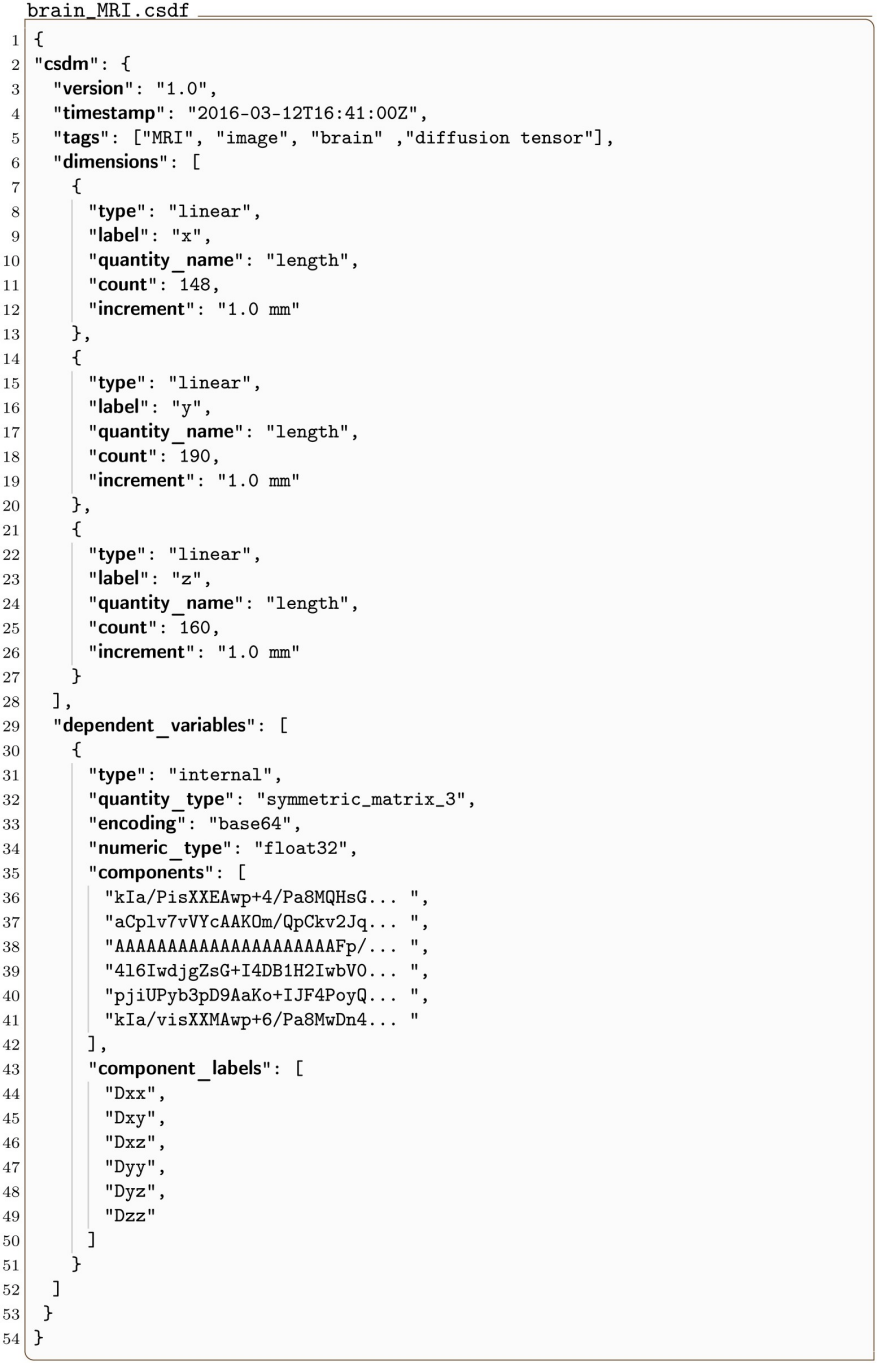


The value of the dimensions key is an array with three JSON serialized **Dimension** objects describing the three spatial dimensions, labeled as *x*, *y*, and *z* respectively. Here, all objects describe a linear dimension with the sampling resolution of 1 mm, and 148, 190 and 160 points along the respective dimension.

The value of the dependent_variables key is an array with a single JSON serialized **InternalDependentVariable** object (lines 30-51) describing a symmetric matrix dataset as indicated by the value of the quantity_type key. The value symmetric_matrix_3 emphasizes a six-component dataset as noted in Table 3. The six components, labeled as *Dxx*, *Dxy*, *Dxz*, *Dyy*, *Dyz*, and *Dzz* respectively, are stored as Base64 strings as the value of the components key. Each Base64 decoded binary array is interpreted as 32-bit floating-points array and subsequently reshaped to a 148 × 190 × 160 matrix.

**Fig 11:**
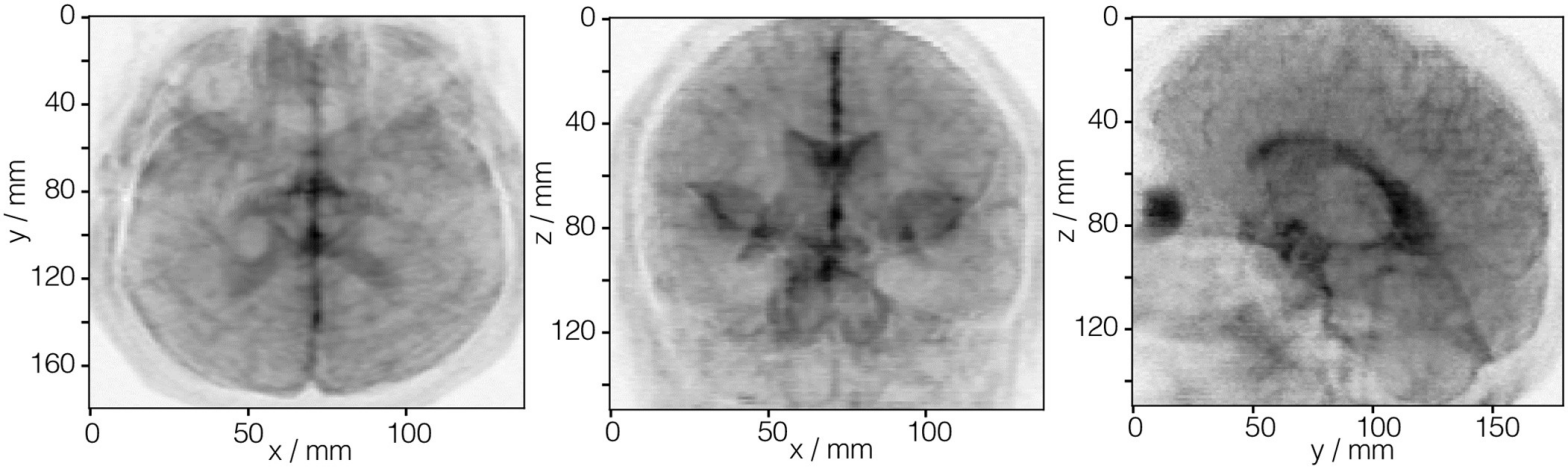
CSD model depiction of an MRI tensor dataset. The intensity plots, derived from the diffusion tensor MRI dataset in Listing 11, are the projection of the isotropic diffusion coefficients, calculated on to the three spatial dimensions. The figure was created by the authors using data from reference [17]. The diffusion tensor MRI Brain dataset [17] courtesy of Gordon Kindlmann at the Scientific Computing and Imaging Institute, University of Utah, and Andrew Alexander, W. M. Keck Laboratory for Functional Brain Imaging and Behavior, University of Wisconsin-Madison.

The symmetric matrix data from the brain_MRI.csdf file was partially processed as a second-rank symmetric diffusion tensor to determine the isotropic diffusion coefficients. The intensity plots in Fig 11 depicts the projection of the isotropic diffusion coefficients on to the three spatial dimensions.

### 3.6 2D{1,1,2,1,1} example

An example of a 2D{1,1,2,1,1} dataset using data from the US National Centers for Environment Information / National Oceanic and Atmospheric Administration [14] is given in Listing 12.

**Listing 12. CSD model depiction of a meteorology dataset with multiple dependent-variables.** JSON serialized listing of multiple dependent variables including scalar and vector on a two-dimensional grid. Listing was created by the authors using data from the US National Centers for Environment Information / National Oceanic and Atmospheric Administration [14].

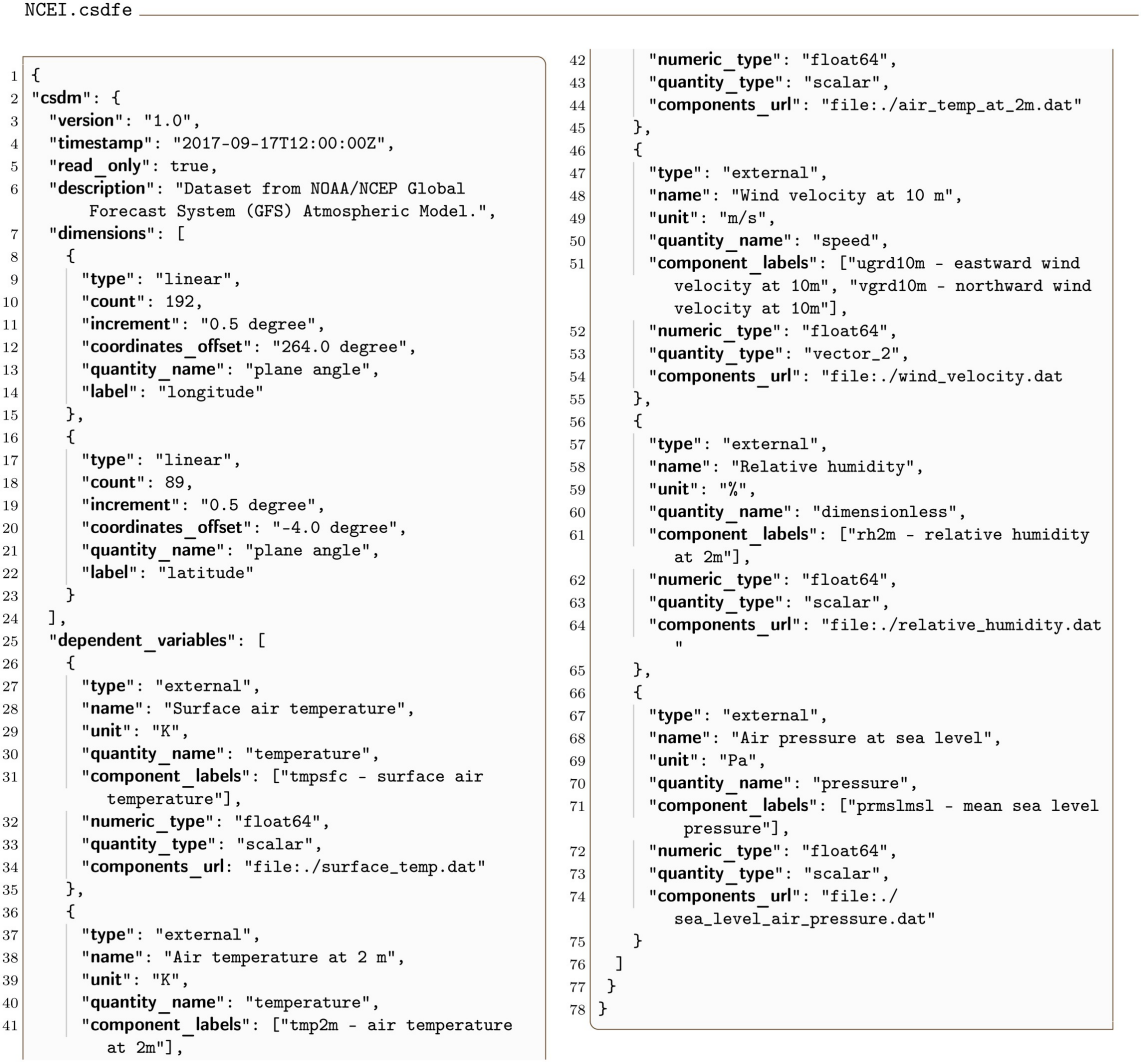


**Fig 12:**
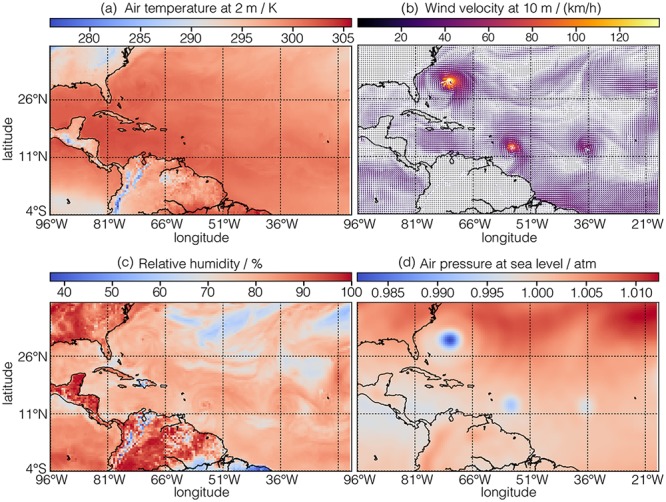
CSD model depiction of a meteorology dataset with multiple dependent-variables. The figure depicts (a) an intensity plot of the air temperature at 2 m above surface, (b) a quiver plot of the wind vectors at 10 m above surface, (c) an intensity plot of the relative humidity, and (d) an intensity of the air pressure at sea level corresponding to the last four dependent-variables from Listing 12. These plots are overlaid on the coastline map of the Earth corresponding to the latitude and longitudes. These coastline were rendered using the Matplotlib Basemap toolkit [15]. The plots were generated using the Matplotlib library [16] for python. The figure was created by the authors using data from reference [14].

In this example, the value of the dimensions key is an array with two **LinearDimension** objects describing the two spatial dimensions, labeled as *longitude* and *latitude*, respectively. The value of the dependent_variables key is an array with five **ExternalDependentVariable** objects describing the surface temperature (*p*_0_ = 1), the air temperature at 2 m above ground level (*p*_1_ = 1), the two-component wind velocity vector at 10 m above surface (*p*_2_ = 2), the relative humidity (*p*_3_ = 1), and the air pressure at the sea level (*p*_4_ = 1). Fig 12 depicts the intensity and quiver plots of four dependent variables.

### 3.7 0D{1,1} example

#### J_vs_s.csdf

**Fig 13:**
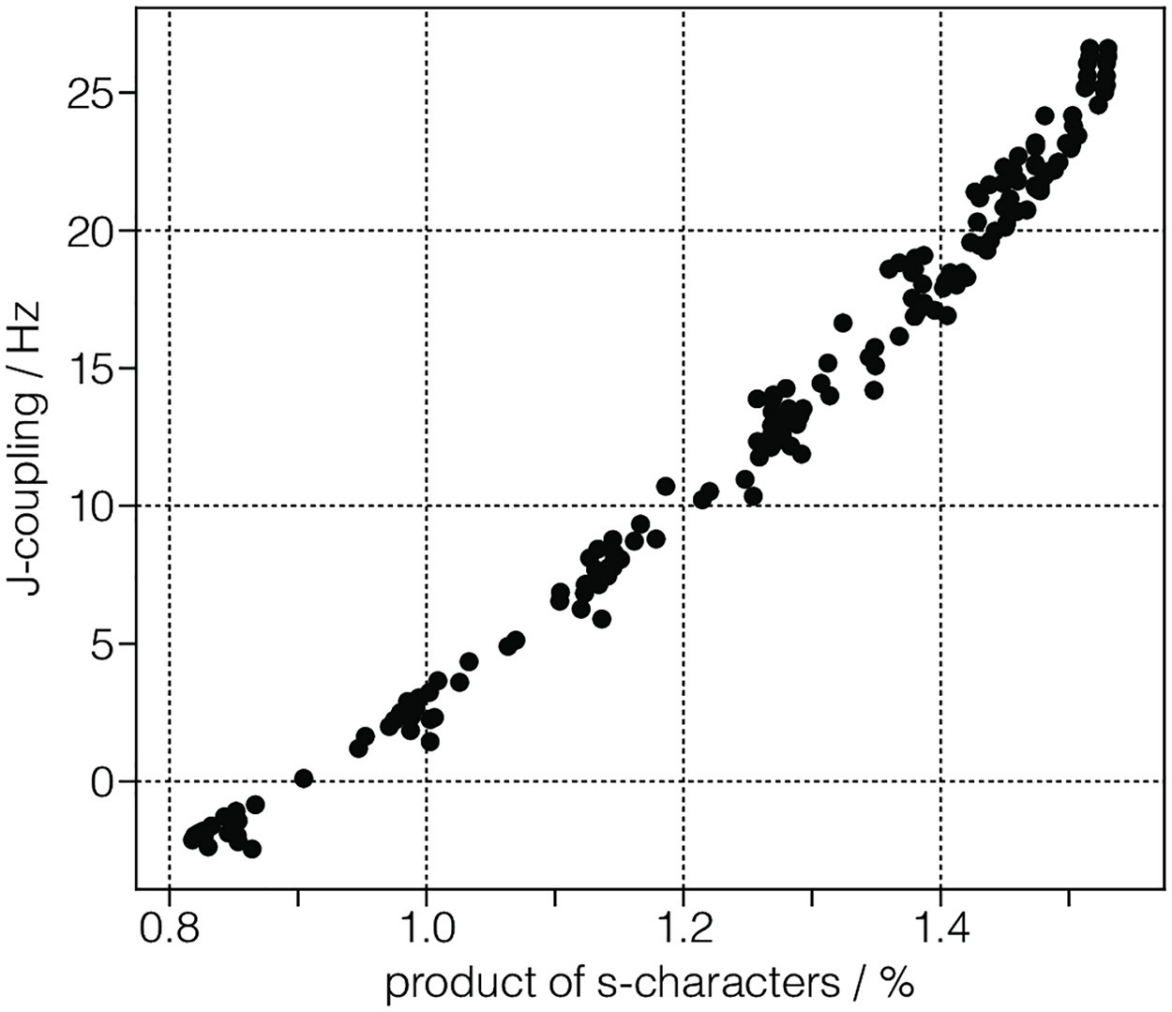
CSD model depiction of a computational dataset. Two dependent variables [18] correlating ${}^{2}J_{\text{Si-O-Si}}$ couplings to the corresponding product of *s*-characters on the Si, O and Si atoms along the Si-O bond across the Si-O-Si linkage. The figure was created by the authors using data from reference [18].

The CSD model also allows the serialization of datasets without a coordinate grid. A 0D{1,1} datasets, for example, has no dimensions, *d* = 0, and two single-component dependent variable, *p*_0_ = 1 and *p*_1_ = 1. The listing for such a dataset [18] is given in Listing 13. In this example, the two “correlated” dependent variables are the ^29^Si-^29^Si nuclear spin couplings, ^2^*J*, across a Si-O-Si linkage and the *s*-character product on the O and two Si along the Si-O bond across the Si-O-Si linkage [18]. The value of the dependent_variables key is an array with two JSON serialized **InternalDependentVariable** object (lines 7-16 and 17-27). The first object, named as *Gaussian computed J-couplings*, describes the ^2^*J* couplings. The data values are stored as a Base64 string in the components key. The Base64 decoded binary array is interpreted as a 32-bit floating-point numerical array following the value of the numeric_type key. The second object is named as the *product of s-characters*. Here, the data values are again stored as a Base64 string, which after decoding is interpreted as a 32-bit floating-point numerical array. A scatter plot revealing the correlation between the two dependent variables from the dataset in Listing 13 is presented in Fig 13.

**Listing 13. CSD model depiction of a computational dataset.** JSON serialized listing of quantum chemistry calculation of nuclear spin-spin coupling constant between ^29^Si nuclei across a Si-O-Si linkage in small cluster molecule. An example dataset with two **DependentVariable** objects and no **Dimension** objects. The listing was created by the authors using data from reference [18].

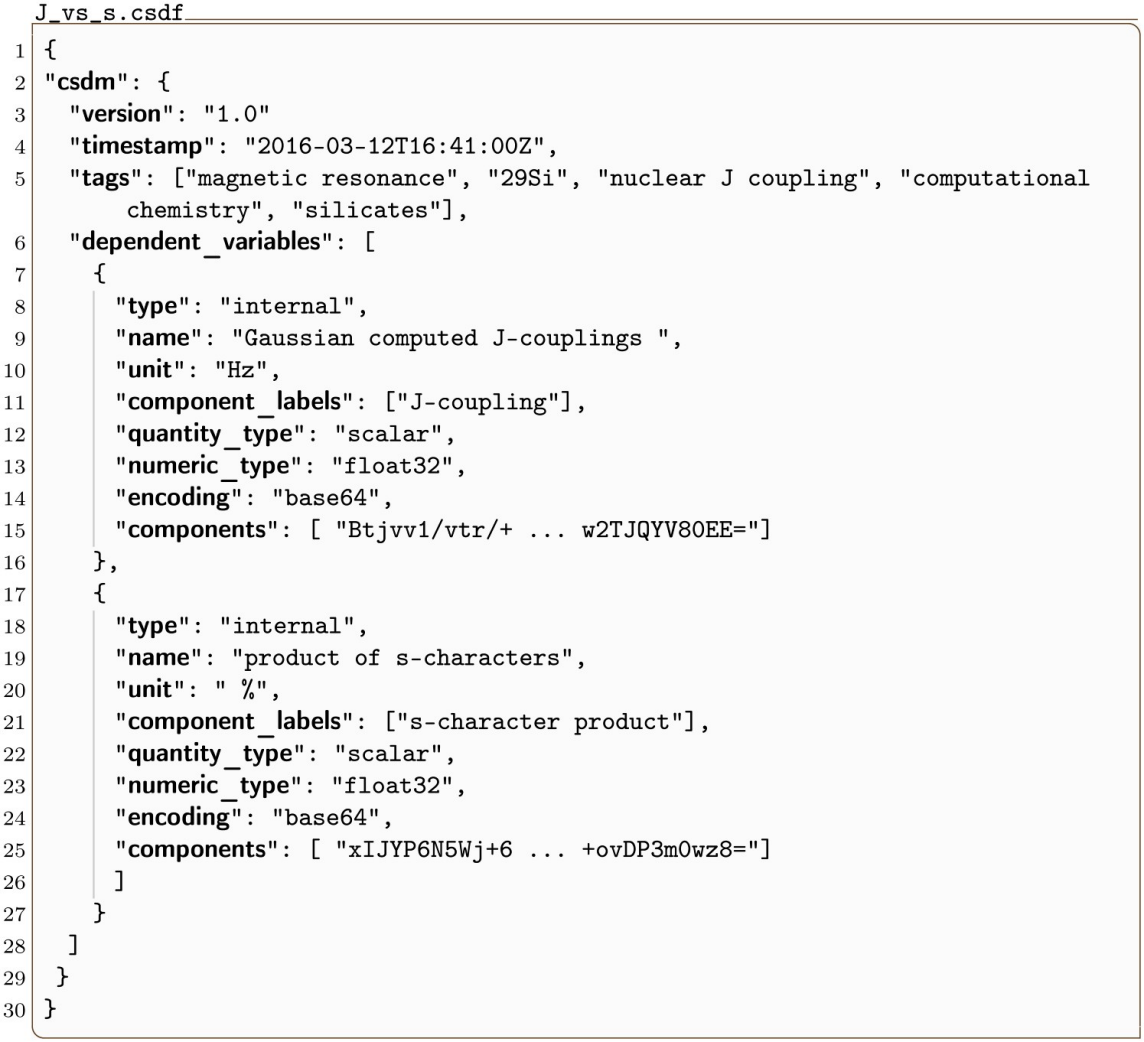


## 4 Conclusions

We have designed the Core Scientific Dataset (CSD) Model as a lightweight, portable, versatile, resourceful, and standalone data model that is capable of handling multi-dimensional and correlated datasets from various spectroscopies, diffraction, microscopy, and imaging techniques. A guiding principle in the design of this model was to encapsulate only the minimal metadata necessary to represent the correlated datasets sampled on a common orthogonal coordinate grid. The model also allows for sparse sampling on this grid. Throughout the model, we make use of the **ScalarQuantity** class, which is composed of a numerical value and any valid SI unit symbol or any number of accepted non-SI unit symbols. This approach enables tremendous flexibility in allowing the dataset model to be agnostic of the scientific domain. Historically, this may have been perceived as a potential barrier to software implementation of the CSDM, however, in recent years libraries capable of parsing units have become freely available for various computing environments such as Matlab, Mathematica, and python. The CSD model is independent of the hardware, operating system, application software, and file-serialization method used for data exchange. The model provides a mechanism for the inclusion of additional application-specific metadata without compromising its fundamental role as a data exchange and archiving standard. When serialized using JSON serialization the resulting file format is human readable and integrable with most object-oriented programming languages and software. The serialization of the CSD model has been adopted as an open dataset file format in NMR software development under our control, i.e., SIMPSON [19, 20], DMFIT [21], jsNMR [22], and RMN [23], which already have a large installed user base within the solid-state NMR scientific community. We envision the CSD model and its associated file format as playing an important role in community accessible databases and in greater data-trail integrity and compliance issues for many research laboratories.

## Appendix

### Scaled variables

Coordinates along a dimension can also be converted into scaled quantities based on other attributes in the **Dimension** object or in application meta-data. For example, in nuclear magnetic resonance spectroscopy, the spectra are conventionally plotted as a function of a dimensionless frequency ratio. In CSD model, the origin_offset, *o*_*k*_, is interpreted as the NMR spectrometer frequency and the coordinates_offset, *b*_*k*_, as the reference frequency. Given the dimension coordinate, **X**_*k*_, from Eq (3), the corresponding dimensionless-coordinate ratio follows,

$$\begin{array}{c} X_{k}^{\text{ratio}}=\frac{X_{k}}{o_{k}-b_{k}}. \end{array}$$ (9)

### csdmpy

The csdmpy module is the Python support for the core scientific dataset (CSD) model file-exchange format. The source code is available at https://github.com/DeepanshS/csdmpy and the corresponding documentation at https://csdmpy.readthedocs.io/en/stable, which includes links for downloading the CSDM compliant files used in this report.

The main objective of this python module is to facilitate the import and export of the CSD model serialized files for Python users. Moreover, the module utilizes Python libraries such as Numpy and therefore allowing the end-users to process or visualize the imported datasets with any third-party package(s) compatible with Numpy.

## Supporting information

S1 File Additional CSDM examples and review of units and constants as used in the CSD model. (PDF)
