# Supplementary material for: Core Scientific Dataset Model: A lightweight and portable model and file format for multi-dimensional scientific data
Source: PLoS One. 2020 Jan 2;15(1):e0225953. doi: 10.1371/journal.pone.0225953 (PMC6940021; doi:10.1371/journal.pone.0225953)
Supplement: S1 File — (PDF) [file pone.0225953.s001.pdf]

Supplemental Material for "Core Scientific Dataset Model: A light weight and portable model and file format for multi-dimensional scientific data"

Deepansh J. Srivastava[1], Thomas Vosegaard[2], Dominique Massiot[3], and Philip J. Grandinetti[1]

[1]Department of Chemistry, Ohio State University,
100 West 18th Avenue, Columbus, OH 43210, USA,

[2]Laboratory for Biomolecular NMR Spectroscopy,
Department of Molecular and Structural Biology,
University of Aarhus, DK-8000 Aarhus C, Denmark,

[3]CNRS, UPR3079 CEMHTI,
1D Avenue de la Recherche Scientifique,
45071 Orléans Cedex 2, France

November 22, 2019

In section 1 of this supplement, we give additional serialization examples of the CSD model. In section 2 we give a brief overview of concepts associated with the international system of units[1], along with tables giving unit symbol conventions as well as symbol suggestions for various physical constants.

S1 Additional CSDM Examples

Listings 1, 2, 3, and 4 further illustrate the versatility of the CSD model for 1D{1} datasets from different scientific domains. Here, we give examples from the Fourier-transform infrared (FTIR) spectroscopy, Ultraviolet-visible (UV-vis) spectroscopy, Electron paramagnetic resonance (EPR) spectroscopy, and Gas Chromatography, respectively. These datasets were obtained from http://wwwchem.uwimona.edu.jm/spectra/specindex.html (Prof. Robert J. Lancashire) and converted from JCAMP to the CSD model.

In Listing 5, we show a 2D{1} NMR dataset from a phase-adjusted spinning sideband (PASS) measurement[2] where the two **LinearDimension** objects describe a 2D coordinate grid of time and rotor phase. In Listing 6 we show an example of a 3D{1} NMR dataset for a phase incremented echo train acquisition (PIETA) measurement[3]. Additional details are given in the respective listing captions.

Listing 1: 1D{1} example dataset of the FTIR spectrum of caffeine with one **LinearDimension** object and one **DependentVariable** object. The dimension is sampled every 1.9305486 cm^{-1} for 1842 points starting at 449.41 cm^{-1}. Here, the data values in the **InternalDependentVariable** object are stored as a Base64 encoded string of a 32-bit floating point array.

caffeine.csdf


```
{
 "csdm": {
  "version": "1.0",
  "timestamp": "2019-07-01T21:03:42Z",
  "read_only": true,
  "tags": [
    "infrared spectrum",
    "caffeine"
  ],
  "dimensions": [
    {
      "type": "linear",
      "count": 1842,
      "increment":"1.9305486 cm^-1",
      "coordinates_offset": "449.41 cm^-1",
      "quantity_name": "wavenumber",
    }
  ],
  "dependent_variables": [
    {
      "type": "internal",
      "name": "Caffeine",
      "encoding": "base64",
      "numeric_type": "float32",
      "quantity_type": "scalar",
      "component_labels": ["Transmittance"],
      "components": ["/p7GQg...J5dchC"]
    }
  ]
 }
}
```


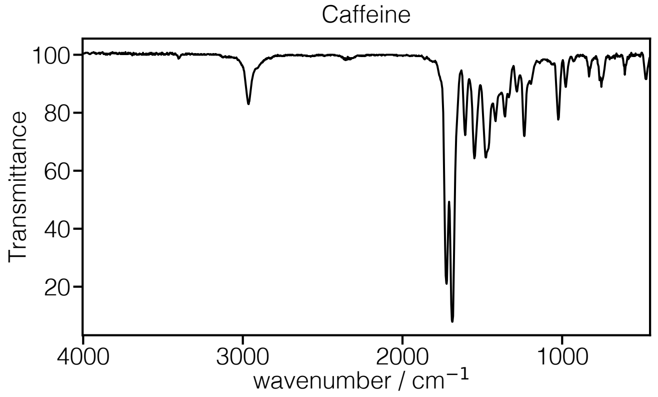

Figure S1: **CSD model depiction of a FTIR dataset.**

Listing 2: 1D{1} example dataset of the UV-vis spectrum of benzene vapor with one **LinearDimension** object and one **DependentVariable** object. The dimension is sampled every 0.01 nm for 4001 data points starting at 230 nm. The data values from the **ExternalDependentVariable** object are stored as 32 bit floating point binary array in an external file whose URL relative to the `benzene.csdfe` file is specified by the value of the components_url key.

benzene.csdfe

```
{
 "csdm": {
  "version": "1.0",
  "timestamp": "2019-07-01T21:03:42Z",
  "read_only": true,
  "tags": [
    "uv-visible spectrum",
    "gas phase",
    "benzene"
  ],
  "dimensions": [
    {
      "type": "linear",
      "count": 4001,
      "increment": "0.01 nm",
      "coordinates_offset": "230.0 nm",
      "quantity_name": "wavelength"
    }
  ],
  "dependent_variables": [
    {
      "type": "external",
      "name": "Vapor of Benzene",
      "numeric_type": "float32",
      "quantity_type": "scalar",
      "component_labels": ["Absorbance"],
      "components_url": "file:./benzeneVap.dat"
    }
  ]
 }
}
```

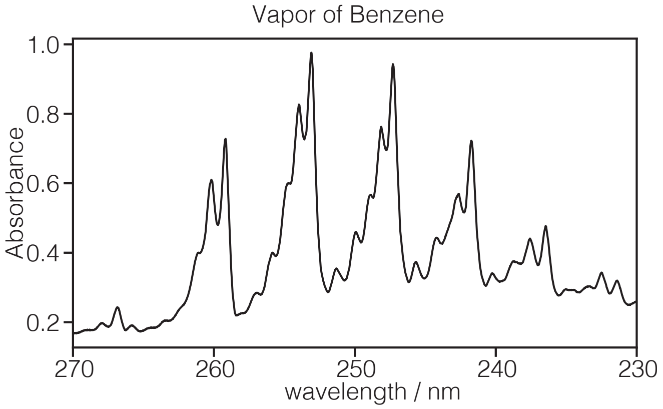

Figure S2: **CSD model depiction of a UV-Vis dataset.**

Listing 3: 1D{1} example dataset of the EPR spectrum of vanadium in the mushroom *Amanita muscaria*[4] with one **InternalDependentVariable** and one **LinearDimension** object. The dimension is sampled for 298 points at every 4 G starting at 2750 G. The data values in the **InternalDependentVariable** object are stored as a Base64 encoded string of a 32-bit floating points array.

AmanitaMuscaria.csdf

```
{
 "csdm": {
  "version": "1.0",
  "timestamp": "2019-07-01T21:03:42Z",
  "read_only": true,
  "tags" : [
    "epr spectrum",
    "Vanadyl",
    "Amanita muscaria"
  ],
  "dimensions": [
    {
      "type": "linear",
      "count": 298,
      "increment": "4.0 G",
      "coordinates_offset": "2750.0 G",
      "quantity_name": "magnetic flux density"
    }
  ],
  "dependent_variables": [
    {
      "type": "internal",
      "name": "Vanadyl in Amanita muscaria",
      "encoding": "base64",
      "numeric_type": "float32",
      "quantity_type": "scalar",
      "component_labels": ["Intensity Derivative"],
      "components": ["TDeJPZ...kMvg=="]
    }
  ]
 }
}
```

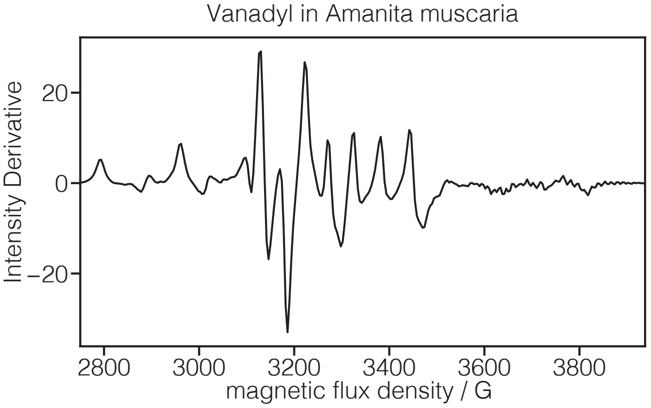

Figure S3: **CSD model depiction of an EPR dataset.**

Listing 4: 1D{1} example dataset of the gas chromatograph of cinnamon with one **InternalDependentVariable** and one **LinearDimension** object. The data values in the **InternalDependentVariable** object are stored as a Base64 encoded string of a 32-bit floating points array.

```
cinnamon.csdf
{
 "csdm": {
  "version": "1.0",
  "timestamp": "2019-07-01T21:03:42Z",
  "read_only": true,
  "tags": [
    "gas chromatography",
    "cinnamon"
  ],
  "dimensions": [
    {
      "count": 6001,
      "increment": "0.0034 min",
      "quantity_name": "time"
    }
  ],
  "dependent_variables": [
    {
      "components": ["AEU9Rw...g7Rw=="],
      "encoding": "base64",
      "numeric_type": "float32",
      "quantity_type": "scalar",
      "name": "Headspace from cinnamon stick",
      "component_labels" : ["FID response"]
    }
  ]
 }
}
```

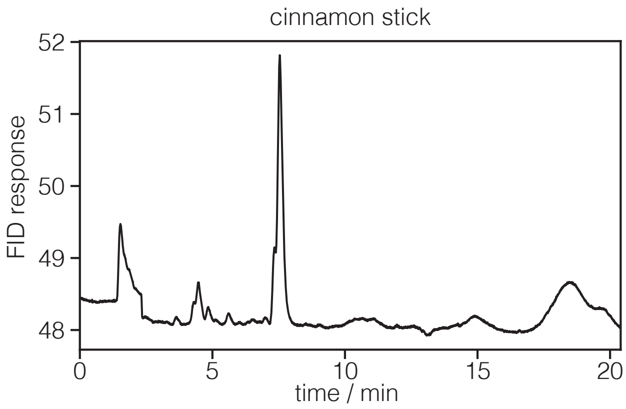

Figure S4: **CSD model depicting of a gas chromatography dataset.**

Listing 5: A 2D{1} example dataset of the NMR phase-adjusted spinning sideband (PASS) measurement[2, 5, 6] where the two **LinearDimension** objects describe a 2D coordinate grid of time and rotor phase. The first **LinearDimension** describes a linearly sampled time dimension with 128 points acquired at every 10 μs. The second **LinearDimension** describes a linearly sampled `rotor phase / 2` πdimension with 32 points sampled at every 0.03125 tr. The symbol "tr" represents a unit of turn. Since this dimension is periodic, a period of 1 tr is provided as the value of the period key. Here, the label in the corresponding **ReciprocalDimension** object is `sideband order`. The value of the dependent_variables key is an array with a single **ExternalDependentVariable** object describing the single-component complex signal response.

pass.csdfe

```
{
 "csdm": {
  "version": "1.0",
  "timestamp": "2010-11-23T22:26:00Z",
  "tags": ["13C NMR", "Phase Adjusted Spinning Sideband", "L-Histidine"],
  "description": "M.C. Davis, et al., J.Magn.Reson., 210, 51-58 (2011).",
  "dimensions": [
    {
      "type": "linear",
      "count": 128,
      "quantity_name": "time",
      "increment": "10 µs",
      "reciprocal": {
        "coordinates_offset": "1229 Hz",
        "origin_offset": "131.543 MHz"
      }
    },
    {
      "type": "linear",
      "count": 32,
      "quantity_name": "plane angle",
      "label": "rotor phase / 2 $\pi$,
      "increment": "0.03125 tr",
      "period": "1 tr",
      "reciprocal": {
        "label": "sideband order"
      }
    }
  ],
  "dependent_variables": [
    {
      "type": "external",
      "numeric_type": complex64",
      "quantity_type": "scalar",
      "components_url": "file:./pass.dat"
    }
  ]
 }
}
```

Listing 6: A 3D{1} example dataset of the NMR phase incremented echo train acquisition (PIETA) measurement[3] with three **LinearDimension** objects. The 1st dimension, labeled as `t2`, is sampled every 15 μs for 256 points starting at 1.92 ms. The 2nd dimension, labeled as `t1 (echo time)`, is sampled every 5 ms for 128 points. The 3rd dimension, labeled as `pulse phase/`2π, is periodic with period as 1 tr, and sampled every 0.0039065 turns for 256 points. Its **ReciprocalDimension** object is labeled as `accumulated coherence order change`. The dependent_variables holds an array with a single **ExternalDependentVariable** object with complex values stored in an external file as a binary array. The component_url gives the external file location on a remote `https` server.

pieta.csdfe

```
{
 "csdm": {
  "version": "1.0",
  "timestamp": "2019-07-01T21:03:42Z",
  "dimensions": [
    {
      "type": "linear",
      "count": 256,
      "quantity_name": "time",
      "increment": "15 µs",
      "coordinates_offset": "-1.92 ms",
      "label": "t2",
      "reciprocal": {
        "origin_offset": "400.065795 MHz",
        "label": "1H frequency shift"}
    },
    {
      "type": "linear",
      "count": 128,
      "increment": "5.0 ms",
      "label": "t1 (echo time)"
    },
    {
      "type": "linear",
      "count": 256,
      "increment": "0.00390625 tr",
      "period": "1.0 tr",
      "label": "pulse phase / 2 $\pi$",
      "reciprocal": {
        "label":"accumulated coherence order change"}
    }
  ],
  "dependent_variables": [
    {
      "type": "external",
      "numeric_type": "complex64",
      "quantity_type": "scalar",
      "components_url": "https://osu.box.com/shared/static/o46g1boeu8w009la6q9jbezks7duymon.data"
    }
  ]
 }
}
```

S2 Overview of Physical Quantities, Units and Constants

S2.1 Dimensionality

In the International System of Units (SI) seven reference quantities are used to define seven dimensions whose symbols are given in Table S1. The dimensionality of any physical quantity, q, can then be expressed in terms of these seven reference dimensions in the form of a dimensional product

$$\dim q = \mathsf{L}^{\alpha} \cdot \mathsf{M}^{\beta} \cdot \mathsf{T}^{\gamma} \cdot \mathsf{I}^{\delta} \cdot \Theta^{\epsilon} \cdot \mathsf{N}^{\zeta} \cdot \mathsf{J}^{\eta}, \tag{1}$$

where the lower case greek symbols represent integers called the dimensional exponents. The dimensionality of any physical quantity can be represented as a point in the space of dimensional exponents $(\alpha, \beta, \gamma, \delta, \epsilon, \zeta, \eta)$. Physical quantities with different meanings can have the same dimensionality. For example, the thermodynamic quantities *entropy* and *heat capacity* are different physical quantities having the same physical dimensions. Only physical quantities with the same dimensionality can be added. With the operation of multiplication, the physical dimensions form a mathematical group.

There also exists dimensionless quantities, such as *plane angle* which has dimensionality of L/L, or *solid angle* with a dimensionality of $\mathsf{L}^2/\mathsf{L}^2$. Representing such dimensionalities requires us to split each dimension exponent into the numerator and denominator exponents. Thus, we redefine our set of dimensionalities to incorporate numerator and denominator exponents,

$$\dim q = \left[\frac{\mathsf{L}^{\alpha_+}}{\mathsf{L}^{\alpha_-}}\right] \cdot \left[\frac{\mathsf{M}^{\beta_+}}{\mathsf{M}^{\beta_-}}\right] \cdot \left[\frac{\mathsf{T}^{\gamma_+}}{\mathsf{T}^{\gamma_-}}\right] \cdot \left[\frac{\mathsf{I}^{\delta_+}}{\mathsf{I}^{\delta_-}}\right] \cdot \left[\frac{\Theta^{\epsilon_+}}{\Theta^{\epsilon_-}}\right] \cdot \left[\frac{\mathsf{N}^{\zeta_+}}{\mathsf{N}^{\zeta_-}}\right] \cdot \left[\frac{\mathsf{J}^{\eta_+}}{\mathsf{J}^{\eta_-}}\right]. \tag{2}$$

A list of accepted physical quantity names in the CSD model and their corresponding dimensionality symbols are given in Table 8. When no accepted physical quantity name exists for a dimensionality the symbol obtained from Eq. (2) should be used as the quantity name in the CSD model.

S2.2 Unit

Inherent in the measurement of any physical quantity is a comparison to a previous measurement. What is most useful is the ratio of the new measurement to a previous measurement. For example, an ancient scientist might have used a cubit to measure the ratio of large tree trunk's circumference to its diameter. Around 3000 B.C.E., a cubit was decreed to be the length of a forearm and hand. So, a scientist could make a string of 1 cubit in length using the distance from the back of her elbow to the tip of her middle finger, and then use the string to measure the ratio

$$\frac{l_{\text{circumference}}}{l_{\text{diameter}}} \approx 3.14. \tag{3}$$

While another scientist with longer arms might have cut a longer string to be a cubit, the procedure for finding the ratio of large tree trunk's circumference to its diameter will be the same, and the result is independent of the absolute length of the string used, i.e., independent of the units of length used.

S2.2.1 SI Units

We represent a physical quantity, q, using the notation

$$q = \{q\} \cdot [q], \tag{4}$$

where $\{q\}$ is the numerical value and $[q]$ is the reference unit.

Reference Quantity	Dimension Symbol
length	L
mass	M
time	T
electric current	I
thermodynamic temperature	Θ
amount of substance	N
luminous intensity	J

Table S1: Dimension symbols for the seven reference quantities.

Base Dimension	Coherent SI Base Unit Name	Plural Name	Symbol
length	meter	meters	m
mass	kilogram	kilograms	kg
time	second	seconds	s
electric current	ampere	ampere	A
thermodynamic temperature	kelvin	kelvin	K
amount of substance	mole	moles	mol
luminous intensity	candela	candelas	cd

Table S2: Coherent SI base units for the seven reference quantities.

Base Dimension	Base Root Unit Name	Plural Name	Symbol
length	meter	meters	m
mass	gram	grams	g
time	second	seconds	s
electric current	ampere	ampere	A
thermodynamic temperature	kelvin	kelvin	K
amount of substance	mole	moles	mol
luminous intensity	candela	candelas	cd

Table S3: Base root unit names and symbols for the seven reference quantities.

Coherent SI Base Units: The *coherent SI base (reference) units* form a set of seven units, described in Table S2 and given by the symbols

$$[q]_{\mathrm{CBU}} \in [Q]_{\mathrm{CBU}} = \{\mathrm{m}, \mathrm{kg}, \mathrm{s}, \mathrm{A}, \mathrm{K}, \mathrm{mol}, \mathrm{cd}\}\,. \tag{5}$$

SI Base Root Units: A minor complication is that the coherent base unit for mass in SI Units is defined as the kilogram and not the gram. For this reason, we define the set of seven base root units,

$$[q]_{\mathrm{BRU}} \in [Q]_{\mathrm{BRU}} = \{\mathrm{m}, \mathrm{g}, \mathrm{s}, \mathrm{A}, \mathrm{K}, \mathrm{mol}, \mathrm{cd}\}\,. \tag{6}$$

with names and symbols shown in table S3.

SI Base Units: The set of *Coherent SI Base Units* only includes the seven SI base units, given in Table S2. The larger set of *SI Base Units* includes the Coherent SI Base Units as well as all decimal multiples of the root units created using the 20 prefix symbols given in Table S4 with the root unit names and symbols given in Table S3. These prefixed and unprefixed symbols form the set, $[Q]_{\mathrm{BU}}$, of 147 SI base units,

$$[q]_{\mathrm{BU}} \in [Q]_{\mathrm{BU}} = \{x_L\mathrm{m}, x_M\mathrm{g}, x_T\mathrm{s}, x_I\mathrm{A}, x_\Theta\mathrm{K}, x_N\mathrm{mol}, x_J\mathrm{cd}\}\,, \tag{7}$$

where $[Q]_{\mathrm{CBU}} \subseteq [Q]_{\mathrm{BU}}$, $[Q]_{\mathrm{BRU}} \subseteq [Q]_{\mathrm{BU}}$, and x_i indicates that the root unit symbol may be modified with one of the SI prefixes given in Table S4.

Coherent Derived SI Units: *Coherent derived SI units* are an infinite set, $[Q]_{\mathrm{CDU}}$, defined as the products of powers of *coherent SI base units*,

$$[q]_{\mathrm{CDU}} \in [Q]_{\mathrm{CDU}} = \{\mathrm{m}^\alpha \cdot \mathrm{kg}^\beta \cdot \mathrm{s}^\gamma \cdot \mathrm{A}^\delta \cdot \mathrm{K}^\epsilon \cdot \mathrm{mol}^\zeta \cdot \mathrm{cd}^\eta\}, \tag{8}$$

for all positive and negative integer values of the exponents. Here $[Q]_{\mathrm{CBU}} \subseteq [Q]_{\mathrm{CDU}}$.

Derived SI Root Units: *Derived SI root units* are an infinite set, $[Q]_{\mathrm{DRU}}$, defined as the products of powers of *SI base root units*,

$$[q]_{\mathrm{DRU}} \in [Q]_{\mathrm{DRU}} = \{\mathrm{m}^\alpha \cdot \mathrm{g}^\beta \cdot \mathrm{s}^\gamma \cdot \mathrm{A}^\delta \cdot \mathrm{K}^\epsilon \cdot \mathrm{mol}^\zeta \cdot \mathrm{cd}^\eta\}, \tag{9}$$

for all positive and negative integer values of the exponents. Here $[Q]_{\mathrm{BRU}} \subseteq [Q]_{\mathrm{DRU}}$.

SI Prefix Name	x, SI Prefix Symbol	factor	SI Prefix Name	x, SI Prefix Symbol	factor
yotta	Y	10^{24}	yocto	y	10^{-24}
zetta	Z	10^{21}	zepto	z	10^{-21}
exa	E	10^{18}	atto	a	10^{-18}
peta	P	10^{15}	femto	f	10^{-15}
tera	T	10^{12}	pico	p	10^{-12}
giga	G	10^{9}	nano	n	10^{-9}
mega	M	10^{6}	micro	μ	10^{-6}
kilo	k	10^{3}	milli	m	10^{-3}
hecto	h	10^{2}	centi	c	10^{-2}
deca	da	10^{1}	deci	d	10^{-1}

Table S4: SI prefixes used for the seven reference quantities.

Derived SI Units: *Derived SI units* are an infinite set, $[Q]_{\mathrm{DU}}$, defined as the products of powers of *SI base units*,

$$[q]_{\mathrm{DU}} \in [Q]_{\mathrm{DU}} = \left\{ (x_L\mathrm{m})^{\alpha} \cdot (x_M\mathrm{g})^{\beta} \cdot (x_T\mathrm{s})^{\gamma} \cdot (x_I\mathrm{A})^{\delta} \cdot (x_\Theta\mathrm{K})^{\epsilon} \cdot (x_N\mathrm{mol})^{\zeta} \cdot (x_J\mathrm{cd})^{\eta} \right\}, \tag{10}$$

for all positive and negative integer values of the exponents. Here $[Q]_{\mathrm{CBU}} \subseteq [Q]_{\mathrm{CDU}} \subseteq [Q]_{\mathrm{DU}}$ and $[Q]_{\mathrm{BRU}} \subseteq [Q]_{\mathrm{DRU}} \subseteq [Q]_{\mathrm{DU}}$.

Derived SI Dimensionless Units: There also exists dimensionless units, such as the radian which has units of m/m, or the steradian with units of $\mathrm{m}^2/\mathrm{m}^2$. Representing such units requires us to split each dimension exponent into the numerator and denominator exponents. Thus, we redefine the infinite set of coherent derived SI units to incorporate the numerator and denominator exponents,

$$[q]_{\mathrm{CDU}} \in [Q]_{\mathrm{CDU}} = \left\{ \left[\frac{\mathrm{m}^{\alpha_+}}{\mathrm{m}^{\alpha_-}}\right] \cdot \left[\frac{\mathrm{kg}^{\beta_+}}{\mathrm{kg}^{\beta_-}}\right] \cdot \left[\frac{\mathrm{s}^{\gamma_+}}{\mathrm{s}^{\gamma_-}}\right] \cdot \left[\frac{\mathrm{A}^{\delta_+}}{\mathrm{A}^{\delta_-}}\right] \cdot \left[\frac{\mathrm{K}^{\epsilon_+}}{\mathrm{K}^{\epsilon_-}}\right] \cdot \left[\frac{\mathrm{mol}^{\zeta_+}}{\mathrm{mol}^{\zeta_-}}\right] \cdot \left[\frac{\mathrm{cd}^{\eta_+}}{\mathrm{cd}^{\eta_-}}\right] \right\}, \tag{11}$$

for all positive integer values of the exponent numerator and denominators. Using this approach we represent dimensionless units with an exponent vector using the notation $(\alpha_+{-}\alpha_-,\ \beta_+{-}\beta_-,\ \gamma_+{-}\gamma_-,\ \delta_+{-}\delta_-,\ \epsilon_+{-}\epsilon_-,\ \zeta_+{-}\zeta_-,\ \eta_+{-}\eta_-)$. Thus, the radian can be represented by the exponent vector (1−1, 0−0, 0−0, 0−0, 0−0, 0−0, 0−0) and the steradian by (2−2, 0−0, 0−0, 0−0, 0−0, 0−0, 0−0). Dimensionless quantities such as counts, are not derived SI units and are represented with the exponent vector $(0, 0, 0, 0, 0, 0, 0)$.

Finally, we redefine the infinite set of derived SI units to incorporate numerator and denominator exponents,

$$[q]_{\mathrm{DU}} \in [Q]_{\mathrm{DU}} = \left\{ \left[\frac{(x_L^+\mathrm{m})^{\alpha_+}}{(x_L^-\mathrm{m})^{\alpha_-}}\right] \cdot \left[\frac{(x_M^+\mathrm{g})^{\beta_+}}{(x_M^-\mathrm{g})^{\beta_-}}\right] \cdot \left[\frac{(x_T^+\mathrm{s})^{\gamma_+}}{(x_T^-\mathrm{s})^{\gamma_-}}\right] \cdot \left[\frac{(x_I^+\mathrm{A})^{\delta_+}}{(x_I^-\mathrm{A})^{\delta_-}}\right] \cdot \left[\frac{(x_\Theta^+\mathrm{K})^{\epsilon_+}}{(x_\Theta^-\mathrm{K})^{\epsilon_-}}\right] \cdot \left[\frac{(x_N^+\mathrm{mol})^{\zeta_+}}{(x_N^-\mathrm{mol})^{\zeta_-}}\right] \cdot \left[\frac{(x_J^+\mathrm{cd})^{\eta_+}}{(x_J^-\mathrm{cd})^{\eta_-}}\right] \right\}, \tag{12}$$

for all positive integer values of the exponent numerator and denominators. The infinite set $[Q]_{\mathrm{DU}}$, as described by Eq. (12), is a complete set of units for all quantities in the physical sciences and is allowed in the CSD model.

Equivalent Units While all units in the set $[Q]_{\mathrm{DU}}$ have unique derived symbols and names, it should be noted that some are functionally equivalent. For example, a derived physical quantity such as speed in units of $[\mathrm{m} \cdot \mathrm{s}^{-1}]$ can be converted among any of the units below,

$$[\mathrm{m} \cdot \mathrm{s}^{-1}] \equiv [\mathrm{km} \cdot \mathrm{ks}^{-1}] \equiv [\mathrm{hm} \cdot \mathrm{hs}^{-1}] \equiv [\mathrm{dam} \cdot \mathrm{das}^{-1}] \equiv [\mathrm{dm} \cdot \mathrm{ds}^{-1}] \equiv [\mathrm{cm} \cdot \mathrm{cs}^{-1}] \equiv [\mathrm{nm} \cdot \mathrm{ns}^{-1}], \tag{13}$$

without modifying the numerical value of the quantity.

Special SI Units: In the SI system there is a set, $[Q]_{\mathrm{SU}}$, of 22 coherent derived units contained within the $[Q]_{\mathrm{CDU}}$ set that, for convenience, have their own special names and symbols. The 22 special names and symbols and their corresponding coherent derived units are given in table S5. For example, the coherent derived SI unit: $\mathrm{m} \cdot \mathrm{kg} \cdot \mathrm{s}^{-2}$, used for the derived quantity of force, is given the special name "newton" and replaced with the symbol "N".

	SI Special Unit			
Derived Quantity	Name	Plural Name	Symbol	Coherent Derived SI Symbol
plane angle	radian	radians	rad	(m/m)
solid angle	steradian	steradians	sr	(m^2/m^2)
frequency	hertz	hertz	Hz	s^{-1}
force	newton	newtons	N	$m \cdot kg \cdot s^{-2}$
pressure, stress	pascal	pascals	Pa	$m^{-1} \cdot kg \cdot s^{-2}$
energy, work, heat	joule	joules	J	$m^2 \cdot kg \cdot s^{-2}$
power, radiant flux	watt	watts	W	$m^2 \cdot kg \cdot s^{-1}$
electric charge	coulomb	coulombs	C	$s \cdot A$
electric potential difference	volt	volts	V	$m^2 \cdot kg \cdot s^{-3} \cdot A^{-1}$
capacitance	farad	farads	F	$m^{-2} \cdot kg^{-1} \cdot s^4 \cdot A^2$
resistance	ohm	ohms	Ω	$m^2 \cdot kg \cdot s^{-3} \cdot A^{-2}$
electric conductance	siemens	siemens	S	$m^{-2} \cdot kg^{-1} \cdot s^3 \cdot A^2$
magnetic flux	weber	webers	Wb	$m^2 \cdot kg \cdot s^{-2} \cdot A^{-1}$
magnetic flux density	tesla	tesla	T	$kg \cdot s^{-2} \cdot A^{-1}$
inductance	henry	henry	H	$m^2 \cdot kg \cdot s^{-2} \cdot A^{-2}$
Celsius Temperature	degree Celsius	degrees Celsius	°C	K
luminous flux	lumen	lumens	lm	$(m^2/m^2) \cdot cd$
illuminance	lux	lux	lx	$m^{-2} \cdot cd$
radionuclide activity	becquerel	becquerel	Bq	s^{-1}
absorbed dose	gray	grays	Gy	$m^2 \cdot s^{-2}$
dose equivalent	sievert	sieverts	Sv	$m^2 \cdot s^{-2}$
catalytic activity	katal	katal	kat	$s^{-1} \cdot mol$

Table S5: The 22 Coherent Derived SI Units with Special SI Names and Symbols.

Special SI Units with prefixes: The 22 coherent derived units with special SI units have the additional possibility of being modified by SI prefixes to create a set, $[Q]_{x\mathrm{SU}}$, of 462 units. Some of the units in $[Q]_{x\mathrm{SU}}$, however, may not have an equivalent in the set of derived SI units, $[Q]_{\mathrm{DU}}$. That is, $[Q]_{\mathrm{DU}} - [Q]_{x\mathrm{SU}} \neq \emptyset$. For example, the Sievert is equivalent to the coherent derived unit $m^2 \cdot s^{-2}$, and

$$[\mathrm{Sv}] = [\mathrm{m}^2 \cdot \mathrm{s}^{-2}] \subseteq [Q]_{\mathrm{DU}}, \tag{14}$$

but the decisievert, [dSv], which is one-tenth of the coherent derived unit $m^2 \cdot s^{-2}$, has no equivalent unit in the set $[Q]_{\mathrm{DU}}$. That is,

$$[\mathrm{dSv}] = 0.1[\mathrm{m}^2 \cdot \mathrm{s}^{-2}] \nsubseteq [Q]_{\mathrm{DU}}. \tag{15}$$

On the other hand, the centiSievert, [cSv], which is one-hundredth of the coherent derived unit $m^2 \cdot s^{-2}$, has an equivalent unit in the set $[Q]_{\mathrm{DU}}$,

$$[\mathrm{cSv}] = 0.01[\mathrm{m}^2 \cdot \mathrm{s}^{-2}] = [\mathrm{dm}^2 \cdot \mathrm{s}^{-2}] = [\mathrm{m}^2 \cdot \mathrm{das}^{-2}] \subseteq [Q]_{\mathrm{DU}}. \tag{16}$$

Derived Units employing Special SI Units: With the introduction of special SI symbols there is the additional possibility of derived quantities with derived units employing a special SI unit. Generally, however, no more than one special SI symbol appears in such a derived unit symbol and the special SI symbol often appears as a linear term in the numerator. For example, the derived quantity dynamic viscosity can use the derived unit $[\mathrm{Pa} \cdot \mathrm{s}]$ as well as $[\mathrm{m}^{-1} \cdot \mathrm{kg} \cdot \mathrm{s}^{-1}]$. The derived quantity of surface tension can use the derived unit $[\mathrm{N} \cdot \mathrm{m}^{-1}]$ as well as $[\mathrm{kg} \cdot \mathrm{s}^{-2}]$. Additional examples are given in Table S6.

S2.2.2 Non-SI Units:

There are several units outside the International System of Units that continue to be used in different science and engineering communities. While it is straightforward to include non-SI unit symbols in the same manner as special SI unit symbols, one has to be careful to avoid symbol and name collisions, particularly as some non-SI unit symbols employ SI prefixes.

Derived Quantity	Derived Symbol	Coherent Derived SI Symbol
dynamic viscosity	$\mathrm{Pa \cdot s}$	$\mathrm{m^{-1} \cdot kg \cdot s^{-1}}$
moment of force	$\mathrm{N \cdot m}$	$\mathrm{m^2 \cdot kg \cdot s^{-2}}$
surface tension	N/m	$\mathrm{kg \cdot s^{-2}}$
angular velocity	rad/s	$\mathrm{m/(m \cdot s)}$
angular acceleration	$\mathrm{rad/s^2}$	$\mathrm{m/(m \cdot s^2)}$
heat flux density, irradiance	$\mathrm{W/m^2}$	$\mathrm{kg \cdot s^{-3}}$
heat capacity, entropy	J/K	$\mathrm{m^2 \cdot kg \cdot s^{-2} \cdot K^{-1}}$
specific heat capacity, specific entropy	$\mathrm{J/(kg \cdot K)}$	$\mathrm{m^2 \cdot s^{-2} \cdot K^{-1}}$
specific energy	J/kg	$\mathrm{m^2 \cdot s^{-2}}$
thermal conductivity	$\mathrm{W/(m \cdot K)}$	$\mathrm{m \cdot kg \cdot s^{-3} \cdot K^{-1}}$
energy density	$\mathrm{J/m^3}$	$\mathrm{m^{-1} \cdot kg \cdot s^{-2}}$
electric field strength	V/m	$\mathrm{m \cdot kg \cdot s^{-3} \cdot A^{-1}}$
electric charge density	$\mathrm{C/m^3}$	$\mathrm{m^{-3} \cdot s \cdot A}$
surface charge density	$\mathrm{C/m^2}$	$\mathrm{m^{-2} \cdot s \cdot A}$
electric flux density, electric displacement	$\mathrm{C/m^2}$	$\mathrm{m^{-2} \cdot s \cdot A}$
permittivity	F/m	$\mathrm{m^{-3} \cdot kg^{-1} \cdot s^4 \cdot A^2}$
permeability	H/m	$\mathrm{m \cdot kg \cdot s^{-2} \cdot A^{-2}}$
molar energy	J/mol	$\mathrm{m^2 \cdot kg \cdot s^{-2} \cdot mol^{-1}}$
molar entropy, molar heat capacity	$\mathrm{J/(mol \cdot K)}$	$\mathrm{m^2 \cdot kg \cdot s^{-2} \cdot K^{-1} \cdot mol^{-1}}$
exposure (x- and γ-rays)	C/kg	$\mathrm{kg^{-1} \cdot s \cdot A}$
absorbed dose rate	Gy/s	$\mathrm{m^2 \cdot s^{-3}}$
radiant intensity	W/sr	$\mathrm{m^4 \cdot m^{-2} \cdot kg \cdot s^{-3}}$
radiance	$\mathrm{W/(m^2 \cdot sr)}$	$\mathrm{m^2 \cdot m^{-2} \cdot kg \cdot s^{-3}}$
catalytic activity concentration	$\mathrm{kat/m^3}$	$\mathrm{m^{-3} \cdot s^{-1} \cdot mol}$

Table S6: Quantities with Derived Symbols using Special SI Symbols.

S2.2.3 Physical constants

It is also possible to use symbols for physical constants as unit symbols, although again one has to be careful to avoid symbol collisions. There are conflicts between unit symbols and commonly accepted symbols for physical constants, e.g., the planck constant and hour (h), or the newton gravitational constant and gauss (G). Table S7 gives a list of suggested symbols for physical constants (giving priority to unit symbols) which avoid collision with accepted unit symbols.

Due of the symbol collision between the euler constant $e = 2.718281828459045...$ *all string representations of numbers using scientific notation in the serialization of the CSD model should use an upper case* `"E"`, e.g., `6.022140857E+23` instead of `6.022140857e+23`. Since the euler constant is dimensionless the latter expression could mistakenly be interpreted as the valid equation: $6.022140857 \times e + 23 = 39.36987606000388...$

References

[1] Ambler Thompson and Barry N. Taylor. Guide for the use of the International System of Units (SI). March 30, 2008 2008.

[2] W. T. Dixon. Spinning-sideband-free NMR-spectra. *J. Magn. Reson.*, 44:220–223, 1981.

[3] J. H. Baltisberger, B. J. Walder, E. G. Keeler, D. C. Kaseman, K. J. Sanders, and P. J. Grandinetti. Phase incremented echo train acquisition in NMR spectroscopy. *J. Chem. Phys.*, 136:211104–1–4, 2012.

[4] Rupert D. Gillard and Robert J. Lancashire. Electron spin resonance of vanadium in amanita muscaria. *Phytochemistry*, 23:179 – 180, 1984.

[5] O. N. Antzutkin, S. C. Shekar, and M. H. Levitt. Two-dimensional sideband separation in magic-angle spinning NMR. *J. Magn. Reson. A*, 115:7–19, 1995.

Constant	Suggested symbol	Value in Coherent Derived SI Unit
avogadro constant	N_A	6.022140857E+23 (1/mol)
bohr magneton	µ_B	9.274009992054043E-24 m^2*A
boltzmann constant	k_B	1.38064852E-23 m^2*kg/(s^2*K)
characteristic impedance of vacuum	Z_0	376.7303134617707 m^2*kg/(s^3*A^2)
compton wavelength	λ_C	2.42631023609262E-12 m
electric constant	ϵ_0	8.854187817620413E-12 s^4*A^2/(m^3*kg)
electron *g* factor	g_e	-2.00231930436182 m^2*A/(m^2*A)
electron magnetic moment	µ_e	-9.28476462E-24 m^2*A
electron mass	m_e	9.10938356E-31 kg
elementary charge	q_e	1.6021766208E-19 s*A
euler constant	e	2.718281828459045 ...
fine structure constant	α	0.007297352566206478 m^5*kg*s^4*A^2/(m^5*kg*s^4*A^2)
gas constant	R	8.314459861448581 m^2*kg/(s^2*K*mol)
newton gravitational constant	G_N	6.67408E-11 m^3/(kg*s^2)
gravity acceleration	g_0	9.80665 m/s^2
magnetic constant	µ_0	1.256637061435917E-06 m*kg/(s^2*A^2)
magnetic flux quantum	Φ_0	2.067833831170082E-15 m^2*kg/(s^2*A)
muon *g* factor	g_µ	-2.0023318418 m^2*A/(m^2*A)
muon magnetic moment	µ_µ	-4.49044826E-26 m^2*A
myon mass	m_µ	1.883531594E-28 kg
neutron *g* factor	g_n	-3.82608545 m^2*A/(m^2*A)
neutron magnetic moment	µ_n	-9.662365E-27 m^2*A
neutron mass	m_n	1.674927471E-27 kg
nuclear magneton	µ_N	5.050783698211084E-27 m^2*A
pi	π	3.141592653589793 m/m
planck constant	h_P	6.62607004E-34 m^2*kg/s
proton *g* factor	g_p	5.585694702 m^2*A/(m^2*A)
proton magnetic moment	µ_p	1.4106067873E-26 m^2*A
proton mass	m_p	1.672621898E-27 kg
reduced planck constant	$\hbar$	1.054571800139113E-34 m^3*kg/(m*s)
rydberg constant	R_∞	10973731.5705508 (1/m)
speed of light	c_0	299792458 m/s
stefan-boltzmann constant	σ	5.670367E-08 m^2*kg/(m^2*s^3*K^4)
wien wavelength displacement constant	b_λ	0.0028977729 m*K

Table S7: Physical constants and suggested symbols for avoiding symbol collision with unit symbols.

[6] M.C. Davis, K. M. Shookman, J. D. Sillaman, and P. J. Grandinetti. TOP-PASS: A processing algorithm to reduce 2D PASS acquisition time. *J. Magn. Reson*, 210:51–58, 2011.

Table 8: Physical quantity names accepted in the CSD model.

**Quantity**	**Dimensionality**
absorbed dose	L^2•M/(M•T^2)
absorbed dose rate	L^2/T^3
acceleration	L/T^2
action	L^2•M/T
amount	N
amount concentration	N/L^3
amount of electricity	T•I
amount ratio	N/N
angular acceleration	L/(L•T^2)
angular frequency	L/(L•T)
angular momentum	L^2•M/T
angular speed	L/(L•T)
angular velocity	L/(L•T)
area	L^2
area ratio	L^2/L^2
capacitance	T^4•I^2/(L^2•M)
catalytic activity	N/T
catalytic activity concentration	N/(L^3•T)
catalytic activity content	N/(M•T)
charge to amount ratio	T•I/N
charge to mass ratio	T•I/M
circulation	L^2/T
compressibility	L•T^2/M
current	I
current density	I/L^2
current ratio	I/I
density	M/L^3
diffusion coefficient	L^2/T
diffusion flux	N/(L^2•T)
dimensionless	1
distance per volume	L/L^3
dose equivalent	L^2•M/(M•T^2)
dynamic viscosity	M/(L•T)
elastic modulus	M/(L•T^2)
electric charge	T•I
electric charge density	T•I/L^3
electric conductance	T^3•I^2/(L^2•M)
electric conductivity	T^3•I^2/(L^3•M)
electric dipole moment	L•T•I
electric displacement	T•I/L^2
electric field gradient	L^2•M/(L^2•T^3•I)
electric field strength	L•M/(T^3•I)
electric flux	L^3•M/(T•I)
electric flux density	T•I/L^2
electric polarizability	L^2•T^4•I^2/(L^2•M)
electric potential difference	L^2•M/(T^3•I)
electric quadrupole moment	L^2•T•I
electric resistance	L^2•M/(T^3•I^2)
electric resistance per length	L^2•M/(L•T^3•I^2)
electric resistivity	L^3•M/(T^3•I^2)
electrical mobility	L^2•T^3•I/(L^2•M•T)
electromotive force	L^2•M/(T^3•I)
energy	L^2•M/T^2
energy density	M/(L•T^2)
entropy	L^2•M/(T^2•θ)
fine structure constant	L^5•M•T^4•I^2/(L^5•M•T^4•I^2)
first hyperpolarizability	L^3•T^7•I^3/(L^4•M^2)

Quantity	Dimensionality
fluidity	L•T/M
force	L•M/T^2
frequency	1/T
frequency per electric field gradient	L^2•T^3•I/(L^2•M•T)
frequency per electric field gradient squared	L^4•T^6•I^2/(L^4•M^2•T)
frequency per magnetic flux density	T•I/M
frequency ratio	T/T
gas permeance	L•T^2•N/(L^2•M•T)
gravitational constant	L^3/(M•T^2)
gyromagnetic ratio	L•T^2•I/(L•M•T)
heat capacity	L^2•M/(T^2•θ)
heat flux density	L^2•M/(L^2•T^3)
heat transfer coefficient	M/(T^3•θ)
illuminance	L^2•J/L^4
inductance	L^2•M/(T^2•I^2)
inverse amount	1/N
inverse amount concentration inverse time	L^3/(T•N)
inverse area	1/L^2
inverse current	1/I
inverse length	1/L
inverse luminous intensity	1/J
inverse magnetic flux density	T^2•I/M
inverse mass	1/M
inverse temperature	1/θ
inverse time	1/T
inverse time squared	1/T^2
inverse volume	1/L^3
irradiance	L^2•M/(L^2•T^3)
kinematic viscosity	L^2/T
length	L
length ratio	L/L
linear momentum	L•M/T
luminance	J/L^2
luminous efficacy	T^3•J/(L^2•M)
luminous energy	L^2•T•J/L^2
luminous flux	L^2•J/L^2
luminous flux density	L^2•J/L^4
luminous intensity	J
luminous intensity ratio	J/J
magnetic dipole moment	L^2•I
magnetic dipole moment ratio	L^2•I/(L^2•I)
magnetic field gradient	M/(L•T^2•I)
magnetic field strength	I/L
magnetic flux	L^2•M/(T^2•I)
magnetic flux density	M/(T^2•I)
magnetizability	L^2•M•T^4•I^2/(M^2•T^2)
mass	M
mass concentration	M/L^3
mass flow rate	M/T
mass flux	M/(L^2•T)
mass ratio	M/M
mass to charge ratio	M/(T•I)
molality	N/M
molar conductivity	L^2•T^3•I^2/(L^2•M•N)
molar energy	L^2•M/(T^2•N)
molar entropy	L^2•M/(T^2•θ•N)
molar heat capacity	L^2•M/(T^2•θ•N)
molar magnetic susceptibility	L^3/N
molar mass	M/N

Quantity	Dimensionality
moment of force	L^2•M^2/(M•T^2)
moment of inertia	L^2•M
permeability	L•M/(T^2•I^2)
permittivity	T^4•I^2/(L^3•M)
plane angle	L/L
porosity	L^3/L^3
power	L^2•M/T^3
power per luminous flux	L^3•M/(L•T^3•J)
pressure	M/(L•T^2)
pressure gradient	M/(L^2•T^2)
radiance	L^4•M/(L^4•T^3)
radiant flux	L^2•M/T^3
radiant intensity	L^4•M/(L^2•T^3)
radiation exposure	T•I/M
radioactivity	1/T
reduced action	L^3•M/(L•T)
refractive index	L•T/(L•T)
rock permeability	L^2
second hyperpolarizability	L^4•T^10•I^4/(L^6•M^3)
second radiation constant	L^3•M•T^2•θ/(L^2•M•T^2)
solid angle	L^2/L^2
specific energy	L^2/T^2
specific entropy	L^2/(T^2•θ)
specific gravity	L^3•M/(L^3•M)
specific heat capacity	L^2/(T^2•θ)
specific power	L^2•M/(M•T^3)
specific surface area	L^2/M
specific volume	L^3/M
spectral power	L^2•M/(L•T^3)
spectral radiance	L^4•M/(L^5•T^3)
spectral radiant energy	L^2•M/(L•T^2)
spectral radiant flux density	M/(L•T^3)
spectral radiant intensity	L^4•M/(L^3•T^3)
speed	L/T
stefan-boltzmann constant	L^2•M/(L^2•T^3•θ^4)
stress	M/(L•T^2)
stress-optic coefficient	L•T^2/M
surface area to volume ratio	L^2/L^3
surface charge density	T•I/L^2
surface density	M/L^2
surface energy	L^2•M/(L^2•T^2)
surface tension	M/T^2
temperature	θ
temperature gradient	θ/L
temperature ratio	θ/θ
thermal conductance	L^2•M/(T^3•θ)
thermal conductivity	L•M/(T^3•θ)
time	T
time ratio	T/T
torque	L^3•M/(L•T^2)
velocity	L/T
voltage	L^2•M/(T^3•I)
volume	L^3
volume per length	L^3/L
volume power density	L^2•M/(L^3•T^3)
volume ratio	L^3/L^3
volumetric flow rate	L^3/T
wavelength displacement constant	L•θ
wavenumber	1/L

Table 9: Physical unit symbols accepted in the CSD model.

Unit or constant name	Symbol	SI Prefix	Value in Coherent Derived SI Unit
acres	ac	No	4046.8564224 m^2
acre feet	ac*ft	No	1233.48183754752 m^3
alpha particle mass	m_a	No	6.64465723E-27 kg
alpha particle mass energy	m_a*c_0^2	No	5.971920096393537E-10 kg
amperes	A	Yes	1 A
ampere hours	A*h	No	3600 s*A
ampere hours per gram	A*h/g	No	3600000 s*A/kg
ampere minutes	A*min	No	60 s*A
ampere minutes per gram	A*min/g	No	60000 s*A/kg
amperes per ampere	A/A	Yes	1 A/A
ampere per meter	A/m	Yes	1 A/m
amperes per square meter	A/m^2	Yes	1 A/m^2
ampere seconds	A*s	No	1 s*A
ampere seconds per gram	A*s/g	No	1000 s*A/kg
ampere square meters	A*m^2	Yes	1 m^2*A
astronomical units	ua	No	149597870691 m
atmospheres	atm	No	101325 kg/(m*s^2)
atomic mass constant	m_u	No	1.66053904E-27 kg
atomic mass units	u	No	1.66053904E-27 kg
atomic units of 1st polarizability	q_e^3*a_0^3/E_h^2	No	3.206361325744867E-53 m^3*s^7*A^3/(m^4*kg^2)
atomic units of 2nd polarizability	q_e^4*a_0^4/E_h^3	No	6.235380075451029E-65 m^4*s^10*A^4/(m^6*kg^3)
atomic unit of charge density	q_e/a_0^3	No	1081202377737.567 s*A/m^3
atomic unit of current	q_e*E_h/ℏ	No	0.00662361818438244 A
atomic unit of electric dipole moment	q_e*a_0	No	8.478353550255471E-30 m*s*A
atomic unit of electric field	E_h/(q_e*a_0)	No	514220670904.8027 m*kg/(s^3*A)
atomic unit of electric field gradient	E_h/(q_e*a_0^2)	No	9.717362362541966E+21 m^2*kg/(m^2*s^3*A)
atomic unit of electric field gradient	Λ_0	No	9.717362362541966E+21 m^2*kg/(m^2*s^3*A)
atomic units of electric polarizability	q_e^2*a_0^2/E_h	No	1.648777272087738E-41 m^2*s^4*A^2/(m^2*kg)
atomic units of electric potential	E_h/q_e	No	27.2113860243671 m^2*kg/(s^3*A)
atomic units of electric quadrupole moment	q_e*a_0^2	No	4.486551481898239E-40 m^2*s*A
atomic unit of energy	E_h	No	4.359744650780484E-18 m^2*kg/s^2
atomic units of force	E_h/a_0	No	8.238723368557656E-08 m*kg/s^2
atomic unit of length	a_0	No	5.291772105638424E-11 m
atomic units of magnetic dipole moment	ℏ*q_e/m_e	No	1.854801998410809E-23 m^2*A
atomic units of magnetic flux density	ℏ/(q_e*a_0^2)	No	235051.755093854 kg/(s^2*A)
atomic units of magnetizability	q_e*a_0^2/m_e	No	4.925197684724827E-10 m^2*kg*s^4*A^2/(kg^2*s^2)
atomic units of momentum	ℏ/a_0	No	1.992851882293756E-24 m*kg/s
atomic units of permittivity	q_e^2/(a_0*E_h)	No	1.112650056053621E-10 s^4*A^2/(m^3*kg)
atomic units of time	ℏ/E_h	No	2.41888432605869E-17 s
atomic units of velocity	a_0*E_h/ℏ	No	2187691.262715648 m/s
avogadro constant	N_A	No	6.022140857E+23 (1/mol)
bars	bar	Yes	100000 kg/(m*s^2)
barns	b	No	1E-28 m^2
becquerels	Bq	Yes	1 (1/s)
bohr magnetons	µ_B	No	9.274009992054043E-24 m^2*A
boltzmann constant	k_B	No	1.38064852E-23 m^2*kg/(s^2*K)

Unit or constant name	Symbol	SI Prefix	Value in Coherent Derived SI Unit
brewsters	B	No	1E-12 m*s^2/kg
british thermal units	Btu	No	1055.05585257348 m^2*kg/s^2
british thermal unit per hour	Btu/h	No	0.2930710701593 m^2*kg/s^3
british thermal unit per minute	Btu/min	No	17.584264209558 m^2*kg/s^3
british thermal unit per second	Btu/s	No	1055.05585257348 m^2*kg/s^3
Btus per hour per foot per rankine	Btu/(h*ft*°R)	No	1.730734666295077 m*kg/(s^3*K)
Btus per hour per rankine	Btu/(h*°R)	No	0.5275279262867396 m^2*kg/(s^3*K)
Btus per hour per square foot per rankine	Btu/(h*ft^2*°R)	No	5.678263340863113 kg/(s^3*K)
calories	cal	No	4.1868 m^2*kg/s^2
calories per gram per kelvin	cal/(g*K)	No	4186.8 m^2/(s^2*K)
calories per hour	cal/h	No	0.001163 m^2*kg/s^3
calories per hour per kelvin	cal/(h*K)	No	0.001163 m^2*kg/(s^3*K)
calories per hour per meter per kelvin	cal/(h*m*K)	No	0.001163 m*kg/(s^3*K)
calories per hour per square meter per kelvin	cal/(h*m^2*K)	No	0.001163 kg/(s^3*K)
calories per minute	cal/min	No	0.06978 m^2*kg/s^3
calories per mole	cal/mol	No	4.1868 m^2*kg/(s^2*mol)
calories per second	cal/s	No	4.1868 m^2*kg/s^3
candelas	cd	Yes	1 cd
candelas per candela	cd/cd	Yes	1 cd/cd
candela steradians	cd*sr	Yes	1 m^2*cd/m^2
celsius	°C	No	1 K
celsius per meter	°C/m	No	1 K/m
centimeter squared gram	cm^2*g	No	1E-07 m^2*kg
centimeter squared kilogram	cm^2*kg	No	0.0001 m^2*kg
centuries	hyr	No	3155760000 s
chains	ch	No	20.1168 m
characteristic impedance of vacuum	Z_0	No	376.7303134617707 m^2*kg/(s^3*A^2)
charge density	A*h/L	No	3600000 s*A/m^3
compton wavelengths	λ_C	No	2.42631023609262E-12 m
conductance quantum	G_0	No	7.748091730820603E-05 s^3*A^2/(m^2*kg)
coulombs	C	Yes	1 s*A
coulomb meters	C*m	Yes	1 m*s*A
coulombs per cubic meter	C/m^3	Yes	1 s*A/m^3
coulombs per kilogram	C/kg	Yes	1 s*A/kg
coulombs per mole	C/mol	No	1 s*A/mol
coulombs per square meter	C/m^2	Yes	1 s*A/m^2
coulombs per volt meter	C/(V*m)	Yes	1 s^4*A^2/(m^3*kg)
cubic centimeters per hour	cm^3/h	No	2.777777777777778E-10 m^3/s
cubic centimeters per minute	cm^3/min	No	1.666666666666667E-08 m^3/s
cubic centimeters per mole	cm^3/mol	No	1E-06 m^3/mol
cubic centimeters per second	cm^3/s	No	1E-06 m^3/s
cubic feet	ft^3	No	0.028316846592 m^3
cubic feet per hour	ft^3/h	No	7.86579072E-06 m^3/s
cubic feet per minute	ft^3/min	No	0.0004719474432 m^3/s
cubic feet per second	ft^3/s	No	0.028316846592 m^3/s
cubic inches	in^3	No	1.6387064E-05 m^3
cubic meters per cubic meter	m^3/m^3	No	1 m^3/m^3
cubic meters per hour	m^3/h	No	0.0002777777777777778 m^3/s

Unit or constant name	Symbol	SI Prefix	Value in Coherent Derived SI Unit
cubic meters per kilogram	m^3/kg	No	1 m^3/kg
cubic meters per minute	m^3/min	No	0.0166666666666667 m^3/s
cubic meters per mole	m^3/mol	No	1 m^3/mol
cubic meters per second	m^3/s	No	1 m^3/s
cubic yards	yd^3	No	0.764554857984 m^3
cubic ångströms	Å^3	No	1E-30 m^3
cups	cup	No	0.0002365882365 m^3
curies	Ci	Yes	37000000000 (1/s)
daltons	Da	Yes	1.66053904E-27 kg
darcys	Dc	No	9.869233E-13 m^2
days	d	No	86400 s
debyes	D	No	3.335640951816991E-30 m*s*A
decades	dayr	No	315576000 s
degrees	°	No	0.0174532925199433 m/m
drams	dr	No	0.0017718451953125 kg
dynes	dyn	Yes	1E-05 m*kg/s^2
dynes per centimeter	dyn/cm	No	0.001 kg/s^2
dynes per square centimeter	dyn/cm^2	No	0.1 m^2*kg/(m^2*s^2)
electric constant	ε_0	No	8.854187817620413E-12 s^4*A^2/(m^3*kg)
electron g factor	g_e	No	-2.00231930436182 m^2*A/(m^2*A)
electron magnetic moment	μ_e	No	-9.28476462E-24 m^2*A
electron mass	m_e	No	9.10938356E-31 kg
electronvolts	eV	Yes	1.6021766208E-19 m^2*kg/s^2
elementary charge	q_e	No	1.6021766208E-19 s*A
ergs	erg	Yes	1E-07 m^2*kg/s^2
ergs per second	erg/s	No	1E-07 m^2*kg/s^3
euler constant	e	No	2.718281828459045
fahrenheit	°F	No	0.555555555555556 K
fahrenheit per foot	°F/ft	No	1.822688830562848 K/m
farads	F	Yes	1 s^4*A^2/(m^2*kg)
farads per meter	F/m	Yes	1 s^4*A^2/(m^3*kg)
faraday constant	&F	No	96485.33288249877 s*A/mol
fathoms	ftm	No	1.8288 m
fine structure constant	α	No	0.007297352566206478 m^5*kg*s^4*A^2/(m^5*kg*s^4*A^2)
fluid ounces	floz	No	2.95735295625E-05 m^3
feet	ft	No	0.3048 m
feet per hour	ft/h	No	8.466666666666667E-05 m/s
feet per minute	ft/min	No	0.00508 m/s
feet per second	ft/s	No	0.3048 m/s
feet per square minute	ft/min^2	No	8.466666666666667E-05 m/s^2
feet per square second	ft/s^2	No	0.3048 m/s^2
feet pound force	ft*lbf	No	1.3558179483314 m^2*kg^2/(kg*s^2)
feet pound force per hour	ft*lbf/h	No	0.0003766160967587223 m^2*kg/s^3
feet pound force per minute	ft*lbf/min	No	0.02259696580552334 m^2*kg/s^3
feet pound force per second	ft*lbf/s	No	1.3558179483314 m^2*kg/s^3
furlongs	fur	No	201.168 m
gallons	gal	No	0.003785411784 m^3
gallons per hour	gal/h	No	1.051503273333333E-06 m^3/s

Unit or constant name	Symbol	SI Prefix	Value in Coherent Derived SI Unit
gallons per minute	gal/min	No	6.30901964E-05 m^3/s
gallons per second	gal/s	No	0.003785411784 m^3/s
gas constant	R	No	8.314459861448581 m^2*kg/(s^2*K*mol)
gas permeance unit	GPU	No	0.33 m*s^2*mol/(m^2*kg*s)
gauss	G	Yes	0.0001 kg/(s^2*A)
gauss per centimeter	G/cm	No	0.01 kg/(m*s^2*A)
gill	gi	No	0.00011829411825 m^3
grains	gr	No	6.479891E-05 kg
grams	g	Yes	0.001 kg
gram meters per second	m*g/s	Yes	0.001 m*kg/s
grams per cubic meter	g/m^3	Yes	0.001 kg/m^3
grams per kilogram	g/kg	Yes	0.001 kg/kg
grams per liter	g/L	Yes	1 kg/m^3
grams per microliter	g/µL	Yes	1000000 kg/m^3
grams per milliliter	g/mL	Yes	1000 kg/m^3
grams per mole	g/mol	Yes	0.001 kg/mol
grams per second	g/s	Yes	0.001 kg/s
grams per square meter	g/m^2	Yes	0.001 kg/m^2
grams per square meter per second	g/(m^2*s)	Yes	0.001 kg/(m^2*s)
gram square meters per second	g*m^2/s	Yes	0.001 m^2*kg/s
gravitational constant	G_N	No	6.67408E-11 m^3/(kg*s^2)
gravity acceleration	g_0	No	9.80665 m/s^2
grays	Gy	Yes	1 m^2*kg/(kg*s^2)
grays per second	Gy/s	Yes	1 m^2/s^3
half teaspoons	halftsp	No	2.464460796875E-06 m^3
hectares	ha	No	10000 m^2
henries	H	Yes	1 m^2*kg/(s^2*A^2)
henries per meter	H/m	Yes	1 m*kg/(s^2*A^2)
hertz	Hz	Yes	1 (1/s)
hertz per atomic unit of electric field gradient	Hz/Λ_0	No	1.029085839028452E-22 m^2*s^3*A/(m^2*kg*s)
hertz per atomic unit of electric field gradient squared	Hz/Λ_0^2	No	1.059017664088893E-44 m^4*s^6*A^2/(m^4*kg^2*s)
hertz per hertz	Hz/Hz	Yes	1 s/s
hertz per tesla	Hz/T	Yes	1 s*A/kg
horse power per ounce	hp/oz	No	26303.78891073498 m^2*kg/(kg*s^3)
horse power per pound	hp/lb	No	1643.986806920936 m^2*kg/(kg*s^3)
horsepower	hp	No	745.699872 m^2*kg/s^3
hours	h	No	3600 s
hundredweight	cwt	No	45.359237 kg
hundredweightUK	cwtUK	No	50.80234544 kg
imperial cups	cupUK	No	0.000284130625 m^3
imperial fluid ounces	flozUK	No	2.84130625E-05 m^3
imperial gallons	galUK	No	0.00454609 m^3
imperial gallons per hour	galUK/h	No	1.262802777777778E-06 m^3/s
imperial gallons per minute	galUK/min	No	7.576816666666667E-05 m^3/s
imperial gallons per second	galUK/s	No	0.00454609 m^3/s
imperial gill	giUK	No	0.0001420653125 m^3
imperial half teaspoons	halftspUK	No	2.959694010416667E-06 m^3
imperial pints	ptUK	No	0.00056826125 m^3

Unit or constant name	Symbol	SI Prefix	Value in Coherent Derived SI Unit
imperial quarts	qtUK	No	0.0011365225 m^3
imperial quarter teaspoons	quartertspUK	No	1.479847005208333E-06 m^3
imperial tablespoons	tbspUK	No	1.77581640625E-05 m^3
imperial teaspoons	tspUK	No	5.919388020833334E-06 m^3
inches	in	No	0.0254 m
inch ounce force	in*ozf	No	0.007061551814226041 m^2*kg^2/(kg*s^2)
inches per hour	in/h	No	7.055555555555556E-06 m/s
inches per minute	in/min	No	0.0004233333333333334 m/s
inches per second	in/s	No	0.0254 m/s
inches per square minute	in/min^2	No	7.055555555555557E-06 m/s^2
inches per square second	in/s^2	No	0.0254 m/s^2
inch pound force	in*lbf	No	0.1129848290276167 m^2*kg^2/(kg*s^2)
inches pound force per hour	in*lbf/h	No	3.138467472989352E-05 m^2*kg/s^3
inches pound force per minute	in*lbf/min	No	0.001883080483793611 m^2*kg/s^3
inches pound force per second	in*lbf/s	No	0.1129848290276167 m^2*kg/s^3
joules	J	Yes	1 m^2*kg/s^2
joules per cubic meter	J/m^3	Yes	1 kg/(m*s^2)
joules per gram	J/g	Yes	1000 m^2/s^2
joules per gram kelvin	J/(g*K)	Yes	1000 m^2/(s^2*K)
joules per kelvin	J/K	Yes	1 m^2*kg/(s^2*K)
joules per kilogram	J/kg	Yes	1 m^2/s^2
joules per kilogram kelvin	J/(kg*K)	Yes	1 m^2/(s^2*K)
joules per liter	J/L	Yes	1000 kg/(m*s^2)
joules per mole	J/mol	Yes	1 m^2*kg/(s^2*mol)
joules per mole kelvin	J/(mol*K)	Yes	1 m^2*kg/(s^2*K*mol)
joules per nanometer	J/nm	Yes	1E+09 m^2*kg/(m*s^2)
joules per radian	J/rad	Yes	1 m^3*kg/(m*s^2)
Joules per second	J/s	Yes	1 m^2*kg/s^3
joules per square meter	J/m^2	Yes	1 m^2*kg/(m^2*s^2)
joules per tesla	J/T	Yes	1 m^2*A
joules second	J*s	Yes	1 m^2*kg/s
katals	kat	Yes	1 mol/s
katals per cubic meter	kat/m^3	Yes	1 mol/(m^3*s)
katals per kilogram	kat/kg	Yes	1 mol/(kg*s)
katals per liter	kat/L	Yes	1000 mol/(m^3*s)
kelvin	K	Yes	1 K
kelvin per kelvin	K/K	Yes	1 K/K
kelvin per meter	K/m	No	1 K/m
kilocalories	kcal	No	4186.8 m^2*kg/s^2
kilocalories per hour per kelvin	kcal/(h*K)	No	1.163 m^2*kg/(s^3*K)
kilocalories per hour per meter per kelvin	kcal/(h*m*K)	No	1.163 m*kg/(s^3*K)
kilocalories per hour per square meter per kelvin	kcal/(h*m^2*K)	No	1.163 kg/(s^3*K)
kilocalories per mole	kcal/mol	No	4186.8 m^2*kg/(s^2*mol)
kilograms force	kgf	No	9.80665 m*kg/s^2
kilogram force centimeters per radian	kgf*cm/rad	No	0.0980665 m^3*kg/(m*s^2)
kilogram force meters per radian	kgf*m/rad	No	9.80665 m^3*kg/(m*s^2)
kilohertz per atomic unit of electric field gradient	kHz/Λ_0	No	1.029085839028452E-19 m^2*s^3*A/(m^2*kg*s)
kilohertz per atomic unit of electric field gradient squared	kHz/Λ_0^2	No	1.059017664088893E-41 m^4*s^6*A^2/(m^4*kg^2*s)

Unit or constant name	Symbol	SI Prefix	Value in Coherent Derived SI Unit
knots	kn	No	0.514444444444444 m/s
knots per second	kn/s	No	0.514444444444444 m/s^2
leagues	lea	No	4828.032 m
light years	ly	No	9460730472580800 m
links	li	No	0.201168 m
liters	L	Yes	0.001 m^3
liters per 100 kilometers	L/(100 km)	No	1E-08 m^3/m
liter per mole per second	L/(mol*s)	No	0.001 m^3/(s*mol)
lumens	lm	Yes	1 m^2*cd/m^2
lumens per square foot	lm/ft^2	No	10.76391041670972 m^2*cd/m^4
lumens per square meter	lm/m^2	Yes	1 m^2*cd/m^4
lumens per square meter per steradian	lm/(m^2*sr)	Yes	1 cd/m^2
lumens per watt	lm/W	Yes	1 s^3*cd/(m^2*kg)
lumen seconds	lm*s	Yes	1 m^2*s*cd/m^2
lux	lx	Yes	1 m^2*cd/m^4
magnetic constant	µ_0	No	1.256637061435917E-06 m*kg/(s^2*A^2)
magnetic flux quantum	Φ_0	No	2.067833831170082E-15 m^2*kg/(s^2*A)
maxwells	Mx	Yes	1E-08 m^2*kg/(s^2*A)
megahertz per atomic unit of electric field gradient	MHz/Λ_0	No	1.029085839028452E-16 m^2*s^3*A/(m^2*kg*s)
megahertz per atomic unit of electric field gradient squared	MHz/Λ_0^2	No	1.059017664088893E-38 m^4*s^6*A^2/(m^4*kg^2*s)
meters	m	Yes	1 m
meters per hour	m/h	Yes	0.000277777777777778 m/s
meters per hour per second	m/(h*s)	Yes	0.000277777777777778 m/s^2
meters per liter	m/L	Yes	1000 m/m^3
meters per meter	m/m	Yes	1 m/m
meters per minute	m/min	Yes	0.0166666666666667 m/s
meters per second	m/s	Yes	1 m/s
meters per square second	m/s^2	Yes	1 m/s^2
meter seconds per meter second	m*s/(m*s)	No	1 m*s/(m*s)
meters squared gram	m^2*g	No	0.001 m^2*kg
meters squared kilogram	m^2*kg	No	1 m^2*kg
microdarcys	µDc	No	9.869233E-19 m^2
micrograms	mcg	No	1E-09 kg
miles	mi	No	1609.344 m
miles per gallon	mi/gal	No	425143.707430272 m/m^3
miles per hour	mi/h	No	0.44704 m/s
miles per hour per second	mi/(h*s)	No	0.44704 m/s^2
miles per imperial gallon	mi/galUK	No	354006.1899346471 m/m^3
miles per minute	mi/min	No	26.8224 m/s
miles per second	mi/s	No	1609.344 m/s
miles per square minute	mi/min^2	No	0.44704 m/s^2
miles per square second	mi/s^2	No	1609.344 m/s^2
millennia	kyr	No	31557600000 s
milliampere hours	mA*h	No	3.6 s*A
milliampere hours per gram	mA*h/g	No	3600 s*A/kg
milliampere minutes	mA*min	No	0.06 s*A
milliampere minutes per gram	mA*min/g	No	60 s*A/kg
milliampere seconds	mA*s	No	0.001 s*A

Unit or constant name	Symbol	SI Prefix	Value in Coherent Derived SI Unit
milliampere seconds per gram	mA*s/g	No	1 s*A/kg
millidarcys	mDc	No	9.869233E-16 m^2
millimeters of Hg	mmHg	No	133.322 kg/(m*s^2)
million oil barrels	MMbbl	No	158987.295 m^3
minutes	min	No	60 s
moles	mol	Yes	1 mol
moles per cubic meter	mol/m^3	Yes	1 mol/m^3
moles per kilogram	mol/kg	Yes	1 mol/kg
moles per liter	mol/L	Yes	1000 mol/m^3
moles per liter	M	Yes	1000 mol/m^3
moles per microliter	mol/µL	Yes	1E+09 mol/m^3
moles per milliliter	mol/mL	Yes	1000000 mol/m^3
moles per minute	mol/min	Yes	0.0166666666666667 mol/s
moles per mole	mol/mol	Yes	1 mol/mol
moles per second	mol/s	Yes	1 mol/s
months	month	No	2629800 s
muon g factor	g_µ	No	-2.0023318418 m^2*A/(m^2*A)
muon magnetic moment	µ_µ	No	-4.49044826E-26 m^2*A
myon mass	m_µ	No	1.883531594E-28 kg
nanodarcys	nDc	No	9.869233E-21 m^2
natural units of energy	m_e*c_0^2	No	8.187105649650028E-14 m^2*kg/s^2
natural units of length	ƛ_C	No	3.8615926764E-13 m
natural units of momentum	m_e*c_0	No	2.73092448831719E-22 m*kg/s
natural units of time	ℏ/(m_e*c_0^2)	No	1.288088666822313E-21 s
newtons per meter	N/m	Yes	1 kg/s^2
newtons per square meter	N/m^2	Yes	1 kg/(m*s^2)
neutron g factor	g_n	No	-3.82608545 m^2*A/(m^2*A)
neutron magnetic moment	µ_n	No	-9.662365E-27 m^2*A
neutron mass	m_n	No	1.674927471E-27 kg
newtons	N	Yes	1 m*kg/s^2
newton meters	N*m	Yes	1 m^2*kg^2/(kg*s^2)
newton meters per radian	N*m/rad	Yes	1 m^3*kg/(m*s^2)
newtons per coulomb	N/C	Yes	1 m*kg/(s^3*A)
newtons per square ampere	N/A^2	Yes	1 m*kg/(s^2*A^2)
newton seconds	N*s	Yes	1 m*kg/s
newton seconds per square meter	N*s/m^2	Yes	1 kg/(m*s)
nuclear magnetons	µ_N	No	5.050783698211084E-27 m^2*A
ohms	Ω	Yes	1 m^2*kg/(s^3*A^2)
ohms centimeter	Ω*cm	Yes	0.01 m^3*kg/(s^3*A^2)
ohms meter	Ω*m	Yes	1 m^3*kg/(s^3*A^2)
ohms per feet	Ω/ft	Yes	3.280839895013123 m^2*kg/(m*s^3*A^2)
ohms per meter	Ω/m	Yes	1 m^2*kg/(m*s^3*A^2)
oil barrels	bbl	No	0.158987295 m^3
ounces	oz	No	0.028349523125 kg
ounces force	ozf	No	0.2780138509537812 m*kg/s^2
ounce force inches	ozf*in	No	0.007061551814226041 m^2*kg^2/(kg*s^2)
parts per billion	ppb	No	1E-09
parts per million	ppm	No	1E-06

Unit or constant name	Symbol	SI Prefix	Value in Coherent Derived SI Unit
parts per quadrillion	ppq	No	1E-15
parts per ten thousand	‱	No	0.0001
parts per thousand	‰	No	0.001
parts per trillion	ppt	No	1E-12
pascals	Pa	Yes	1 kg/(m*s^2)
pascals per meter	Pa/m	Yes	1 kg/(m^2*s^2)
pascal seconds	Pa*s	Yes	1 kg/(m*s)
percent	%	No	0.01
phots	ph	Yes	10000 m^2*cd/m^4
pi	π	No	3.141592653589793 m/m
pints	pt	No	0.000473176473 m^3
planck charge	q_P	No	1.875546022722158E-18 s*A
planck constant	h_P	No	6.62607004E-34 m^2*kg/s
planck length	l_P	No	1.616228373080886E-35 m
planck mass	m_P	No	2.176470195634196E-08 kg
planck temperature	T_P	No	1.416807993748162E+32 K
planck time	t_P	No	5.391157549003072E-44 s
poises	P	Yes	0.1 kg/(m*s)
pounds	lb	No	0.45359237 kg
pounds force	lbf	No	4.4482216152605 m*kg/s^2
pound force feet	lbf*ft	No	1.3558179483314 m^2*kg^2/(kg*s^2)
pound force feet per radian	lbf*ft/rad	No	1.3558179483314 m^3*kg/(m*s^2)
pound force inches	lbf*in	No	0.1129848290276167 m^2*kg^2/(kg*s^2)
pound force inches per radian	lbf*in/rad	No	0.1129848290276167 m^3*kg/(m*s^2)
pounds force per square feet	lbf/ft^2	No	47.880259 kg/(m*s^2)
pounds force per square inch	psi	No	6894.75729 kg/(m*s^2)
pounds force per square inch	lbf/in^2	No	6894.75729 kg/(m*s^2)
pounds force per square inch per foot	psi/ft	No	22620.59478346456 kg/(m^2*s^2)
proton g factor	g_p	No	5.585694702 m^2*A/(m^2*A)
proton magnetic moment	μ_p	No	1.4106067873E-26 m^2*A
proton mass	m_p	No	1.672621898E-27 kg
quantum of circulation	h_P/(2*m_e)	No	1.054571800139113E-34 m^2/s
quarts	qt	No	0.000946352946 m^3
quarter teaspoons	quartertsp	No	1.2322303984375E-06 m^3
radians	rad	Yes	1 m/m
radians per second	rad/s	Yes	1 m/(m*s)
radians per second per tesla	rad/(s*T)	Yes	1 m*s^2*A/(m*kg*s)
radians per square second	rad/s^2	Yes	1 m/(m*s^2)
rankines	°R	No	0.5555555555555556 K
rankines per foot	°R/ft	No	1.822688830562848 K/m
reduced planck constant	ℏ	No	1.054571800139113E-34 m^3*kg/(m*s)
rods	rod	No	5.0292 m
rydbergs	Ry	No	2.179872325390242E-18 m^2*kg/s^2
rydberg constant	R_∞	No	10973731.5705508 (1/m)
seconds	s	Yes	1 s
seconds per second	s/s	Yes	1 s/s
second radiation constant	h_P*c_0/k_B	No	0.0143877735382772 m^3*kg*s^2*K/(m^2*kg*s^2)
siemens	S	Yes	1 s^3*A^2/(m^2*kg)

Unit or constant name	Symbol	SI Prefix	Value in Coherent Derived SI Unit
siemens centimeter squared per mole	S*cm^2/mol	Yes	0.0001 m^2*s^3*A^2/(m^2*kg*mol)
siemens meter squared per mole	S*m^2/mol	Yes	1 m^2*s^3*A^2/(m^2*kg*mol)
siemens per centimeter	S/cm	Yes	100 s^3*A^2/(m^3*kg)
siemens per meter	S/m	Yes	1 s^3*A^2/(m^3*kg)
sieverts	Sv	Yes	1 m^2*kg/(kg*s^2)
speed of light	c_0	No	299792458 m/s
ångström ångströms	Å^2	No	1E-20 m^2
square centimeters	cm^2	No	0.0001 m^2
square centimeters per second	cm^2/s	No	0.0001 m^2/s
square chains	ch^2	No	404.6872515856 m^2
square feet	ft^2	No	0.09290304 m^2
square inches	in^2	No	0.00064516 m^2
square kilometers	km^2	No	1000000 m^2
square meters	m^2	No	1 m^2
square meters per cubic meter	m^2/m^3	No	1 m^2/m^3
square meters per liter	m^2/L	No	1000 m^2/m^3
square meters per second	m^2/s	No	1 m^2/s
square meters per square meter	m^2/m^2	No	1 m^2/m^2
square micrometers	µm^2	No	1E-12 m^2
square micrometers per second	µm^2/s	No	1E-12 m^2/s
square miles	mi^2	No	2589988.110336 m^2
square millimeters	mm^2	No	1E-06 m^2
square millimeters per second	mm^2/s	No	1E-06 m^2/s
square nanometers	nm^2	No	1E-18 m^2
square rods	rod^2	No	25.2929532241 m^2
square yards	yd^2	No	0.83612736 m^2
stefan-boltzmann constant	σ	No	5.670367E-08 m^2*kg/(m^2*s^3*K^4)
steradians	sr	Yes	1 m^2/m^2
stilbs	sb	Yes	10000 cd/m^2
stokes	St	Yes	0.0001 m^2/s
stones	st	No	6.35029318 kg
tablespoons	tbsp	No	1.478676478125E-05 m^3
teaspoons	tsp	No	4.92892159375E-06 m^3
tesla	T	Yes	1 kg/(s^2*A)
tesla meter per ampere	T*m/A	Yes	1 m*kg/(s^2*A^2)
tesla per centimeter	T/cm	No	100 kg/(m*s^2*A)
tesla per meter	T/m	Yes	1 kg/(m*s^2*A)
thomson	Th	No	1.036426957204542E-08 kg/(s*A)
thousand oil barrels	Mbbl	No	158.987295 m^3
tons	ton	No	907.18474 kg
tonnes	t	No	1000 kg
tonsUK	tonUK	No	1016.0469088 kg
torrs	Torr	No	133.3223684210526 kg/(m*s^2)
townships	twp	No	93239571.972096 m^2
turns	tr	Yes	6.283185307179586 m/m
volts	V	Yes	1 m^2*kg/(s^3*A)
volts meter	V*m	Yes	1 m^3*kg/(s*A)
volts per centimeter	V/cm	Yes	100 m*kg/(s^3*A)

Unit or constant name	Symbol	SI Prefix	Value in Coherent Derived SI Unit
volts per meter	V/m	Yes	1 m*kg/(s^3*A)
volts per square meter	V/m^2	Yes	1 m^2*kg/(m^2*s^3*A)
von klitzing constant	h_P/(q_e^2)	No	25812.80745611641 m^2*kg/(s^3*A^2)
watts	W	Yes	1 m^2*kg/s^3
watt hour	W*h	Yes	3600 m^2*kg/s^2
watts per cubic centimeter	W/cm^3	Yes	100000 m^2*kg/(m^3*s^3)
watts per cubic meter	W/m^3	Yes	1 m^2*kg/(m^3*s^3)
watts per kelvin	W/K	Yes	1 m^2*kg/(s^3*K)
watts per kilogram	W/kg	Yes	1 m^2*kg/(kg*s^3)
watts per lumen	W/lm	Yes	1 m^3*kg/(m*s^3*cd)
watts per meter kelvin	W/(m*K)	Yes	1 m*kg/(s^3*K)
watts per nanometer	W/nm	Yes	1E+09 m^2*kg/(m*s^3)
watts per square centimeter	W/cm^2	Yes	10000 m^2*kg/(m^2*s^3)
watts per square foot	W/ft^2	No	10.76391041670972 m^2*kg/(m^2*s^3)
watts per square inch	W/in^2	No	0.89699253472581 m^2*kg/(m^2*s^3)
watts per square meter	W/m^2	Yes	1 m^2*kg/(m^2*s^3)
watts per square meter per kelvin	W/(m^2*K)	Yes	1 kg/(s^3*K)
watts per square meter per nanometer	W/(m^2*nm)	Yes	1E+09 kg/(m*s^3)
watts per square meter per steradian	W/(m^2*sr)	Yes	1 m^4*kg/(m^4*s^3)
watts per square meter steradian per nanometer	W/(m^2*sr*nm)	Yes	1E+09 m^4*kg/(m^5*s^3)
watts per steradian	W/sr	Yes	1 m^4*kg/(m^2*s^3)
watts per steradian per nanometer	W/(sr*nm)	Yes	1E+09 m^4*kg/(m^3*s^3)
webers	Wb	Yes	1 m^2*kg/(s^2*A)
webers per ampere meter	Wb/(A*m)	Yes	1 m*kg/(s^2*A^2)
weeks	wk	No	604800 s
wien wavelength displacement constant	b_λ	No	0.0028977729 m*K
yards	yd	No	0.9144 m
years	yr	No	31557600 s
ångströms	Å	No	1E-10 m
œrsteds	Oe	Yes	79.57747154594767 A/m
